# BRD4 prevents the accumulation of R-loops and protects against transcription–replication collision events and DNA damage

**DOI:** 10.1038/s41467-020-17503-y

**Published:** 2020-08-14

**Authors:** Fred C. Lam, Yi Wen Kong, Qiuying Huang, Tu-Lan Vu Han, Amanda D. Maffa, Ekkehard M. Kasper, Michael B. Yaffe

**Affiliations:** 1 Koch Institute for Integrative Cancer Research, Massachusetts Institute of Technology, 500 Main Street, Cambridge, MA 02139 USA; 2 Center for Precision Cancer Medicine, Massachusetts Institute of Technology, 500 Main Street, Cambridge, MA 02139 USA; 3 Faculty of Health Sciences, Division of Neurosurgery, Hamilton General Hospital, McMaster University, 237 Barton St E, Hamilton, ON L8L 2X2 Canada; 4 Departments of Biology and Bioengineering, Massachusetts Institute of Technology, Cambridge, MA 02139 USA; 5 Beth Israel Deaconess Medical Center, Department of Surgery, Harvard Medical School, Boston, MA 02215 USA

**Keywords:** Cancer therapy, DNA damage checkpoints, DNA synthesis, Transcription

## Abstract

Proper chromatin function and maintenance of genomic stability depends on spatiotemporal coordination between the transcription and replication machinery. Loss of this coordination can lead to DNA damage from increased transcription-replication collision events. We report that deregulated transcription following BRD4 loss in cancer cells leads to the accumulation of RNA:DNA hybrids (R-loops) and collisions with the replication machinery causing replication stress and DNA damage. Whole genome BRD4 and γH2AX ChIP-Seq with R-loop IP qPCR reveals that BRD4 inhibition leads to accumulation of R-loops and DNA damage at a subset of known BDR4, JMJD6, and CHD4 co-regulated genes. Interference with BRD4 function causes transcriptional downregulation of the DNA damage response protein TopBP1, resulting in failure to activate the ATR-Chk1 pathway despite increased replication stress, leading to apoptotic cell death in S-phase and mitotic catastrophe. These findings demonstrate that inhibition of BRD4 induces transcription-replication conflicts, DNA damage, and cell death in oncogenic cells.

## Abstract (web summary)

In order to avoid transcription-replication conflicts (TRCs) on shared DNA templates, cell must maintain strict spatiotemporal co-ordination of transcription with replication. Here the authors uncover a role for BRD4 in preventing TRCs and DNA damage checkpoint signaling in oncogenic cells.

## Introduction

Maintaining genome integrity depends on coordination and cross-talk between processes that occur along chromatin. This is particularly relevant in eukaryotic cells, where multiple replication origins firing at different times can encounter chromatin occupied by transcription machinery. It is reported that both co-directional and bi-directional processes occurring on the same DNA template can cause transcription–replication conflicts (TRCs) and collision events leading to DNA damage and genome instability^1,2^. The inability of a cell to maintain strict spatiotemporal co-ordination of transcription with replication, or to deal with these conflicts, ultimately affects cell proliferation and viability.

One significant source of such collision events is the formation of R-loops, intermediates of transcription consisting of a complementary RNA-DNA hybrid plus a displaced single-stranded DNA^3^. R-loops can occur naturally during processes, such as mitochondrial DNA replication^4^, transcription-coupled recombination^5^, and immunoglobulin class-switching^6^. Common among these natural R-Loop phenomena is the concept that R-loops lead to productive chromosome rearrangements. However, abnormal R-loop accumulation causes DNA damage and genomic instability, presumably through increased collisions with replication forks^7–9^. Evidence implicating R-loops as a source of deleterious DNA damage first came from studies in yeast cells in which loss of function of genes involved in mRNA processing led to DNA damage and genome instability, which could be rescued upon overexpression of RNase H^5,8,9^. Further work has identified helicases such as AQR and SETX, and DNA damage response components such as BRCA1, BRCA2, Cockayne syndrome, and Fanconi Anemia proteins, in preventing R-loop-induced double-strand break (DSB) formation in human cells^10–14^ suggesting that proteins involved in chromatin modification and the DNA damage response are important for prevention and resolution of R-loop-induced DNA damage and intimating a role in oncogenesis.

Bromodomain-containing proteins (BRDs) are a class of transcriptional co-activators that recognize acetylated lysine residues on histones through direct binding to tandem conserved bromodomains. Upon binding to the chromatin, BRDs are known to function in the assembly of complexes that facilitate chromatin accessibility to transcription factors allowing for the recruitment of RNA polymerases^15,16^. The bromodomain and extraterminal domain (BET) family of BRDs (BRD2, BRD3, BRD4, and BRDT) share conserved bromodomains and an extraterminal (ET) domain, which has been shown to interact with several transcriptional co-activators^17^. The prototypical BET bromodomain family member BRD4, recruits the positive elongation factor (P-TEFb) complex along with transcriptional co-activators JMJD6 and CHD4 to assist RNA polymerase II (RNAPII) elongation^18^. In particular, BRD4 has been shown to be concentrated at super-enhancer regions upstream of the *MYC* promoter in oncogenic cells, making it an attractive target in multiple models of cancer^19,20^.

We previously reported a novel role for BRD4 in insulating the chromatin against radiation-induced DNA damage response signaling in oncogenic cells^21^. In the course of that study, we observed separately that in some cell types, BET bromodomain protein inhibition led to increased DNA damage signaling even in the absence of exogenous DNA damage sources. We noted that cell types with robust DNA damage responses to BET bromodomain inhibition alone were frequently oncogene-driven and rapidly proliferating, leading us to hypothesize that the mechanism of DNA damage involved both replication and the known role of BET bromodomain proteins in transcriptional regulation. Here, we report that BRD4 loss of function leads to the accumulation of R-loops in oncogenic cells causing increased transcription–replication collision events, DNA DSB formation, DNA damage response signaling, and apoptosis. R-loop-induced DNA damage could be reversed by overexpression of RNase H1 or by inhibiting the initiation of transcription using triptolide. These findings reveal the importance of BRD4 in preventing TRCs and regulating DNA damage checkpoint signaling in oncogenic cells.

## Results

### BRD4 bromodomain inhibition causes DNA damage and apoptosis

**Fig. 1:**
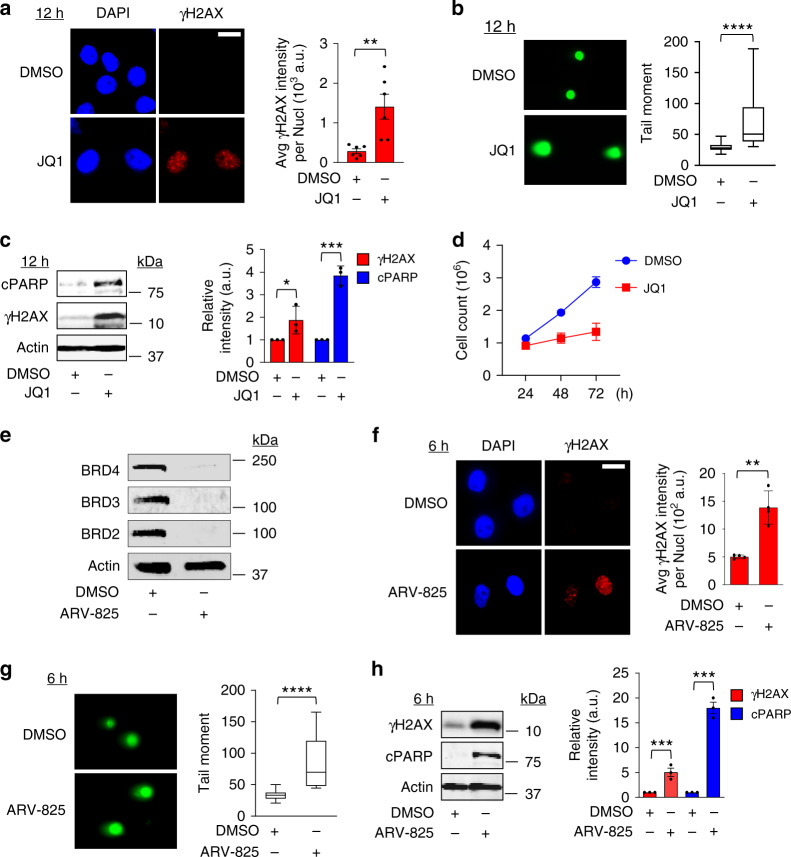
BRD4 bromodomain inhibition causes DNA damage and apoptosis. **a** Left panel: Immuno-fluorescence (IF) images of γH2AX fluorescence in HeLa cells following treatment with DMSO or 500 nM JQ1 for 12 h (*n* = 3 independent experiments). Significance assessed using two-tailed unpaired *t* test (**P = 0.0058). **b** Fluorescence microscopy images of neutral comet single cell gel electrophoresis assay in cells treated for 12 h with DMSO or JQ1 (*n* = 3). Box-whisker plots indicate median, 25th to 75th percentile, and maximum and minimum values by line, box, and whiskers, respectively. Significance assessed using two-tailed unpaired *t* test (****P < 0.0001). **c** Left panel: lysates from control or JQ1-treated cells were analyzed by western blotting (WB) for γH2AX and cleaved PARP (cPARP). Actin or tubulin serves as loading controls in this and subsequent WB. Right panel: Relative γH2AX and cPARP band intensities were quantified (*n* = 3). Significance assessed using one-tailed unpaired *t* test (*P_γH2AX_ = 0.0347, ***P_cPARP_ = 0.0002). **d** HeLa cells were treated with either DMSO or JQ1 and their growth plotted over a course of 72 h (*n* = 3, error bars indicate SEM). **e** HeLa cells were treated with the BET bromodomain proteasome-targeting small molecule ARV-825 (100 nM) for 3 h, and lysates probed for levels of BRD2, BRD3, and BRD4 by WB. **f** Representative IF images of γH2AX foci in cells treated with ARV-825 for 6 h (n = 3). Significance assessed using one-tailed unpaired *t* test (**P = 0.0011). **g** Fluorescence microscopy images of neutral cell single cell gel electrophoresis assay of cells treated with ARV-825 for 6 h (*n* = 3 independent experiments). Box-whisker plots drawn as in **b**. Significance assessed using two-tailed unpaired *t* test (****P < 0.0001). **g** Lysates from cells treated with ARV-825 (100 nM for 6 h) were analyzed for γH2AX, cPARP and actin by WB. Intensities of the γH2AX and cPARP bands were quantified (*n* = 3). Significance assessed using two-tailed unpaired *t* test (***P_γH2AX_ = 0.0009, ***P_cPARP_ = 0.0001). In panels **a**, **c**, **f**, and **h** data are presented as mean ± SEM. Scale bars in **a**, **f** = 5 μm. Source data are provided as a Source data file.

To further explore our previous finding that BRD4 is involved in regulating the DNA damage response in oncogenic cells^21^, we treated cells with the prototypical BET bromodomain inhibitor JQ1^22^ and assayed for changes in DNA damage response signaling using immunofluorescence (IF) and western blotting for γH2AX, a marker of DNA damage signaling and DSB^23^. Treatment of HeLa cells with 500 nM JQ1 for 12 h led to increased nuclear γH2AX immunostaining (Fig. 1a). This increase in DNA damage signaling corresponded to increased DSB formation as quantified using the neutral comet single cell gel electrophoresis assay (Fig. 1b), increased cleavage of PARP (cPARP), an indicator of apoptosis (Fig. 1c), and subsequent growth inhibition (Fig. 1d). The increase in DNA damage signaling, DSB formation, apoptosis, and growth inhibition following treatment with JQ1 was also seen in HCT116 cells (Supplementary Fig. 1a–d).

It has recently been reported that bromodomain inhibitors such as JQ1 can lead to significant accumulation of BRD4 protein in the nucleus, requiring prolonged periods of treatment with higher concentrations of drug in order to achieve significant protein inhibition^24^. This may explain why cells required prolonged treatment with JQ1 (at least 12 h) before we observed increases in DNA damage and apoptosis. However, lengthy treatment with JQ1 leads to global changes in gene expression that could confound our ability to study the relationship between deregulated transcription following BRD4 inhibition and DNA damage signaling. To circumvent this, we employed the use of the small molecule inhibitor ARV-825, a new class of BET bromodomain degraders, heterobifunctional proteolysis targeting chimeras which recruit BET bromodomain proteins to the E3 ubiquitin ligase cereblon, leading to rapid and prolonged protein degradation after treatment^24^. Treatment of HeLa cells with 100 nM ARV-825 for 3 h led to potent degradation of BRD2, BRD3, and BRD4 (Fig. 1e), and markedly increased nuclear γH2AX immunostaining (Fig. 1f), DSB formation (Fig. 1g), and cPARP (Fig. 1h) by 6 h, similar to levels caused by 12 h of JQ1 treatment. This increase in DNA damage signaling, DBS formation, and apoptosis following treatment with ARV-825 was also seen in HCT116 cells (Supplementary Fig. 1e–h). The ability to detect increased DNA damage after more rapid loss of BET bromodomain proteins reduces the confounding effects from changes in gene expression that are seen using bromodomain inhibitors. Taken together, these data suggest that inhibition or degradation of BET bromodomain proteins such as BRD4 leads to increased DNA damage signaling, DSB formation, and apoptosis in cells.

### DNA damage caused by BRD4 degradation requires transcription

**Fig. 2:**
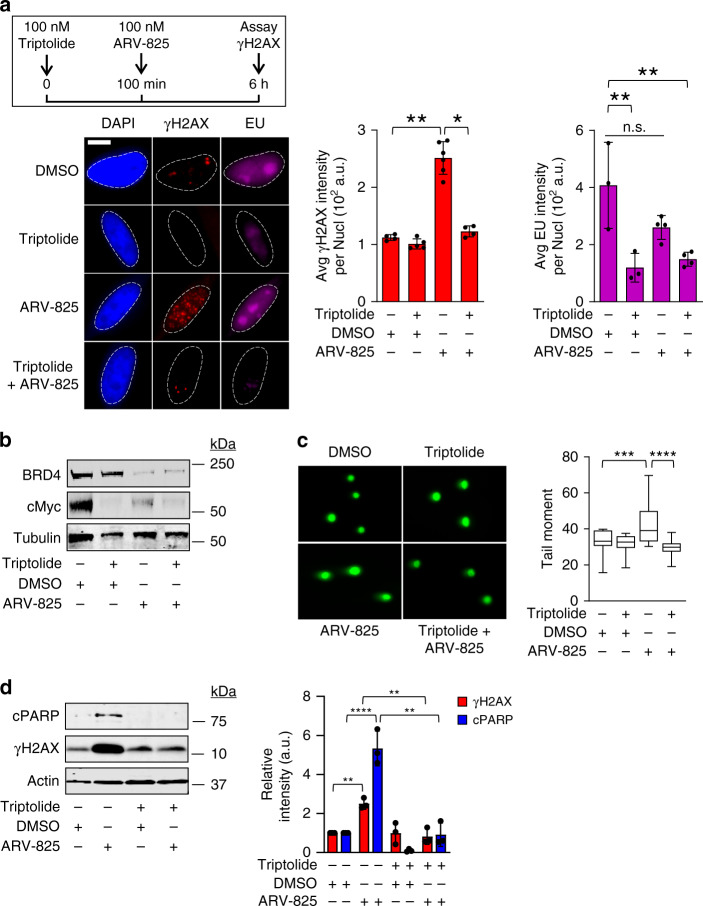
DNA damage caused by BRD4 degradation requires active transcription. **a** Top right: schematic of triptolide/ARV-825 co-treatment experiment in HeLa cells. Bottom right: representative IF images of HeLa cells following treatment with triptolide and/or ARV-825. EU incorporation was used to assess RNA synthesis. Left: Quantification of integrated γH2AX and EU intensity per nucleus quantified from >300 cells as in Fig. 1a (*n* = 3). Significance assessed using ANOVA followed by Tukey’s test (γH2AX_DMSO vs ARV-825_ **Adjusted *P* = 0.0038; γH2AX_Triptolide vs Triptolide + ARV_ *Adjusted *P* = 0.0139; EU_DMSO vs Triptolide_ **Adjusted *P* = 0.0041; EU_DMSO vs Triptolide + ARV-825_ **Adjusted *P* = 0.0055). Scale bar = 2.5 μm. **b** Lysates of cells treated with triptolide and/or ARV-825 were analyzed for BRD4 and cMyc levels by WB. **c** Representative fluorescence microscopy images of neutral comet single cell gel electrophoresis assay after triptolide and/or ARV-825 treatment. Box-whisker plots drawn as in Fig. 1b (*n* = 3). Significance assessed using ANOVA followed by Tukey’s test, ***Adjusted *P* = 0.0002, ****Adjusted *P* < 0.0001. **d** Lysates from cells pretreated with triptolide followed by co-treatment with ARV-825 were analyzed for γH2AX and cPARP by WB. Relative intensity of bands was quantified (*n* = 3). Significance assessed using ANOVA followed by Tukey’s test (γH2AX_DMSO vs ARV-825_ **Adjusted *P* = 0.0051; γH2AX_ARV-825 vs Triptolide + ARV-825_ **Adjusted *P* = 0.0019; cPARP_DMSO vs ARV-825_ ****Adjusted *P* < 0.0001; cPARP_ARV-825 vs Triptolide + ARV-825_ ****P < 0.0001). **b**-**d** are representative gels and images from the same experiment. Data in **a**, **d** presented as mean ± SEM. Source data are provided as a Source data file.

A principal member of BET bromodomain family of proteins, BRD4, is known to recruit the positive elongation factor P-TEFb, and to assist RNAPII in transcriptional elongation^16,25,26^. We hypothesized that the increased DNA damage signaling we observed following BET bromodomain protein loss could involve dysregulation of this transcriptional co-activator function. To test this, we pretreated cells briefly for 100 min with an inhibitor of transcriptional initiation, triptolide, a TFII helicase inhibitor that causes collapse of the transcription bubble and degradation of RNA polymerase II^27^, followed by a 6 h co-treatment with ARV-825, and assayed for changes in DNA damage signaling at the single cell level using γH2AX immunofluorescence, and in the bulk cell population using western blotting for γH2AX and cPARP. To assay for effectiveness of triptolide to inhibit transcription during this timeframe, we utilized ethyluridine (EU) incorporation to quantify de novo RNA synthesis in cells, along with western blotting for MYC protein levels, which has a rapid turnover time of <30 minutes^28,29^. HeLa cells treated with triptolide showed significantly decreased levels of EU incorporation (Fig. 2a) and suppression of MYC protein levels (Fig. 2b), demonstrating inhibition of transcription. Treatment with 100 μM triptolide did not significantly decrease BRD4 protein levels throughout the time course of the experiment, which was to be expected as BRD4 has been shown to have a half-life of ~18 h (Fig. 2b)^30^. Cells treated with ARV-825 showed marked degradation of BRD4 protein and significantly reduced levels of MYC protein, demonstrating reduced levels of BRD4-driven *MYC* transcription (Fig. 2b)^19^. Despite potent suppression of MYC, treatment with triptolide alone did not result in increased DNA damage signaling, DSBs, or apoptosis in cells, while treatment with ARV-825 alone was again associated with increased DNA damage, PARP-mediated apoptosis and DSB formation over the same time course (Fig. 2a, c, d). These findings suggest a mechanism of DNA damage and apoptosis induction following BRD4 loss that is independent of changes in MYC transcription alone, which has been reported as a predominant mechanism responsible for the decreased survival of oncogenic cells following treatment with BET bromodomain inhibitors^20^. Cells pretreated with triptolide followed by co-treatment with ARV-825 showed abrogation of DNA damage signaling, DSB formation, and apoptosis (Fig. 2a, c, d, respectively), suggesting that DNA damage caused by BRD4 loss requires the presence of active transcription bubbles. Abrogation of BET bromodomain degrader-induced DNA damage and DSB formation by triptolide was also seen in HCT116 cells (Supplementary Fig. 2a, b, respectively). It should be noted that the short course of RNAPII inhibition by triptolide treatment allowed us to avoid the increases in DNA damage and apoptosis that would have resulted from longer treatment times with triptolide, which would have confounded our results. Taken together, these data suggest that DNA damage and apoptosis following BET bromodomain protein degradation requires the presence of active transcription bubbles.

### DNA damage caused by BRD4 loss involves R-loop accumulation

**Fig. 3:**
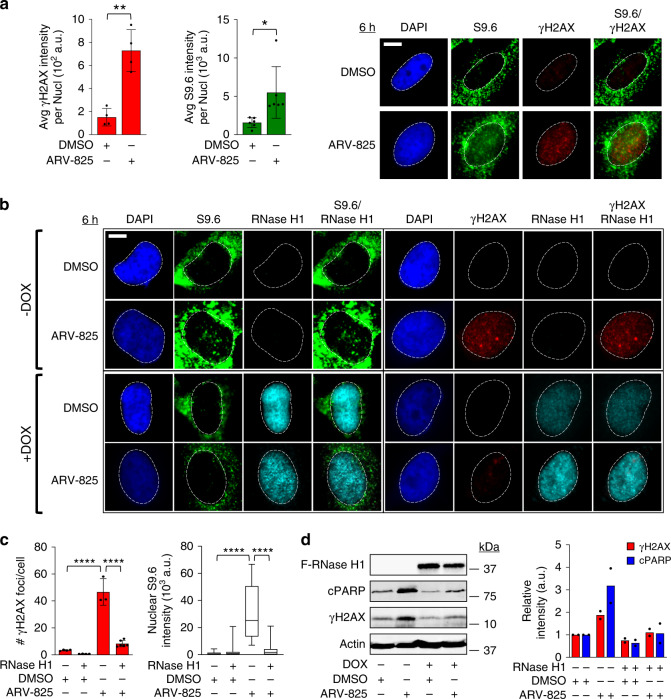
DNA damage caused by BRD4 loss requires R-loop accumulation. **a** Quantification and representative IF images of nuclear S9.6 and γH2AX intensity in HeLa cells following treatment with ARV-825 (100 nM for 6 h). Quantification was performed as described in Fig. 1a. For specific quantification of nuclear S9.6 staining, regions of interest were overlaid on the DAPI signal (delineated by dotted white lines in the figures) and selectively quantified to exclude cytoplasmic S9.6 signal and nucleolar S9.6 signal (*n* = 3 independent experiments). Statistical significance assessed using two-tailed unpaired *t* test (γH2AX_DMSO vs ARV-825_ **P = 0.0010; S9.6_DMSO vs ARV-825_ *P = 0.0186). **b**, **c** Representative IF images and quantification of nuclear S9.6 intensity, γH2AX foci, and RNase H1 in HeLa cells in the absence (−DOX) or presence (+DOX) of doxycycline-induced FLAG-tagged RNase H1 expression for 24 h, followed by treatment with 100 nM ARV-825 for 6 h (*n* = 3 independent experiments). Box-whisker plots are drawn as in Fig. 1b. Significance assessed using ANOVA followed by Tukey’s test ****Adjusted *P* < 0.0001. **d** Lysates from cells treated as in panel B were assayed for FLAG-tagged RNase H1, γH2AX and cPARP expression by WB. Quantification of γH2AX and cPARP intensity, data presented as mean (*n* = 2 independent experiments). In panels **a** and **c** data are presented as mean ± SEM. Scale bars = 2.5 μm. Source data are provided as a Source data file.

A widely reported cause for transcription-replication collisions and subsequent DNA damage is the presence of R-loops^1^. We therefore hypothesized that the DNA damage induced by BET bromodomain protein loss that we observed was due to the accumulation of R-loops. To test this hypothesis, we first performed immunostaining in cells using the S9.6 antibody which recognizes R-loops^31^ following treatment of cells with ARV-825. Treatment of HeLa or HCT116 cells with ARV-825 for 6 h led to increased nuclear S9.6 and γH2AX staining at the single cell level, quantified over multiple panels of cells (Fig. 3a and Supplementary Fig. 4a, respectively). The prominent cytoplasmic S9.6 staining in cells is thought to be due to the ability of the antibody to recognize mitochondrial R-loops^4^. This increase in nuclear S9.6 staining was also observed in HeLa and HCT116 cells treated with JQ1 (Supplementary Figs. 3a and 4b, respectively). These findings indicate that degradation or inhibition of BET bromodomain proteins lead to the accumulation of R-loops in cells that also show increased DNA damage signaling. To specifically test whether the observed DNA damage depends on the formation of R-loops, we next used HeLa cells stably expressing RNase H1 under the control of a doxycycline-inducible promoter^14^. These cells again displayed increased nuclear S9.6 and γH2AX immunostaining following treatment with ARV-825 or JQ1 in the absence of doxycycline-induced RNase H1 expression (−DOX panels, Fig. 3b, c, and Supplementary Fig. 3b, c, respectively). Doxycycline induction of RNase H1 expression prior to treatment of cells with ARV-825 or JQ1 led to abrogation of nuclear S9.6 and γH2AX signal (+DOX panels, Fig. 3b, c and Supplementary Fig. 3b, c, respectively). More importantly, RNase H1 expression was able to blunt the DNA damage and apoptotic response caused by degradation or inhibition of BET bromodomain proteins detected in the bulk cell population by western blot analysis (Fig. 3d and Supplementary Fig. 3d), strongly suggesting that DNA damage and apoptosis caused by ARV-825 and JQ1 is due to the accumulation of R-loops.

### Loss of BRD4 but not BRD2/3 causes R-loop-induced DNA damage

**Fig. 4:**
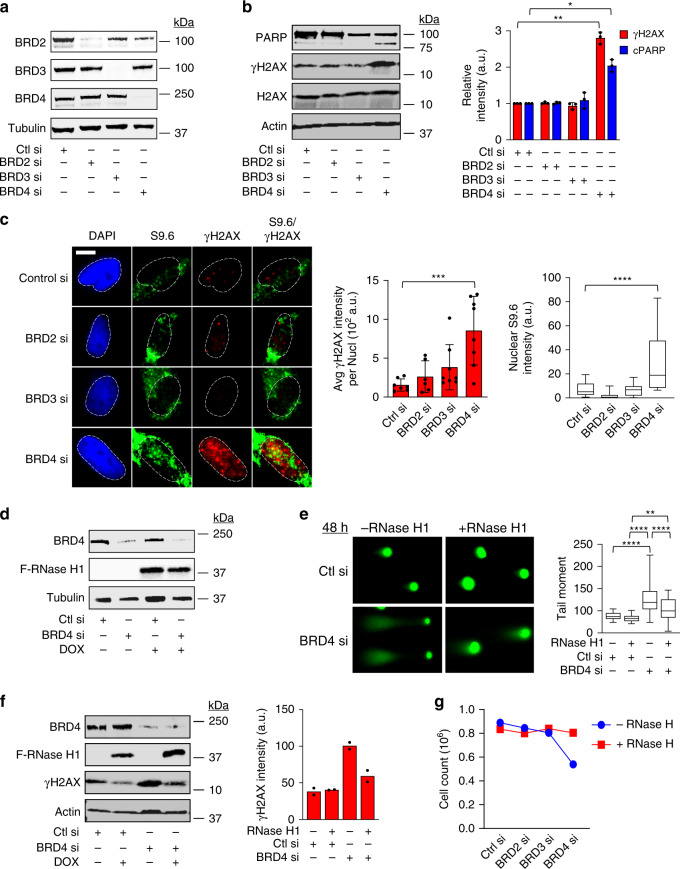
Specific loss of BRD4 leads to R-loop-induced DNA damage. **a** WB of BRD2, BRD3, and BRD4 following transfection of HeLa cells with control siRNA, or siRNAs to BRD2, BRD3, and BRD4 for 48 h. **b** Left: WB of γH2AX, total PARP, and total H2AX in cells following siRNA knockdown of BRD2, BRD3, or BRD4 in HeLa cells. Right: Quantification of γH2AX and cleaved PARP product (cPARP; lower band on PARP western blot) signal following knockdown of BRD2, BRD3, or BRD4. Data presented as mean ± SEM (*n* = 3 independent experiments). Significance assessed using ANOVA followed by Sidak’s test (γH2AX_Ctl si vs BRD4 si_ **Adjusted *P* = 0.0080; cPARP_Ctl si vs BRD4 si_ *Adjusted *P* = 0.0254). **c** Quantification and representative IF images of γH2AX and nuclear S9.6 intensities in cells following siRNA knockdown of BRD2, BRD3, or BRD4. Data presented as mean ± SEM (*n* = 3 separate experiments). Box-whisker plots are drawn as in Fig. 1b. Significance was assessed using ANOVA followed by Dunnett’s test (γH2AX_Ctl si vs BRD4 si_ ***Adjusted *P* = 0.0004; S9.6_Ctl si vs BRD4 si_ ****Adjusted *P* < 0.0001). Scale bar = 2.5 μm. **d** HeLa cells expressing doxycycline-inducible FLAG-tagged RNase H1 were treated with doxycycline for 24 h then were transfected with control or BRD4 siRNA for 48 h. Lysates were probed for BRD4 and FLAG-tagged RNase H1 by WB. Tubulin serves as loading control. **e** Fluorescence microscopy images of single cell gel electrophoresis showing tail moments following knockdown of BRD4 (BRD4 si) in the absence or presence of RNase H1 induction (*n* = 3). Box-whisker plots are drawn as in Fig. 1b. Significance was assessed using ANOVA followed by Tukey’s test ****Adjusted *P* < 0.0001, **Adjusted *P* = 0.0069. **d**, **e** Are from the same experiment. **f** Representative WB of BRD4, FLAG-tagged RNase H1, and γH2AX following knockdown of BRD4 and doxycycline-induced expression of FLAG-tagged RNase H1. Quantification of γH2AX intensity presented as mean (*n* = 2 independent experiments)*.* **g** Cell counts of RNase H-inducible HeLa cells following siRNA knockdown of BRD2, BRD3, or BRD4 for 72 h, in the presence (+RNase H, red line) or absence (−RNase H, blue line) of induced RNase H expression (*n* = 2 independent experiments). Source data are provided as a Source data file.

BET bromodomain degraders and inhibitors such as ARV-825 and JQ1 also lead to the degradation and inhibition of other bromodomain-containing proteins such as BRD2 and BRD3. To specifically address the role of BRD4 loss in the induction of DNA damage from transcription-replication collision events in cells, siRNA knockdown of either BRD2, BRD3, or BRD4 was performed in HeLa cells (Fig. 4a). As shown in Fig. 4b, only knockdown of BRD4, but not BRD2 or BRD3, significantly increased DNA damage signaling and apoptosis, as detected by western blotting for γH2AX and PARP cleavage without affecting total levels of H2AX. Similarly, only knockdown of BRD4 significantly increased nuclear S9.6 and γH2AX immunostaining in HeLa and HCT116 cells (Fig. 4c and Supplementary Fig. 4c, respectively). These data indicate that BRD4 loss of function specifically leads to R-loop-mediated DNA damage in cells. To further explore this observation, we performed BRD4 knockdown in the absence or presence of RNase H1 expression in HeLa cells (Fig. 4d) followed by single cell gel electrophoresis to quantify DSB formation and western blotting to quantify the DNA damage response in the bulk cell population. Knockdown of BRD4 in cells in the absence of RNase H1 expression led to significant increases in tail moment compared to cells that received control scramble siRNA (–RNase H1 panels, Fig. 4e). This increase in tail moment was significantly abrogated upon expression of RNase H1 (+RNase H1 panels, Fig. 4e). At the bulk cell level, western blots showed abrogation of DNA damage signaling in cells that expressed RNase H1 following transfection with BRD4 siRNA compared to control scramble siRNA (Fig. 4f), indicating that degradation of R-loops by RNase H1 was able to reverse the DNA damage caused by BRD4 loss of function. Finally, expression of RNase H1 abrogated the growth inhibition effects of BRD4 knockdown in cells (Fig. 4g), suggesting that decreasing R-loop-mediated DNA damage restores cellular proliferation. Taken together, these data suggest that loss of function of BRD4 leads to deregulated transcription and accumulation of R-loops, causing DNA damage and apoptosis.

### The BRD4 long isoform suppresses R-loop-induced DNA damage

**Fig. 5:**
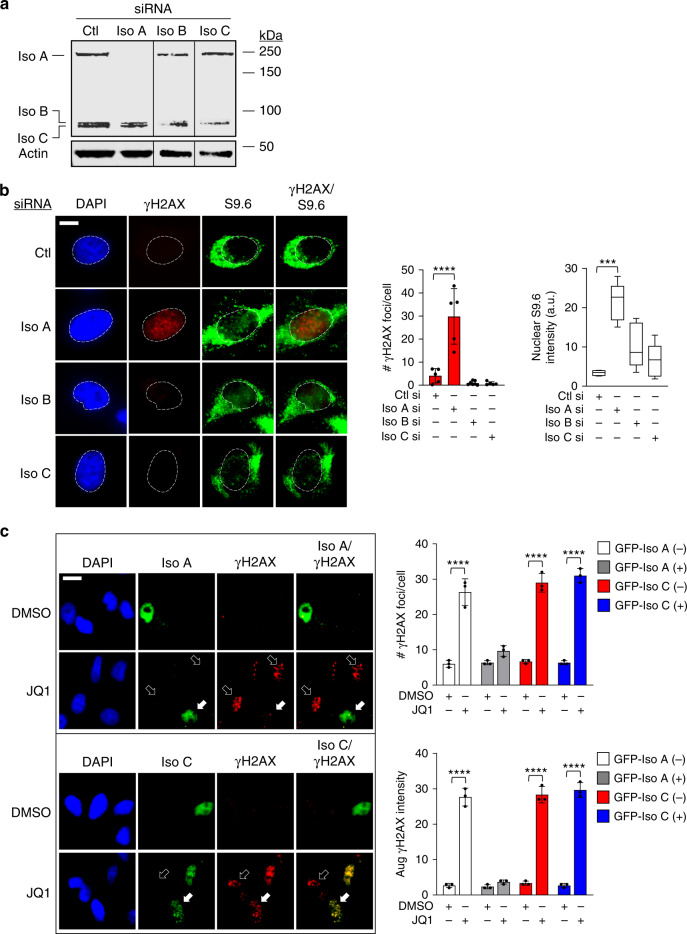
BRD4 isoform A rescues R-loop-induced DNA damage. **a** WB of endogenous levels of BRD4 isoforms A, B, and C probed with a pan-BRD4 antibody following isoform-specific siRNA knockdown. **b** Left panel: IF images of γH2AX and S9.6 fluorescence in HeLa cells following siRNA knockdown of BRD4 isoforms A, B, or C. Right panel: Quantification of γH2AX foci and S9.6 nuclear intensities (*n* = 3 separate experiments). γH2AX foci are shown as mean ± SEM. Box-whisker plots for S9.6 intensity drawn as in Fig. 1b. Significance assessed using ANOVA followed by Tukey’s test ****Adjusted *P* < 0.0001. Scale bar = 2.5 μm. **c** Left panel: IF images of γH2AX fluorescence in cells overexpressing GFP-tagged BRD4 isoforms (solid arrows) and in cells not overexpressing GFP-tagged BRD4 isoforms (open arrows) following JQ1 treatment. Right panel: quantification of γH2AX foci and integrated nuclear intensity per cell (*n* = 3 separate experiments) presented as mean ± SEM. Significance assessed using two-tailed unpaired *t* test for γH2AX foci per cell, ****P < 0.0001. Scale bar in **b**, **c** = 5 μm. Source data are provided as a Source data file.

The long (isoform A) and short (isoform C) isoforms of BRD4 have opposing functions on P-TEFb to promote or oppose gene transcription where isoform C tethers P-TEFb in its inactive form through interactions with the inhibitory molecules HEXIM1 and 7SK snRNP while isoform A liberates HEXIM1 and 7SK snRNP from P-TEFb through its C-terminal P-TEFb interaction domain^32^. Activation of P-TEFb by BRD4 isoform A leads to CTD phosphorylation and RNAPII elongation^16,33^. We specifically hypothesized that bromodomain inhibition or degradation of BRD4 isoform A results in deregulated transcription and accumulation of R-loops. To test this, we designed isoform-specific siRNAs against the three known isoforms of BRD4 in humans (isoforms A, B, and C) and confirmed their knockdown by western blotting of HeLa cell lysates using a pan-BRD4 antibody (Cell Signaling Technologies) (Fig. 5a). Immunostaining of cells demonstrating increased nuclear R-loop and γH2AX staining following knockdown of isoform A, but not isoforms B or C (Fig. 5b). This data suggests that the C-terminus of isoform A, which contains the P-TEFb interacting domain, is required for regulating R-loop-mediated DNA damage.

To further test the involvement of the specific BRD4 isoforms in effecting transcription-associated DNA damage, we performed isoform-specific rescue experiments using transient expression of either GFP-tagged isoform A (GFP-Iso A) or GFP-tagged isoform C (GFP-Iso C) in cells prior to treatment with JQ1, and assayed the ability of these constructs to reduce the DNA damaging effects of JQ1 (Isoforms A and C were examined because of their well-established functions in effecting transcription, whereas little is known for the role of isoform B in transcriptional control). Immunostaining for γH2AX revealed that cells expressing GFP-Iso A had significantly reduced γH2AX DNA damage foci and nuclear γH2AX intensity following treatment with JQ1 (Fig. 5c upper panel, solid white arrows) compared to non-expressing cells (Fig. 5c upper panel, open arrows). In contrast, cells that transiently expressed GFP-Iso C showed persistently increased numbers of γH2AX foci and nuclear γH2AX intensity following treatment with JQ1 (Fig. 5c lower panel, solid arrows) that was essentially identical to that observed in cells that did not express GFP-Iso C (Fig. 5c lower panel, open arrows). These results indicate that the normal function of the long isoform of BRD4 in transcriptional elongation prevents transcription-associated DNA damage in cells.

### BRD4 loss induces R-loops and DNA damage at a subset of genes

**Fig. 6:**
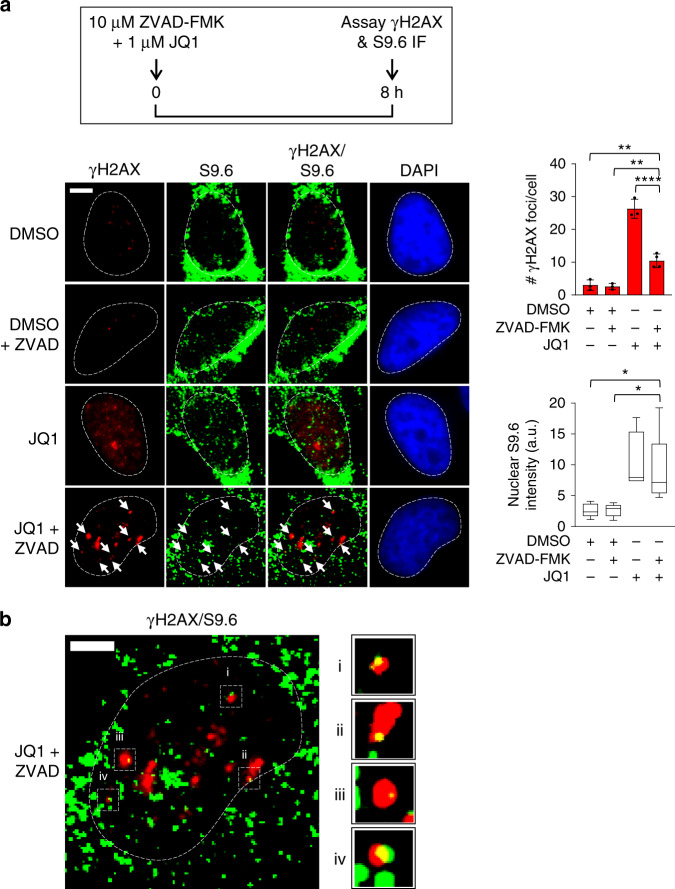
DNA damage occurs at a subset of R-loops in cells following BRD4 inhibition. **a** Upper panel: experimental design. Lower left panel: IF images of γH2AX and S9.6 fluorescence in HeLa cells treated with 10 μM ZVAK-FMK, 1 μM JQ1, or co-treated for 8 h. Lower right panel: quantification of γH2AX foci and nuclear S9.6 intensity per cell. Data presented as mean ± SEM (*n* = 3 separate experiments). Significance assessed using ANOVA followed by Tukey’s test (γH2AX_DMSO vs ZVAD-FMK + JQ1_ **Adjusted *P* = 0.0044; γH2AX_ZVAD-FMK + DMSO vs ZVAD-FMK + JQ1_ **Adjusted *P* = 0.0031; γH2AX_JQ1 vs ZVAD-FMK + JQ1_ ****Adjusted *P* < 0.0001; S9.6_DMSO vs ZVAD-FMK + JQ1_ *Adjusted *P* = 0.0444; S9.6_ZVAD-FMK + DMSO_ vs S9.6_ZVAD-FMK + JQ1_ *Adjusted *P* = 0.0838). Box-whisker plots are drawn as in Fig. 1b. Scale bar = 2.5 μm. **b** Enlarged image of a cell treated with ZVAK-FMK and JQ1 showing a subset of co-localizing nuclear γH2AX and S9.6 foci (magnified panels i–iv). Scale bars = 1 μm. Source data are provided as a Source data file.

It has recently been shown that while cells have the propensity to accumulate R-loops at multiple loci when natural processes of R-loop removal are affected (i.e. loss of RNase-H function)^9^, DNA damage appears to occur at only a fraction of these sites^34^. This, coupled with other observations that the persistence of a subset of R-loops can impair the expression of specific genes^35–37^ led us to examine whether a subset of R-loops is responsible for the DNA damage foci observed upon BRD4 inhibition in cells. To address this, cells were co-stained for γH2AX and DNA-RNA hybrids using the S9.6 monoclonal antibody. Importantly, because prolonged bromodomain inhibition results in pan-nuclear γH2AX foci on a faint diffuse γH2AX staining background, likely as a consequence of apoptosis (Fig. 1a, f), we reduced the treatment time and used a slightly higher amount of JQ1 (1 µM) in combination with the pan-caspase inhibitor ZVAD-FMK. As shown in Fig. 6a, treatment of cells with 1 µM JQ1 alone for 8 h still results in pan-nuclear γH2AX foci (Fig. 6a, JQ1), while co-treatment of cells with JQ1 and ZVAD-FMK eliminates the faint diffuse γH2AX background, and results in fewer but brighter, more well-defined γH2AX foci, without significantly affecting the S9.6 staining (Fig. 6a, JQ1 + ZVAD). This supports that a specific subset of R-loops is associated with DNA damage foci (Fig. 6b, i–iv), suggesting that not every R-loop causes DNA damage following BRD4 inhibition.

**Fig. 7:**
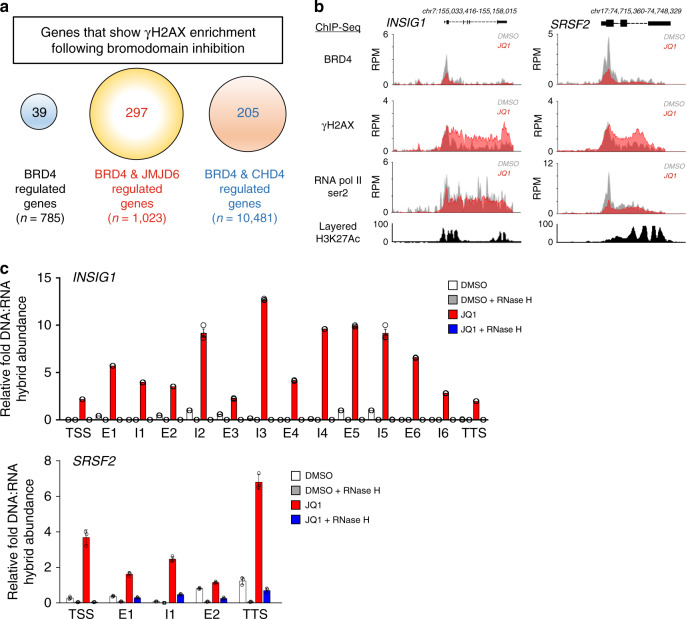
BRD4 loss induces R-loops and DNA damage at a subset of genes. **a** Diagram showing a subset of BRD4, JMJD6, and CHD4 co-regulated loci that show enrichment of γH2AX using ChIP-Seq following treatment with 500 nM JQ1 for 16 h. **b** BRD4, γH2AX, and RNAPII ser2 ChIP-Seq plots at the *INSIG1* and *SRSF2* loci following treatment with DMSO (gray) or 500 nM JQ1 for 16 h (red). In this and subsequent ChIP-Seq figures, layered H3K27Ac ChIP-Seq plots from ENCODE signify actively transcribed loci. **c** Quantification of relative abundance of DNA:RNA hybrids spanning the *INSIG1* and *SRSF2* loci following treatment with DMSO (white bars), DMSO + RNase H1 (gray bars), JQ1 (red bars), JQ1 + RNase H1 (blue bars) using DRIP-qPCR (TSS = transcription start site, E = exon, I = intron). Data presented as mean ± SEM (*n* = 3 separate experiments). Source data are provided as a Source data file.

To further identify regions of the genome that were susceptible to R-loop-associated DNA damage following bromodomain inhibition, we performed BRD4, γH2AX, and RNAPII ser2 chromatin immunoprecipitation followed by massive parallel sequencing (ChIP-Seq) in the presence of JQ1. Alignment of BRD4, γH2AX, and RNAPII ser2 ChIP-Seq peaks identified greater than 2-fold enrichment of γH2AX at 39 genes that are known to be regulated by BRD4 in humans following treatment of cells with JQ1 (Fig. 7a and Supplementary Table 1)^18^. As transcriptional regulation of genes by BRD4 often require interactions with coregulatory proteins including JMJD6 and CHD4^17^, we also looked for enrichment of γH2AX at genes that are known to be co-regulated by BRD4 and JMJD6^18^, or by BRD4 and CHD4^17^, and found enrichment at 297 genes that are known to be co-regulated by the former, and 205 genes that are known to be co-regulated by the latter, following JQ1 treatment (Fig. 7a and Supplementary Table 1). Representative ChIP-Seq profiles for *INSIG1* and *SRSF2*, along with 9 other genes belonging to all three classes, are shown in Fig. 7b and Supplementary Fig. 5, respectively. Alignment of BRD4, γH2AX, and RNAPII ser2 ChIP-Seq peaks with known H3K4Ac histone ChIP-Seq peaks from existing ENCODE datasets demonstrated that DNA damage at these BRD4 co-regulated genes correlates with mapped regions of active transcription (c.f. Supplementary Fig. 5), further supporting our hypothesis that deregulated transcriptional elongation following bromodomain inhibition leads to DNA damage. We observed a corresponding JQ1-dependent decrease in BRD4 and RNAPII ser2 at the promoters and throughout the entire gene bodies indicating a reduction in productive RNAPII elongation along the gene. Interestingly, γH2AX enrichment following JQ1 treatment not only occurred at the proximal promoter regions, where BRD4 is known to be enriched, but was similarly propagated throughout the entire gene body (Fig. 7b and Supplementary Fig. 5), consistent with DNA damage occurring throughout the gene.

We next performed DNA-RNA hybrid immunoprecipitation (DRIP) using the S9.6 antibody followed by qPCR (DRIP-qPCR) on the same subset of the genes that were identified to have increased DNA damage following treatment with JQ1 (see Fig. 7b and Supplementary Fig. 5) in order to characterize regions within these genes that were susceptible to the accumulation of R-loops. DRIP-qPCR showed increased relative abundance of R-loops throughout the transcription start site, exonic, intronic, and termination regions of *INSIG1, SRSF2*, and other BRD4, BRD4-JMJD6, and BRD4-CHD4 co-regulated genes when cells were treated with JQ1 (Fig. 7c and Supplementary Fig. 6, red bars). This finding is consistent with our prior S9.6 immunofluorescence results (Supplementary Fig. 3a, JQ1 alone). To confirm the specificity of DNA:RNA hybrid enrichment, we pretreated whole genomic extracts with RNase H1 to specifically degrade R-loops prior to performing DRIP. This resulted in loss of the qPCR signal, indicating that the signal enrichment was due to the accumulation of R-loops throughout the gene bodies at these loci following bromodomain inhibition (Supplementary Fig. 6, blue bars), again consistent with the loss of S9.6 immunofluoresence signal in cells following induced expression of RNaseH1 (Supplementary Fig. 3b). Taken together, these ChIP-Seq and DRIP-qPCR data demonstrate increased DNA-RNA hybrids in a subset of BRD4-regulated genes that show lower levels of actively elongating RNAPII following BET bromodomain inhibition, consistent with increased RNAPII stalling and/or decreased resolution of R-loops.

### BRD4 loss causes TRCs and DNA damage during S phase

**Fig. 8:**
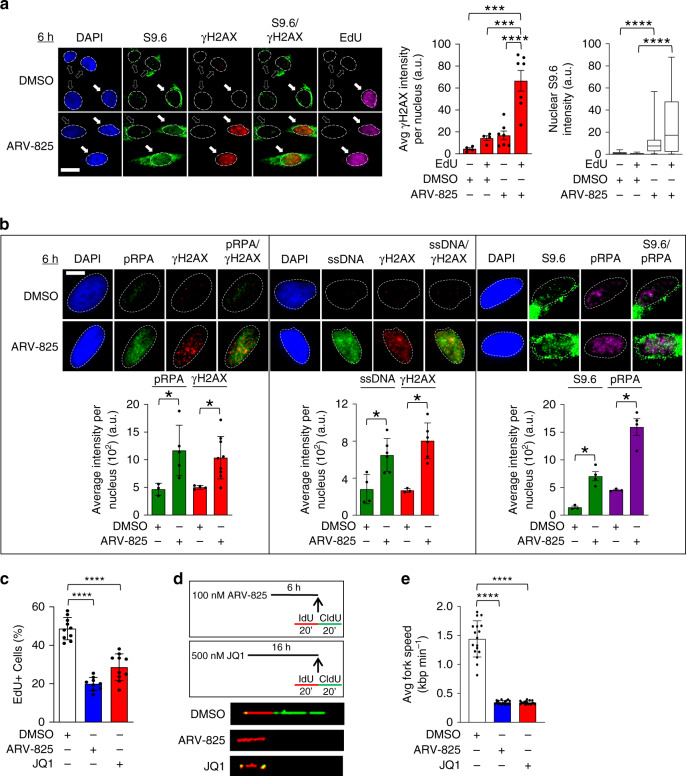
BRD4 loss causes TRCs and replication fork stalling in S-phase. **a** IF images and quantification of γH2AX and S9.6 intensities per nucleus and EdU incorporation in HeLa cells following treatment with ARV-825 (*n* = 3 separate experiments). γH2AX foci intensity data shown as mean ± SEM. Box-whisker plots for S9.6 intensity drawn as in Fig. 1b. Significance assessed using ANOVA followed by Tukey’s test (γH2AX_DMSO EdU (−) vs ARV-825 EdU (+)_ ****Adjusted *P* < 0.0001; γH2AX_DMSO EdU (+) vs ARV-825 EdU (+)_ ***Adjusted *P* = 0.0003; γH2AX_ARV-825 EdU (−) vs ARV-825 EdU (+)_ ****Adjusted *P* < 0.0001; S9.6_DMSO EdU (−) vs ARV-825 EdU (−)_ ****Adjusted *P* < 0.0001; S9.6_DMSO EdU (+) vs ARV-825 EdU (+)_ ****Adjusted *P* < 0.0001). Scale bar = 5 μm. **b** IF images and quantification of pRPA2 ser33 (pRPA), γH2AX, and native BrdU (ssDNA) immunofluorescence in cells treated with ARV-825 shown as mean ± SEM (*n* = 3 separate experiments). Significance assessed using two-tailed unpaired *t* test (*P < 0.05). Scale bar = 2.5 μm. **c** Quantification of the percentage of EdU-positive cells following treatment with DMSO (black), ARV-825 (blue), or JQ1 (red), shown as mean ± SEM (*n* = 3 separate experiments). Significance assessed using ANOVA followed by Tukey’s test (DMSO vs ARV-825 ****Adjusted *P* value < 0.0001; DMSO vs JQ1 ****Adjusted *P* value < 0.0001). **d** Experimental scheme for DNA fiber combing experiments. Fluorescent images of DNA fiber combing following treatment with either DMSO, ARV-825, or JQ1. Representative examples of normal replication, fork stalling, and stalling re-start, respectively, are shown. **e** Quantification of fork speed performed following treatment with DMSO (black), ARV-825 (blue), or JQ1 (red). Mean ± SEM from *n* = 3 separate experiments is shown. Significance assessed using ANOVA followed by Tukey’s test (DMSO vs ARV-825 ****Adjusted *P* value < 0.0001; DMSO vs JQ1 ****Adjusted *P* value < 0.0001). Source data are provided as a Source data file.

Dysregulation of the transcription machinery can directly hinder progression of replication forks, causing collision events that lead to fork collapse and activation of the ATR-dependent replication stress DNA damage response^2^. Our observations that BET bromodomain protein loss of function results in deregulated transcription with persistent R-loops, led us to hypothesize that the DNA damage we observed at specific BRD4-regulated loci was a consequence of collisions with the replication machinery. To test this hypothesis, we used two different techniques to assay whether the DNA damage that we observed was specific to S-phase cells. In one set of experiments HeLa (Fig. 8a) and HCT116 (Supplementary Fig. 7a) cells were pulse-labelled with EdU to identify S-phase cells immediately prior to treatment with the BET bromodomain degrader ARV-825 for 6 h. Cells were then immunostained for γH2AX and examined by fluorescence microscopy to compare the EdU and γH2AX signals. As shown in Fig. 8a and Supplementary Fig. 7a, cells displaying nuclear γH2AX foci after BRD4 degradation were uniformly in S-phase (EdU+), despite the fact that both EdU+ and − cells showed some nuclear S9.6 staining after ARV-825 treatment (left panel). Together, these data indicate that deregulated transcription caused by degradation of BET bromodomain proteins leads to DNA damage in S-phase as a result of collisions between the transcription and replication machinery.

### BRD4 loss causes replication stress and fork slowing

Our observation that BET bromodomain protein loss causes DNA damage specifically in S phase cells led us to further hypothesize that this reflected altered replication fork dynamics and increased replication stress. Since γH2AX is a general marker of replication stress, we assayed for more specific markers of replication stress, namely (1) the increased presence of single-stranded DNA (ssDNA) as a marker of stalled replication forks using native BrdU labelling followed by immunostaining with an anti-BrdU antibody that specifically recognizes ssDNA^38^, and (2) increased phosphorylation of RPA2 ser33 (pRPA)^39^. As shown in Figs. 8b and S7B, immunostaining of HeLa and HCT116 cells, respectively, following treatment with ARV-825 revealed increased pRPA and ssDNA staining in cells with increased γH2AX, as well as increased pRPA in cells with increased S9.6 staining, suggesting that cells with accumulation of unresolved R-loops following BET bromodomain protein loss leads to increased replication stress. Increases in markers of replication stress and DNA damage were also observed in cells treated with JQ1 (Supplementary Fig. 8a–d). Interestingly, we also noticed fewer cells in S phase following treatment with either ARV-825 or JQ1 based on our EdU labelling experiments (Fig. 8c and Supplementary Fig. 7c), suggesting that there are alterations in cell cycle dynamics or increased drop-out of cells in S phase due to DNA damage. To directly examine whether BET bromodomain protein loss of function led to altered fork dynamics, we performed DNA fiber combing assays following treatment with either ARV-825 or JQ1 using IdU and CldU labelling (Fig. 8d and Supplementary Fig. 7d). This revealed a profound decrease in replication fork speed (Figs. 8e and Supplementary Fig. 7e) in the forks that incorporated label after treatment, suggesting that BET bromodomain protein loss of function leads to TRCs resulting in increased replication stress, and decreased fork speeds.

### BRD4 inhibition leads to accumulation of cells in G_2_/M

**Fig. 9:**
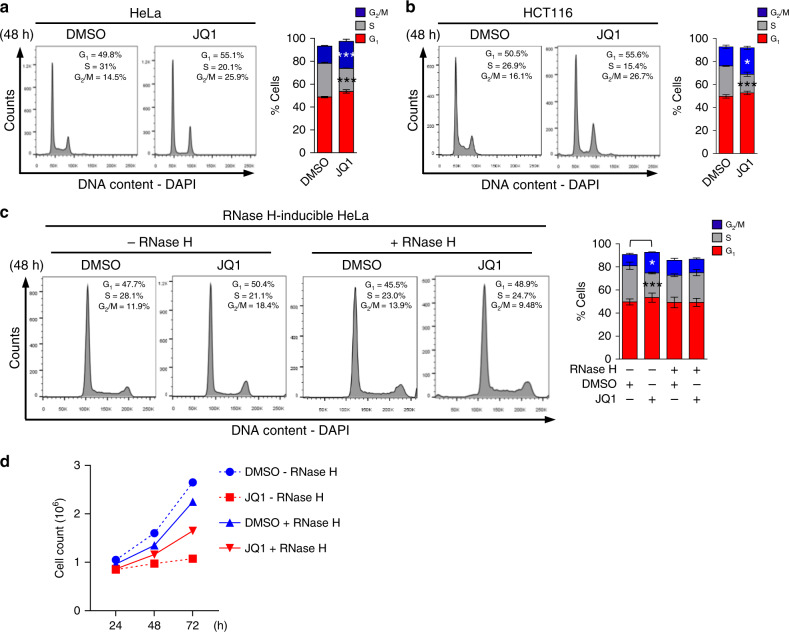
BRD4 inhibition leads to changes in the percentages of S and G2/M phase cells. Representative images and quantification of flow cytometry cell cycle plots in **a** HeLa, **b** HCT116, and **c** RNase H-inducible HeLa cells following 48 h of JQ1 treatment. Data presented as mean ± SEM (*n* = 3 separate experiments). Significance assessed using ANOVA followed by Sidak’s test in **a** (%S_DMSO vs JQ1_ ***Adjusted *P* = 0.0002; % G2/M_DMSO vs JQ1_ ***Adjusted *P* = 0.0004); **b** (%S_DMSO vs JQ1_ ***Adjusted *P* = 0.0003; % G2/M_DMSO vs JQ1_ *Adjusted *P* = 0.0188), and **c** (%S_DMSO RNase H1 negative vs JQ1 RNase H1 negative_ ***Adjusted *P* = 0.0008; %G2/M_DMSO RNase H1 negative vs JQ1 RNase H1 negative_ *Adjusted *P* = 0.0155). **d** Growth curves of RNase H-inducible HeLa cells in the absence (−RNase H) or presence (+RNase H) of DMSO or JQ1. Data presented as mean (*n* = 2 independent experiments). Please refer to Supplementary Fig. 12 for flow cytometry gating strategy. Source data are provided as a Source data file.

Our findings that BET bromodomain protein loss or inhibition causes R-loop-mediated replication stress, fork stalling, and DNA damage led us to investigate whether these effects resulted in subsequent changes in cell cycle progression/distribution and checkpoint signaling. Cell cycle analysis using flow cytometry following bromodomain inhibition with 48 h of JQ1 demonstrated a slight increase in the percentage of both G_1_ and G_2_/M cells and a corresponding decrease in the percentage of S-phase cells (Fig. 9a, b, HeLa and HCT116 cells respectively). Our results showing G_1_ accumulation with fewer S phase cells agrees with studies from Ozato’s lab that identified multiple roles for BRD4 in modulating entry into S-phase^40^, including promoting transcription of genes important for G_1_/S progression^41^ and with our prior observation that bromodomain inhibition or degradation decreases the percentage of EdU+ cells (Fig. 8c and Supplementary Fig. 7c). The increased population of cells in G_2_/M, however, was unexpected, suggesting possible aberrant signaling of the intra-S or G_2_/M checkpoint in the presence of S phase DNA damage. Since induction of RNase H1 expression abrogated the DNA damage signaling and apoptosis in cells treated with either ARV-825 or JQ1 (Fig. 3b and Supplementary Fig. 3b, respectively), we examined whether the induction of RNase H1 in JQ1-treated HeLa cells would also restore the cell cycle distribution of HeLa cells to its pre-treatment level. Indeed, as shown in Fig. 9c, the modest increase in G_1_ and G_2_/M populations and reduction in the S-phase population caused by JQ1 treatment was abrogated by RNase H1 expression, which also partially rescued the JQ1-induced block in cell proliferation (Fig. 9d). These results suggest that removal of transcriptional roadblocks caused by the accumulation of R-loops following BET bromodomain protein loss leads to resolution of TRCs and restoration of normal cell cycle kinetics.

### BRD4 loss impairs the Chk1 S-phase DNA damage checkpoint

**Fig. 10:**
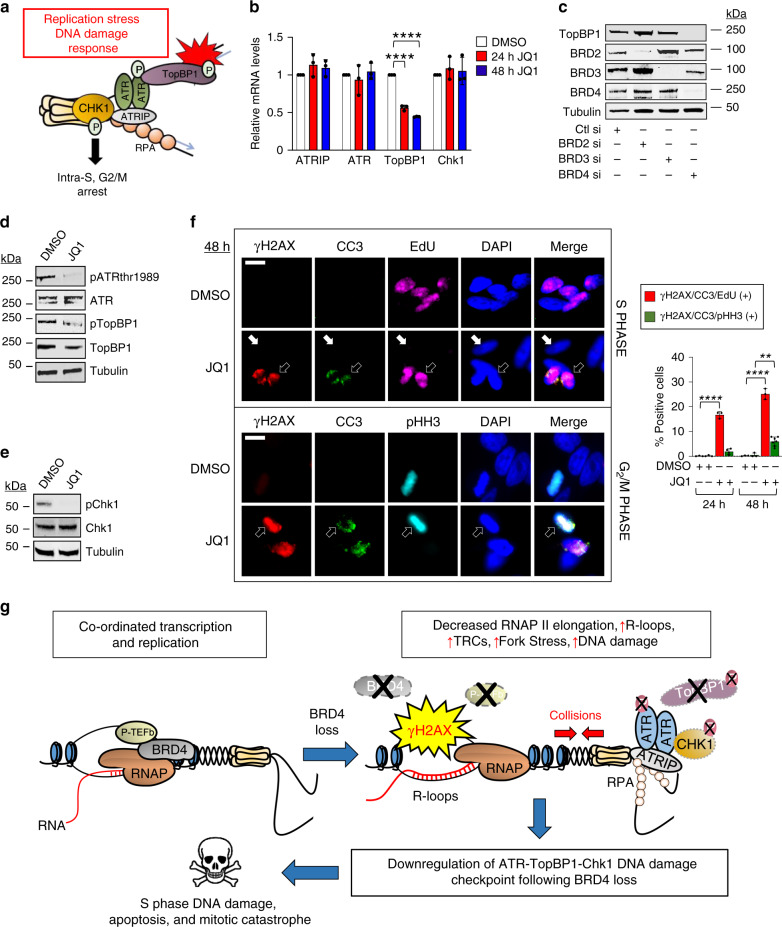
BRD4 loss impairs the replication stress DNA damage checkpoint. **a** Overview of the ATR-TopBP1-Chk1 replication stress DNA damage checkpoint. TopBP1 is required for Chk1 activation and recruitment^31,82^. **b** Quantification of RT-qPCR of ATRIP, ATR, TopBP1, and Chk1 mRNA levels following treatment with 500 nM JQ1 for 24 and 48 h in HeLa cells. Data presented as mean ± SEM (*n* = 3 independent experiments). Significance assessed using ANOVA followed by Tukey’s test (24 h DMSO_TopBP1_ vs JQ1_TopBP1_ ****Adjusted *P* < 0.0001; 48 h DMSO_TopBP1_ vs JQ1_TopBP1_ ****Adjusted *P* < 0.0001). **c** WB of TopBP1, BRD2, BRD3, BRD4, and tubulin following transfection with control siRNA or siRNA to BRD2, BRD3, or BRD4. **d**, **e** WB of pATRthr1989, ATR, pTopBP1ser1138, TopBP1, and tubulin (**d**), and pChk1ser345, Chk1, and tubulin (**e**), following 24 h of treatment with JQ1. **f** IF images and quantification as in Fig. 1a of cells that stain triply positive for γH2AX, CC3 and EdU (upper panel) or γH2AX, CC3, and pHH3 (lower panel) following JQ1 treatment. Data presented as mean ± SEM (*n* = 3 independent experiments). Significance assessed using two-tailed unpaired *t* test (24 h DMSO_γH2AX/CC3/EdU (+)_ vs JQ1_γH2AX/CC3/EdU (+)_ ****P < 0.0001; 48 h DMSO_γH2AX/CC3/EdU (+)_ vs JQ1_γH2AX/CC3/EdU (+)_ ****P < 0.0001; 48 h DMSO_γH2AX/CC3/pHH3 (+)_ vs JQ1_γH2AX/CC3/pHH3 (+)_ **P = 0.0012). Scale bar = 5 μm. **g** Global model of how BRD4 loss results in DNA damage and cell death as a consequence of loss of productive RNAPII elongation, R-loop accumulation, transcription-replication conflicts, and failure to activate the ATR-TopBP1-Chk1 axis. Source data are provided as a Source data file.

Increased replication fork stalling and DNA damage in S phase typically induces the ATR-dependent replication stress DNA damage response, activating cell cycle checkpoints that facilitate DNA repair and restore replication fork integrity, thereby avoiding genomic instability^39,42^. The replication stress DNA damage checkpoint involves recruitment of RPA, ATRIP, and ATR to stretches of exposed ssDNA at stalled forks. The DNA damage response mediator protein TopBP1 is then recruited to this complex and, following a series of phosphorylation events ultimately leads to the recruitment, phosphorylation, and activation of Chk1 with induction of the intra-S and/or G_2_/M checkpoints (Fig. 10a)^42,43^. The observation that BET bromodomain inhibition caused cells to accumulate in G_2_/M led us to hypothesize that BET bromodomain proteins may regulate the ATR-TopBP1-Chk1 pathway, possibly through transcriptional regulation of these key players. To test this, we performed qPCR to assess mRNA levels of ATRIP, ATR, TopBP1, and Chk1 in cells treated with JQ1 at 24 and 48 h and observed progressive decreases in the mRNA levels of TopBP1 but not ATR, ATRIP, or Chk1 (Fig. 10b). Next we performed selective knockdowns of BRD2, BRD3, or BRD4 and measured the protein levels of TopBP1 72 h following knockdown. As shown in Figs. 10c and S9A, knock-down of BRD4, but not BRD2 or BRD3, resulted in reduced levels of TopBP1 protein, suggesting that BRD4 was the predominant BET bromodomain protein that regulated TopBP1 expression. We then cross-referenced this observation with our ChIP-Seq data and observed a BRD4 peak at the promoter region of TopBP1 which decreased following treatment with JQ1, indicating that BRD4 plays a direct role in regulating transcription of *TOPBP1* (Supplementary Fig. 9b). Phosphorylation of TopBP1 at serine residue 1138 (pTopBP1) in its ATR activation domain in the presence of replication stress and DNA damage is thought to enable further autophosphorylation of ATR at threonine residue 1989 (pATRthr1989) for induction of Chk1 (pChk1)^43,44^. To assess the signaling properties of this network of proteins following bromodomain inhibition, whole-cell lysates were examined by western blotting, revealing decrease levels of total TopBP1, pTopBP1, and pATRthr1989 with no significant changes in the levels of total ATR following 24 h of JQ1 treatment (Fig. 10d and Supplementary Fig. 9c). These findings suggest that downregulation of TopBP1 expression levels following bromodomain inhibition leads to loss of amplification of the replication stress DNA damage response despite increased DNA damage in S phase cells. This results in a decrease in Chk1 activation (Fig. 10e and Supplementary Fig. 9d), which may explain the absence of S phase arrest and increase in the population of cells in G_2_/M that we observed upon JQ1 treatment (Fig. 9a–c). We further hypothesized that the decreased percentage of cells in S phase could also result from increased apoptosis of damaged cells in S phase, leading to cell drop-out with prolonged treatment with JQ1 as well as slippage of late S phase cells into mitosis due to downregulation of the ATR-TopBP1-Chk1 DNA damage checkpoint, leading to mitotic catastrophe. To test this, we performed IF to quantify populations of cells that stained triply positive for γH2AX, cleaved caspase 3 (CC3), and EdU to identify DNA damaged apoptotic cells in S phase, as well as cells that were triple positive for γH2AX, CC3, and pHH3 in cells with condensed chromatin as a marker of mitotic catastrophe. As shown in the upper left panel of Fig. 10f, cells that were EdU positive had increased γH2AX and CC3 following treatment with JQ1 (open arrows), while cells that were not EdU positive demonstrated neither DNA damage nor apoptosis (solid arrows). Similarly, JQ1 treatment led to increased numbers of cells that stained triple positive for γH2AX, CC3, and pHH3, consistent with a subset of cells dying by mitotic catastrophe (Fig. 10f and Supplementary Fig. 9e, left lower panel, open arrows). These results, quantified in the right panel, support a model in which BRD4 dysfunction results in loss of productive RNAPII elongation, R-loop accumulation, transcription-replication conflicts, and a paradoxical failure to activate the ATR-TopBP1-Chk1 axis, causing death in S-phase and slippage of damaged cells into mitosis, where they die by mitotic catastrophe (Fig. 10g). These findings identify new roles for BRD4 in resolution of transcription-replication conflicts and regulation of the replication stress DNA damage response in cells.

## Discussion

BRD4 is a well-established transcriptional co-activator that binds to open acetylated chromatin through its bromodomains, and, in the case of the long isoform, recruits the core transcription elongation regulator P-TEFb^16^ to promoters to phosphorylate the C-terminal domain of the large subunit of RNAPII^27,34^. In addition, BRD4 is required for subsequent progression of RNAPII through hyperacetylated nucleosomes during elongation through interactions of its bromodomains with acetylated histones in order to prevent transcriptional stalling^25,26^. Among its known targets, BRD4 regulates genes involved in cell cycle progression and lineage specification^40^, and is particularly important for the transcription of super-enhancer associated genes such as *MYC*^19,20^. Indeed, inhibition of BRD4 results in notable anti-cancer effects in a variety of *MYC*-driven cancers, including leukemias, lymphomas, and multiple myeloma^20,22,45^. However, targeted disruption of BRD4 function also results in tumor cell killing in a variety of solid tumors where *MYC* does not appear to be a major cancer driver, including certain lung cancers, prostate cancers, and glioblastoma^46–50^. The detailed molecular mechanisms responsible for the killing of MYC-independent tumors by BRD4 inhibition are incompletely understood but may involve various aspects of DNA damage and repair^21,51–53^. Here we report that inhibition or loss of BRD4 leads to the accumulation of R-loops, causing collision events between the transcription and replication machinery during S phase. In addition, transcriptional downregulation of TopBP1 by BRD4 results in suppression of ATR-Chk1 cell cycle checkpoint signaling, resulting in apoptosis in S phase and mitotic catastrophe.

It is well known that the lack of spatiotemporal separation between transcription and replication along the same DNA template in mammalian cells can lead to collision events and genome instability, which depends on both the orientation of the collisions (head-on vs co-directional) and the nature of the transcriptional blockade^1^. These collisions occur due to the fact that transcription of nascent RNA results in the formation of R-loops through the activity of RNA polymerase on double-stranded DNA, whereas DNA replication occurs on separated single-stranded DNA at the replication fork. As such, the replication fork cannot progress past an elongating RNA polymerase, leading to collision events^2^. Our data showing that degradation (Fig. 8a and Supplementary Fig. 7a) or inhibition (Fig. 10f) of BET bromodomain proteins in several oncogenic cell lines leads to increased DNA damage specifically during S-phase, supports our model that a normal function of BRD4 in these cells is to enhance transcription of RNAPII-bound genes, thus ensuring proper spatio-temporal co-ordination between the transcription and replication machinery. This is further supported by our observation that DNA damage and apoptosis due to BET bromodomain protein loss is abrogated by either blocking initiation of transcription using triptolide (Fig. 2d), or by inducing RNase H1 to degrade R-loops (Fig. 3b and Supplementary Fig. 3b), indicating that removing transcriptional roadblocks caused by BRD4 loss effectively eliminates collisions with the replication machinery. Our findings are supported by two recent studies also reporting the ability of bromodomain inhibition to increase DNA damage in several oncogenic cell lines. A systematic bromodomain protein screen by Kyle Miller’s group identified BRD2 and BRD4 as regulators of R-loop-associated DNA damage in U2OS cells. In their study, they also showed that inhibition of transcription initiation with triptolide or overexpression of RNase H1 abrogated the effects of DNA damage following bromodomain inhibition^54^. Similarly, an elegant study by Scott Floyd’s group very nicely delineated the need for RNAPII occupancy on chromatin to elicit DNA damage following BRD4 loss by again demonstrating that pretreatment of cells with triptolide prevented BET degrader-induced DNA damage by degrading RNAPII on chromatin, while 5,6-Dichlorobenzimidazole 1-β-d-ribofuranoside (DRB), which inhibits transcription by stalling of RNAPII, did not^55^. Both of these groups then used a kinase-dead RNase H1 that binds but does not degrade R-loops and performed R-ChIP qPCR, identifying genes that had increased R-loops following BET protein loss. Taken together, these studies echo our hypothesis that bromodomain proteins are involved in R-loop-mediated DNA damage in oncogenic cells.

That interference with BRD4 function leads to R-loop accumulation and DNA damage throughout the body of affected genes is consistent with multiple roles of BRD4 in transcriptional elongation. BRD4 performs at least two distinct functions during mRNA transcription: it facilitates the transition from transcriptional initiation to elongation (i.e. promoter proximal pause release), and it also prevents RNAPII stalling throughout elongation through its continual interaction with acetylated histones. BRD4 is known to release the PTEF-b complex from the inhibitory factors HEXIM1/2 and 7SK snRNA, allowing for its transition to its active form with subsequent phosphorylation of RNAPII at ser2, as required for efficient transcriptional elongation^25,26,33,56–58^. Our data is also consistent with findings from Zhang et al, who showed that JQ1 treatment resulted in reduced RNAPII ser2 throughout the bodies of BRD4-regulated genes in CD4+ T-cells^26^, and work by Liu et al, who showed decreased RNAPII travelling ratios (suggestive of RNAPII pausing) across RNAPII-bound genes, and decreased RNAPII ser2 across BRD4 regulated genes following BRD4 loss of function^59^. Importantly, however, work by Keiko Ozato’s group has now shown that BRD4 is involved not only in facilitating pause-release at proximal promoters and the transition to transcriptional elongation at transcription start sites and enhancers, but also assists RNAPII progression throughout the gene bodies through interactions with acetylated histones via its bromodomains, independent of PTEF-b^25^. Furthermore, this process of BRD4-mediated passage of RNAPII through the body of transcriptionally elongating genes was directly antagonized by bromodomain inhibitors. Thus, BRD4 functions as an elongation factor that enhances RNA polymerase processivity all along the gene body, independently of its role at promoters^25^. Our experimental findings showing loss of productive RNAPII elongation, concomitant with increased RNA-loops and DNA damage throughout the gene bodies of a subset of BRD4-regulated genes following JQ1 treatment (Fig. 7b, c, Supplementary Figs. 5 and 6), is fully consistent with these models of BRD4 function from the literature.

RNAPII stalling has been associated with the accumulation of R-loops^60^, and that the inability to resolve or remove R-loops causes DNA damage and genomic instability^3,61,62^. The study by Miller’s group identified a putative interaction between the C-terminus region of BRD2 and Topoisomerase 1 (TOP1), which relaxes negative supercoiled DNA and is known to reduce transcription-associated R-loops^63–65^. They demonstrated the addition of recombinant BRD2 stimulated TOP1 relaxation activity in vitro while BRD3 and BRD4 did not, suggesting that BRD2 loss or JQ1 treatment could explain the increased R-loop-associated DNA damage^54^. Furthermore, they also observed that reduction of BRD4 resulted in R-loop-associated DNA damage, and BRD4 has been shown to regulate TOP1 activity through the phosphorylation of the C-terminal of RNAPII^66^. Taken together, these data and ours further support a role for BRD4 in preventing the accumulation of R-loops at RNAPII-transcribed genes.

Downregulation of R-loop resolving proteins following BRD4 inhibition could also potentially contribute to the persistence of R-loops and DNA damage at late times. Cristini et al performed mass spectrometry on S9.6 immunoprecipitates in HeLa cells and identified key proteins in the R-loop interactome that are involved in R-loop suppression and transcription termination^67^. That study identified previously known proteins involved in R-loop biology such senataxin (SETX)^61,68^ and the serine/arginine-rich splicing factor 1 (SRSF1)^60,69^, and also identified novel functions for the helicase DHX9 in R-loop suppression and transcription termination. Transcription of *DHX9* is known to be co-regulated by BRD4 and JMJD6^18^. Our ChIP-Seq analysis identified loss of BRD4 and RNAPII ser2 with increased γH2AX at *DHX9* (Supplementary Fig. 10a). This corresponded to gradual suppression of DHX9 protein levels over a 24 to 48 h time course of JQ1 treatment (Supplementary Fig. 10b). Transcription of *SETX* and *SRSF1* has not been previously reported to be regulated by BRD4, however, analysis of the *SETX* and *SRSF1* loci, showed BRD4 and RNAPII ser2 peaks at their promoters under basal DMSO conditions which were reduced following treatment with 16 hours of JQ1 (Supplementary Fig. 10a), along with correspondingly decreased SETX and SRSF1 protein levels (Supplementary Fig. 10b). Edwards and colleagues did not report changes in SETX or SRSF1 protein levels within the 6 hours of treatment with the BET protein degrader dBET6 where they saw increased DNA damage, similar to the timeframe where we also saw increased R-loop accumulation and DNA damage with ARV-825^55^, suggesting that the inability to degrade R-loops by these R-loop processing proteins is unlikely to be the primary mechanism responsible for early R-loop-associated DNA damage following BRD4 loss, but could conceivably contribute to the inability of cells to resolve R-loops particularly at later times, leading to persistent TRCs and DNA damage.

Importantly, the ability of bromodomain inhibition to cause DNA damage and replication stress varies across cancer cell lines. Our previous work characterizing the effects of bromodomain inhibition on ionizing radiation-induced DNA damage in U2OS cells showed that exposure of U2OS cells to JQ1 alone did not elicit a DNA damage response^21^, in contrast to what we observed here in HeLa, HCT116, and previously in U87MG and GL261 cells^50^, and what others have observed in human myeloid leukemic cell lines^70^. We further characterized this absence of DNA damage signaling (Supplementary Fig. 11a) and DSB formation (Supplementary Fig. 11b) in U2OS cells following prolonged treatment with 1 μM JQ1, suggesting that these cells are inherently insensitive to bromodomain inhibitor-induced DNA damage. This finding was recapitulated in a study by Zhang et al in which they failed to detect DNA damage or cell cycle perturbation in U2OS cells treated with another bromodomain inhibitor, AZD5153, although they did observe elevated γH2AX levels in OVCAR3 and OVCAR4 cells treated with the same drug for 24 h^71^. Furthermore, while bromodomain inhibition alone in U2OS had little effect, it synergized with replication stress-inducing agents including HU-treatment and with ATR inhibition to cause increased γH2AX signaling. Taken together, their findings and ours suggest that BRD4 plays an important general role in regulating replication stress responses, and that the effect of BRD4 inhibitors alone on inducing DNA damage in cancer cells may depend on the basal level of oncogene-induced replication stress. Additional experiments will be required to confirm or refute this hypothesis.

The ability of JQ1 to downregulate transcription of *TOPBP1* was recently reported by Karakashev and colleagues, who reported downregulation of *TOPBP1* mRNA as early as 30 min following JQ1 treatment^72^. Similarly, downregulation of *TOPBP1* was also demonstrated in BRCA1-proficient OVCAR3 ovarian cancer cells, however, unlike Zhang et al.^71^ and our model, in that study, JQ1 alone did not elicit DNA damage nor PARP cleavage products in OVCAR3 cells, but synergized with the PARP inhibitor Olaparib to increase DNA damage and tumor cell killing in vivo^72^. Finally, a third study by Bowry et al recently reported effects of bromodomain inhibition in U2OS cells on upregulating the transcription of histone and non-polyadenylated non-coding RNA genes, resulting in TRCs and replication fork slowing, leading to recruitment of Rad 51^57^. Interestingly, these authors reported recovery of fork speeds after prolonged incubation of JQ1 (24–72 h) suggesting adaptation of U2OS cells to the effects of JQ1 on replication fork dynamics. We did not observe this effect in HeLa and HCT116 cell lines, which showed persistently stalled forks following JQ1 treatment (Fig. 8d, e and Supplementary Fig. 7d, e), and might contribute to the conflicting effects of BRD4 inhibition alone in different cell types. Those authors further demonstrated rescue of BRD4 siRNA-induced fork slowing with ectopic expression of isoform A, but not isoform C of BRD4, but failed to observe increases in DNA damage or RPA foci following treatment with JQ1. These studies suggest that U2OS cells may have an inherently lower level of endogenous replication stress which protects them from the DNA damaging effects of bromodomain inhibition. Nonetheless, their results echo our findings that ectopic expression of isoform A but not C rescues the DNA damage response induced by JQ1 in HeLa cells by resolution of TRCs (Fig. 5c). Finally, it is interesting that we consistently observe phosphorylation of RPA2 ser33 despite our observed paradoxic decrease in the ATR-TopBP1-Chk1 pathway following BRD4 loss (Fig. 10d, e and Supplementary Fig. 9c, d). There is now strong evidence suggesting that phosphorylation of RPA ser33 occurs through an ETAA1-ATR interaction which is independent of the activation of ATR by TopBP1^73,74^. These studies show that phosphorylation of RPA is primarily dependent on ETAA1 and only modestly affected by TopBP1 knockdown. We can thus infer that there is an appropriate replication stress response to the increases in TRCs however cells cannot appropriately trigger the replication stress DNA damage checkpoint due to downregulation of TopBP1 following BRD4 loss. With recent studies showing synthetic lethality of bromodomain inhibitors in combination with PARP inhibitors for the treatment of different tumor models^51,75^, understanding the role of BRD4 in regulating the replication stress DNA damage response may allow for the development of additional rational combination therapies using bromodomain inhibitors to improve treatment outcomes for a variety of cancers.

## Methods

### Cell culture

HeLa (ATCC CCL-2) and HCT116 (ATCC CCL-247) cells were purchased directly from the American Type Culture Collection and used without further authentication. RNase H1-inducible HeLa cells (gift from Cimprich Lab, Stanford University) were grown in DMEM containing 10% FBS. RNase H1-inducible HeLa cells were grown in DMEM containing 10% FBS and 1 mg ml^−1^ puromycin. Doxycycline (0.5 mg ml^−1^) was used to induce expression of RNase H1. All cell lines were tested for mycoplasma contamination.

### Antibodies and stains

Monoclonal antibodies against γH2AX were purchased from Millipore Sigma (Catalog No. 05-636) and Cell Signaling Technologies (Catalog No. 9718S); H2AX (Cell Signaling Technologies, Catalog No. 7631); actin (Sigma, Catalog No. A5441); cleaved PARP (BD Pharmigen, Catalog No. 552596); PARP (Cell Signaling Technologies, Catalog No. 9532S); cMyc (Cell Signaling Technologies, Catalog No. 5605S); RNA Pol II (Millipore Sigma, Catalog No. 05-623), BRD4 (Cell Signaling Technologies, Catalog No. 13440S); BRD2 (Cell Signaling Technologies, Catalog No. 5848S); BRD3 (Abcam, Catalog No. ab50818); tubulin (Sigma, Catalog No. T5168); FLAG peptide (Cell Signaling Technologies, Catalog No. 14793S); S9.6 (Kerafast, Catalog No. ENH001); senataxin (Abcam, Catalog No. ab220827); DHX9 (ThermoFisher, Catalog No. PA5-19542); SRSF1 (Thermofisher, Catalog No. 32-4600); ATR (Cell Signaling Technology, Catalog No. 2790S); phosphorylated ATR threonine 1989 (Genetex, Catalog No. GTX128145); phosphorylated RPA32 serine 33 (Bethyl, Catalog No. A300-246A); TopBP1 (Bethyl, Catalog No. A300-111A); phosphorylated TopBP1 serine 1138 (Raybiotech, Catalog No. 102-15561); phosphorylated Chk1 serine 345 (Cell Signaling Technology, Catalog No. 2348S); Chk1 (Cell Signaling Technology, Catalog No. 2360S). Pan-BRD4 polyclonal antibody was a gift from Cell Signaling Technologies (Clone PP12). DAPI DNA stain was from ThermoFisher (Catalog No. 62248). SYBR Gold nucleic acid stain was from ThermoFisher (Catalog No. S11494). Fluorescent antibodies were from ThermoFisher: goat anti-rabbit Alexafluor 488 (Catalog No. A-11008), Alexafluor 555 (Catalog No. A-21428), Alexafluor 647 (Catalog No. A-21245); and goat anti-mouse Alexafluor 488 (Catalog No. A-11001), 555 (Catalog No. A21424), and 647 (Catalog No. A-21235). Western blotting secondary antibodies were purchased from Licor: IRDye 800CW goat anti-rabbit IgG (Catalog No. 926-32211) and IRDye 680RD goat anti-mouse IgG (Catalog No. 925-68180). EdU (Catalog No. C10337) and EU (Catalog No. C10329) labeling were performed using Click-IT chemistry kits from Invitrogen. All primary antibodies were used at a dilution of 1:1000. All Alexafluor secondary antibodies were used at a dilution of 1:1000. All Licor antibodies were used at a dilution of 1:20,000.

### Small molecule inhibitors

BET bromodomain inhibitor JQ1 was a gift from J. Bradner. These were used at 100 nM, 500 nM, and 1 μM, respectively. BET bromodomain PROTAC ARV-825 (MedChem Express) was used at 100 nM. Triptolide (Sigma) was used at 100 nM and 1 μM. All small molecule inhibitors were dissolved in DMSO (Sigma). ZVAD-FMK pan-caspase inhibitor (Sigma) was used at 10 μM.

### Short interference RNA loss of function studies

Silencer Select® validated siRNAs against BRD2, BRD3, BRD4, and Negative Control siRNA were purchased from Life Technologies. Custom select BRD4 isoform A siRNA (Sense 5′-CCAUUGACAUGAAUUUCCAtt-3′, Antisense 5′-UGGAAAUUCAUGUCAAUGGta); BRD4 isoform B siRNA (Sense 5′-AGAGUGUGCUCGUUGCUGUtt-3′, Antisense 5′-ACAGCAACGAGCACACUCUgg-3′); and BRD4 isoform C siRNA (Sense 5′-GCUCCUCUGACAGCGAACAtt-3′, Antisense 5′-UCUUCGCUGUCAGAGGACtg-3′) were purchased from Life Technologies. Knockdown experiments were performed as per manufacturer’s instructions using Lipofectamine RNAi Max (Invitrogen).

### Constructs and transient transfections

Full length constructs of BRD4 isoforms A (accession number NM_058243) and C (accession number NM_014299.2) were cloned into pEGFP-C1 (Clontech) by PCR. Transient transfections were performed using Xtremegene 9 (Roche) according to manufacturer’s specifications 12 h prior to treatment of HeLa cells with DMSO or 500 nM JQ1 for 16 h.

### Chromatin immunoprecipitation and library preparation

HeLa cells were treated with DMSO or 500 nM JQ1 for 16 h, fixed with 11% formaldehyde, harvested, and lysed in lysis buffer (50 mM HEPES-KOH, pH 7.5, 140 mM NaCl, 1 mM EDTA, 10% glycerol, 0.5% NP-40, 0.25% Triton X-100), spun at 10,000 rpm for 10 min at 4 °C, and supernatant discarded. Protease inhibitors (Roche) and phosphatase inhibitors (Roche) were added to all buffers. Cells were washed three times in lysis buffer and prepared for sonication using a Bioruptor (30 s on, 30 s off, 6 rounds of 10 min) at 4 °C. Protein G Dynabeads (Thermofisher) was prepared according to manufacturer’s specifications and either BRD4 antibody (Cell Signaling Technologies, Catalog No. 13440S), γH2AX (Millipore Sigma, Catalog No. 05-636), or RNAPII CTD repeat YSPTSPS phospho S2 (Abcam, Catalog No. ab5095) conjugated to the magnetic beads. All antibodies were validated ChIP grade as per the manufacturer. Equal amounts of sheared chromatin were subjected to chromatin immunoprecipitations overnight at 4 °C. Chromatin-bound beads were washed three times for 10 min at 4 °C in wash buffer (20 mM Tris-HCl, pH 8.0, 150 mM NaCl, 2 mM EDTA, 0.1% SDS, and 1% Triton X-100), then once in washer buffer with the addition of 500 mM NaCl, followed by one wash in lithium chloride wash buffer (10 mM Tris-HCl, pH 8.0, 250 mM LiCl, 1 mM EDTA, 1% NP-40), and a final wash in TE buffer with 50 mM NaCl. Cross-linked chromatin immunoprecipitates were eluted in elution buffer (50 mM Tris-HCl, pH 8.0, 10 mM EDTA, 1% SDS) at 65 °C for 15 min. Reverse crosslinking of the eluate was performed at 65^o^C overnight, followed by the addition of RNase A treatment (Thermofisher, 0.2 mg/mL final concentration) at 37 °C for 3 h, and finally Proteinase K treatment (Thermofisher, 0.2 mg/mL final concentration) at 55 °C for 2 h. Purified DNA was isolated using phenol:chloroform extraction. Library preparation for Illumina NextSeq was performed using the NEBNext Ultra II DNA library prep kit (NEB) as per manufacturer’s protocol. The quality of the library prep was assayed using a BioAnalyzer (Agilent Technologies) prior to loading of samples onto an Illumina NextSeq to perform sequencing of 75 nucleotide paired-end reads at 40 million read depth.

### ChIP-Seq read mapping

Paired-end ChIP-Seq sequencing data were mapped against the human genome hg19 assembly using the Burroughs-Wheeler Aligner BWA-MEM v. 0.7.12-r1039 with flag –t 4 and otherwise default parameters [**bio-bwa.sourceforge.net**]^76^. The resulting bam files were sorted and indexed using samtools v. 1.5 [http://www.htslib.org and **LI2009**], and duplicates were marked using Picard v. 2.9.0-1-gf5b9f50-SNAPSHOT (https://broadinstitute.github.io/picard/) MarkDuplicates with flags MAX_SEQUENCES_FOR_DISK_READ_ENDS_MAP = 50000 MAX_FILE_HANDLES_FOR_READ_ENDS_MAP = 8000 SORTING_COLLECTION_SIZE_RATIO = 0.25 REMOVE_DUPLICATES = false ASSUME_SORTED = false DUPLICATE_SCORING_STRATEGY = SUM_OF_BASE_QUALITIES PROGRAM_RECORD_ID = MarkDuplicates PROGRAM_GROUP_NAME = MarkDuplicates OPTICAL_DUPLICATE_PIXEL_DISTANCE = 100 VERBOSITY = INFO QUIET = false VALIDATION_STRINGENCY = SILENT COMPRESSION_LEVEL = 5 MAX_RECORDS_IN_RAM = 500000 CREATE_INDEX = false REMOVE_SEQUENCING_DUPLICATES = false TAGGING_POLICY = DontTag READ_NAME_REGEX = < optimized capture of last three ‘:’ separated fields as numeric values > CREATE_MD5_FILE = false GA4GH_CLIENT_SECRETS = client_secrets.json. Bam files with duplicate reads marked were sorted and indexed again prior to being processed for downstream analyses.

### ChIP-Seq peak calling and visualization

ChIP-Seq peaks were called using MACS2 v. 2.1.1.20160309 callpeak function^77^ with parameters –g hs –call-summits–p 1e-3 –nomodel –B with –ext matching the calculated insert size of each library, and using above-mentioned bam files from whole-cell extract and the chromatin-associated protein of interest as control and treatment, respectively. The resulting “narrowPeak” files were used for peak identification. In addition, wig files were prepared for each library using igvtool’s count function^78^, with –e matching the calculated insert size of the library and -w 25, which were converted to BigWig using UCSC’s wigToBigWig tool with default parameters and hg19’s chromosome sizes as an input.

### Gene-specific ChIP read density plots

Coordinates of candidate genes were retrieved and expanded to a suitable distance 5′ and 3′ of the gene boundaries. Read densities were calculated by extending each read to 200 bps and tallying read counts over 25-bp bins tiling the regions of interest^79^. Genomic bins containing statistically significant ChIP-Seq enrichment were identified by comparision to a Poissonian background model, using a *p*-value threshold of 10^−9^. Final read counts were normalized by each library’s sequencing depth.

### Gene distributions of ChIP-Seq reads

Genomic features were retrieved from the ENSEMBL GRCh37 v. 75 annotation of the hg19 genome assembly and reads were sequentially apportioned to transcription start sites (TSS) regions (defined as 50 bp upstream to 300 bp downstream of annotated TSSs), 5′ UTRs, transcription termination site regions (TTS, defined as 50 bp upstream and 200 bp downstream of annotated 3′ ends of transcripts), 3′UTR, exonic, intronic and intergenic regions using bedtools v. 2.26.0 intersect function^80^.

### DNA:RNA immunoprecipitation followed by qPCR (DRIP-qPCR)

HeLa cells that were treated with either DMSO or 500 nM JQ1 for 16 h as per outlined in the methods for ChIP except that cells were harvested without being subjected to formaldehyde crosslinking. After three washes in lysis buffer, cell pellets were lysed in nuclear lysis buffer (50 mM Tris-HCl pH 8.0, 5 mM EDTA, 1% SDS) and subjected to proteolysis with Proteinase K (Thermofisher, 0.2 mg/mL final concentration) at 50 °C for 4 h. Potassium acetate was added to a final concentration of 1 M, cellular proteins precipitated, and supernatant containing chromatin was subjected to ethanol precipitation. Chromatin pellets were then reconstituted in DRIP buffer (16.67 mM Tris-HCl pH 8.0, 183.67 mM NaCl, 0.01% SDS, 1.1% Triton X-100) and subjected to Bioruptor sonication (30 sec on, 30 s off for 10 min) at 4 °C. S9.6 antibody (Kerafast) was conjugated to Protein G Dynabeads (Thermofisher) as per manufacturer’s instructions and DNA:RNA hybrid IP was performed overnight at 4^o^C. To test the specificity of the S9.6 IP, a portion of sheared chromatin was treated with RNase H (NEB, 60 U) for 3 h at 37 °C prior to immunoprecipitation. Hybrid-bound beads were washed once with DRIP buffer, followed by DRIP buffer with the addition of 500 mM NaCl, then lithium chloride buffer, and finally TE with 50 mM NaCl. Hybrids were eluted from the Dynabeads with elution buffer at 65^o^C for 15 min and treated with RNase A for 3 h at 37 °C followed by Proteinase K for 3 h at 55^o^C. Phenol:chloroform extraction was performed and hybrids isolated by ethanol precipitation. Pellets were resuspended in DNase-free, RNase-free sterile water. Equal amounts of hybrid template were subjected to qPCR with qPCR primers designed against the transcription start sites, exons, introns, and transcription termination sites of candidate genes using Fast SYBR Green master mix (ThermoFisher) on a StepOnePlus Real-Time PCR system (Applied Biosystems) in triplicate. Samples were normalized to equal amounts of template in the non-immunoprecipitated input starting material to determine relative abundance of DNA:RNA hybrids.

### Flow cytometry and cell cycle studies

For cell cycle studies, HeLa, HCT116, and RNase H-inducible HeLa cells were treated with DMSO or 500 nM JQ1 for 48 h, harvested, fixed, and stained using DAPI for DNA content. 10,000 events were recorded on a FACS LSR II HTS-1 flow cytometer (BD Biosciences). Flow data was analyzed using FlowJo software.

### DNA fiber combing assay

HeLa and HCT116 cells were treated with DMSO, 100 nM of ARV-825 for 6 hours, or 500 nM JQ1 for 16 h. Conditioned medium was removed from each plate and half of the media reserved for CldU (Sigma-Aldrich) pulse. IdU (Sigma-Aldrich) was added to the other half at a final concentration of 50 μM and added back to the plate and cells pulse-labelled with IdU for 20 min. After 20 min, IdU-containing media was aspirated, cells washed 1X with warm PBS, CldU added to the reserved media at a final concentration of 100 μM, and added to the cells for the 20 min CldU pulse. The CldU-containing media was removed after 20 minutes and cells washed in PBS, trypsinized, pelleted in ice-cold PBS, and diluted to ~200 cells per microliter. Two microliters of cells were pipetted onto Silane-prep slides (Sigma-Aldrich), lysed in 15 μL of lysis buffer (200 mM Tris-HcL pH 7.4, 50 mM EDTA, 0.5% SDS) for 10 min and slides tilted at an 25° angle to allow for DNA fiber spreads to be formed down the surface of the slides. Slides were placed horizontally, fibers allowed to dry followed by methanol:acetic acid (3:1) fixation for 2 min. Slides were then treated with 2.5 M HCl for 30 minutes followed by one wash with PBS + 0.1% Tween-20 and 2 consequent washes with PBS. Slides were blocked with 5% BSA in PBS at room temperature (RT) for 30 min, blocking solution removed and primary antibody solution containing anti-mouse monoclonal BrdU antibody (Santa Cruz Technology, Catalog No. sc-70443) and anti-rat monoclonal BrdU antibody (Santa Cruz Technology, Catalog No. sc-70441) applied across the length of the slide for 2 h at RT. Slides were washed once with PBS and stringency buffer (10 mM Tris-HCl pH 7.4, 40 mM NaCl, 0.02% Tween-20, 0.02% NP-40) placed on the slides for 10 minutes to reduce non-specific binding. Stringency buffer was then removed and slides washed twice with PBS. Slides were blocked again in blocking solution for 30 minutes at RT and solutions containing fluorophore-conjugated Alexafluor secondary antibodies rabbit anti-mouse Alexafluor 488 (Life Technologies, Catalog No. A11059) and chicken anti-rat Alexafluor 647 (Life Technologies, Catalog No. A21472) were applied to the slides for 2 h at RT. Slides were washed twice in PBS and mounted with glass coverslips using ProLong Gold Antifade mountant (Life Technologies). Approximately 100 DNA fibers were imaged per condition at ×60 magnification using an oil immersion lens on an Olympic FV1000 confocal microscope and analyzed using ImageJ software. Average fork speed was determined using the ruler tool in ImageJ to measure the total length of the fiber tracts where 1 μM corresponds to ~2.59 kilobases^81^.

### Quantitative RT-PCR

HeLa cells were treated with DMSO or 500 nM JQ1 for up to 48 h and total DNA isolated (Promega Wizard Genomic DNA Purification Kit). Reverse transcription was performed using Superscript III Reverse Transcriptase (Invitrogen) and PCR performed using Fast SYBR Green Master Mix (Invitrogen) on a StepOnePlus Real-time PCR machine (Applied Biosystems).

### Growth curves

HeLa, HCT116, and RNase H-inducible HeLa cells were treated with DMSO or 500 nM JQ1 for 72 h. Cells were harvested and cell counts performed from 3 separate experiments using a Nexcelom cellometer cell counter.

### Immunofluorescence microscopy

HeLa and HCT116 cells were grown on glass coverslips coated with Poly-l-lysine (Gibco). All cells were fixed in paraformaldehyde for 20 min, washed twice in PBS, permeabilized with PBS containing 0.1% Triton-X100, and blocked in goat serum. Immunofluorescence was performed using anti-γH2AX (1:1000), anti-S9.6 (1:200), and anti-FLAG (1:1000) primary antibodies overnight at 4 °C. Coverslips were washed three times with PBS and incubated with Alexafluor secondary antibodies (1:1000) and counterstained with DAPI (1:1000, Thermofisher) for 1 h at room temperature. Coverslips were mounted on glass slides using ProLong Gold anti-fade mountant (Invitrogen) and cured overnight. Images were captured on a Nikon Eclipse 80i fluorescence microscope. ImageJ software was used to integrate the S9.6 signal for quantitative image analysis. For specific quantification of nuclear S9.6 staining, regions of interest were overlaid with the DAPI signal, and only co-localizing regions were included in the integration to exclude cytoplasmic S9.6 signal as well as nucleolar S9.6 signal.

### Single cell gel electrophoresis

Assessment of DNA damage and DSB formation in cells was performed using the CometAssay single cell gel electrophoresis assay (Trevigen). Cells were harvested and resuspended in low-melting point agarose, plated onto provided glass slides, and subjected to electrophoresis in neutral electrophoresis buffer (100 mM Tris, 300 mM Na Acetate, pH 9.0). Slides were processed according to manufacturer’s instructions. DNA tails were visualized after SYBR Gold staining using a Nikon Eclipse 80i fluorescence microscope and quantified using ImageJ software with the OpenComet plugin (URL http://www.opencomet.org).

### Western blotting

Cells were harvested, lysed, and sonicated in low salt lysis buffer (50 mM Tris-HCl, 150 mM NaCl, 1 mM EDTA, 0.5% NP-40, pH 7.4) supplemented with protease and phosphatase inhibitors (Complete mini EDTA-free and PhosSTOP, Roche Applied Science). Total protein was concentrated down using trichloroacetic acid precipitation and reconstituted in 2X Laemmli buffer. Samples were loaded onto 15% Tris-glycine gels or 4–20% precast Tris-glycine gradient gels (Life Technologies), subjected to SDS-PAGE, and transferred onto 0.2 μm nitrocellulose membranes (BioRad). Membranes were blocked in Odyssey Blocking Buffer for 1 h and immunoblotting was performed using the monoclonal antibodies listed above at 1:1000 dilution, incubated overnight at 4 °C. Blots were washed three times in PBS-T and incubated in donkey anti-rabbit 800 or donkey anti-mouse 680 fluorescent secondary antibodies (Li-Cor) for one hour at room temperature. Protein bands were visualized using a Li-Cor Odyssey Infrared Imaging System (Li-Cor).

### Statistical analysis

Statistical analyses were performed on data generated from three or more independent experiments. Experiments were analyzed using either *T* test or ANOVA followed by a post-hoc analysis as indicated in the figure legends.

### Reporting summary

Further information on research design is available in the Nature Research Reporting Summary linked to this article.

## Supplementary information

Supplementary Information

Peer Review

Reporting Summary

## Data Availability

The data that support this study are available from the corresponding authors upon reasonable request. The sequencing data discussed in this publication have been deposited in NCBI’s Gene Expression Omnibus and are accessible through GEO Series accession number GSE151038. The source data underlying each figure are provided as a Source data file. Source data are provided with this paper.

## Source data

Source data
