# Supplementary material for: BRD4 prevents the accumulation of R-loops and protects against transcription–replication collision events and DNA damage
Source: Nat Commun. 2020 Aug 14;11:4083. doi: 10.1038/s41467-020-17503-y (PMC7428008; doi:10.1038/s41467-020-17503-y)

SUPPLEMENTARY INFORMATION

“BRD4 Prevents the Accumulation of R-loops and Protects Against Transcription-Replication Collision Events and DNA Damage”

Lam et al.

1) Supplementary Figures
2) Supplementary Table
3) Full Western Blots

Supplementary Figures

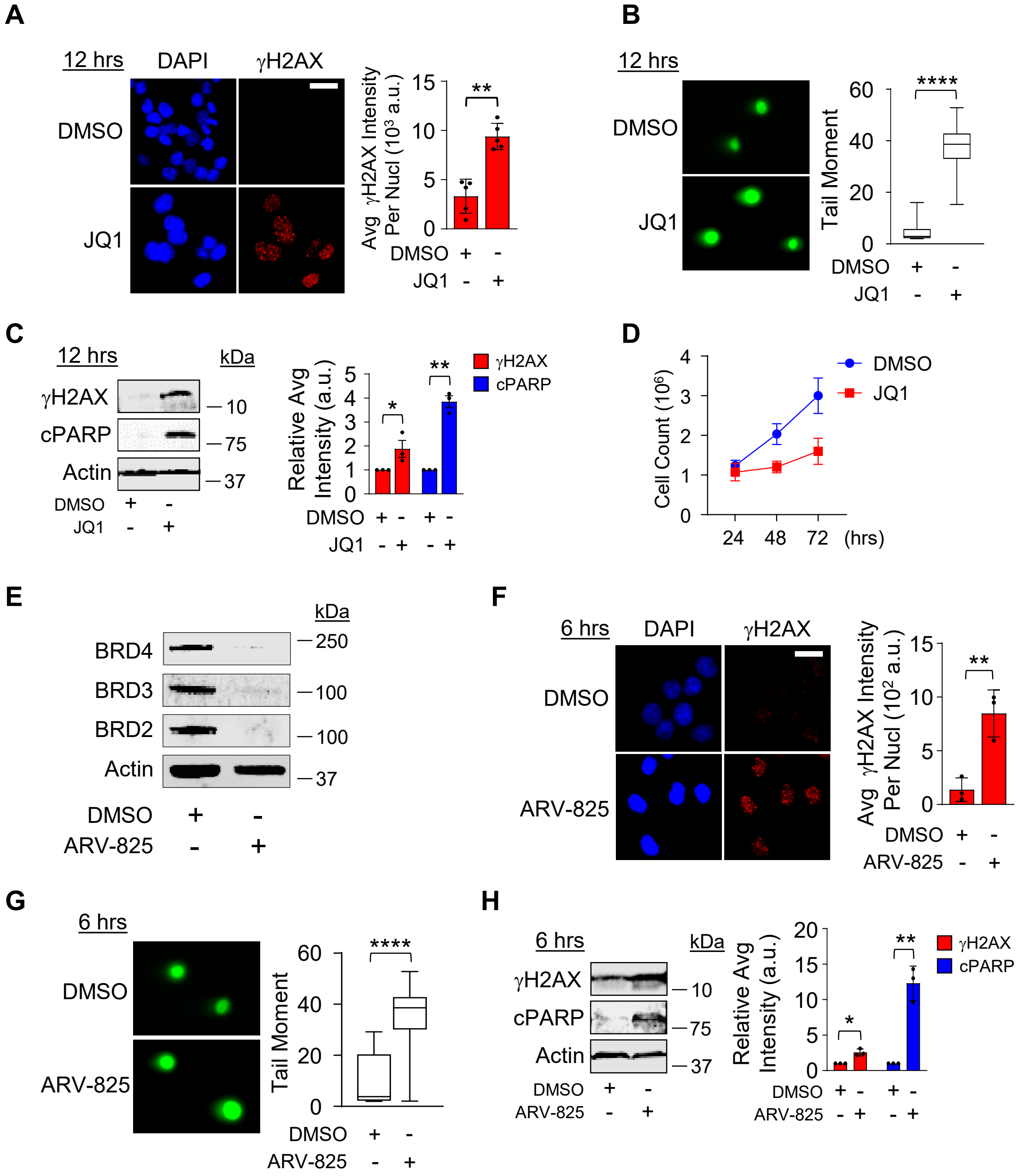

Supplementary Figure 1. Bromodomain inhibition causes DNA damage and apoptosis. (Related to Figure 1). A) Left panel: Immuno-fluorescence (IF) images of γH2AX in HCT116 cells following treatment with DMSO or 500 nMJQ1 for 12 hrs. Right panel: Total nuclear γH2AX intensity was quantified from >300 cells per condition with mean ±SEM for n = 3 independent experiments shown. Significance assessed using one-tailed unpaired *t*-test (**P < 0.01). **B)** Representative fluorescence microscopy images of neutral comet single cell gel electrophoresis assay in HCT116 cells treated for 12 hrs with DMSO or JQ1 (n = 3 independent experiments). Box-whisker plots indicate median, 25th to 75th percentiles and maximum and minimum values by line, box, and whiskers, respectively (DMSO: Min = 2.008, Max = 16.09, Q_1 = 2.407, Q_2 = 2.905, Q_3 = 5.613; JQ1: Min = 15.32, Max = 52.81, Q_1 = 33.21, Q_2 = 38.65, Q_3 = 42.62). Significance assessed using two-tailed unpaired *t*-test (****P < 0.0001). **(C)** Left panel: lysates from DMSO or JQ1-treated HCT116 cells were analyzed by WB for γH2AX and cleaved PARP (cPARP). Actin serves as a loading control. Right panel: γH2AX and cPARP band intensity was quantified and presented as mean ±SEM (n = 3 separate experiments). Significance assessed using one-tailed unpaired *t*-test (γH2AX *P = 0.0347, cPARP***P = 0.0002). **D)** Growth curve of HCT116 cells treated with DMSO (blue line) or 500 nMJQ1 (red line) for 72 hrs (mean ±SEM from n = 3 independent experiments). **E)** HCT116 cells were treated with DMSO or 100 nM ARV-825 for 6 hrs, and lysates probed for levels of BRD2, BRD3, BRD4, and actin by WB. **F)** Left: IF images of γH2AX fluorescence in HCT116 cells treated with DMSO or ARV-825. Right: Quantification of γH2AX intensity (mean ±SEM from n = 3 separate experiments). Statistical significance assessed using one-tailed unpaired *t*-test (**P = 0.0075). **G)** Representative fluorescence microscopy images of neutral comet single cell gel electrophoresis assay of HCT116 cells treated with DMSO or ARV-825 for 6 hrs (n = 3 independent experiments). Box-whisker plots as in panel **B** (DMSO: Min = 2.008, Max = 29.23, Q_1 = 2.462, Q_2 = 3.812, Q_3 = 20.13; ARV-825: Min = 2.022, Max = 52.81, Q_1 = 30.38, Q_2 = 38.60, Q_3 = 42.57). Significance assessed using two-tailed unpaired *t*-test (****P < 0.0001). **H)** Left panel: Lysates from HCT116 cells treated with ARV-825 were analyzed for γH2AX, cPARP, and actin by WB. Right panel: Intensity of the γH2AX and cPARP bands was quantified and presented as mean ±SEM from n = 3 separate experiments. Significance assessed using two-tailed unpaired *t*-test (γH2AX *P = 0.0309, cPARP **P = 0.0013). Scale bars in **A** and **F** = 5 µm. Source data are provided as a Source Data file.

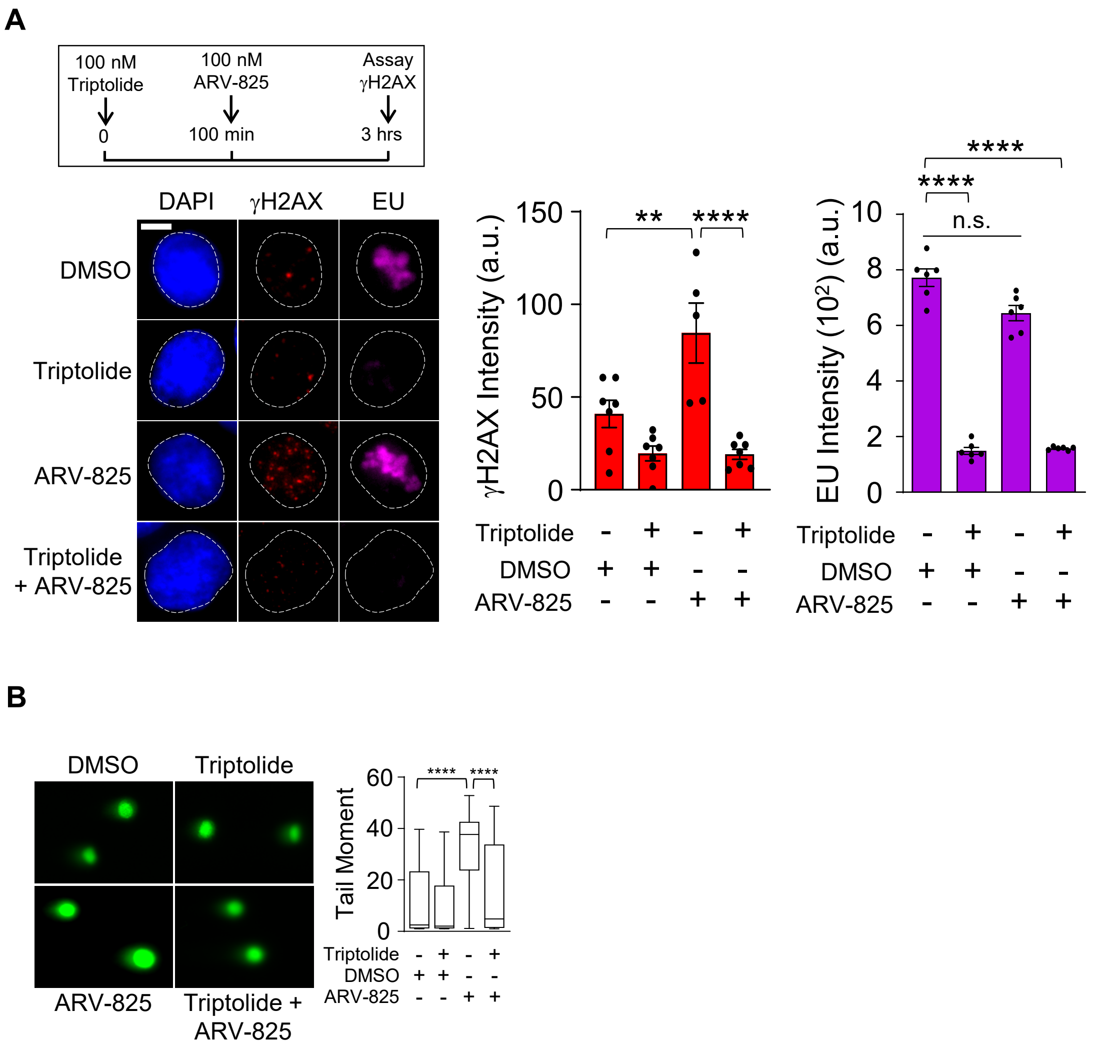

Supplementary Figure 2. DNA damage following BRD4 loss requires active transcription in HCT116 cells. (Related to Figure 2). A) Left top panel: Schematic of triptolide/ARV-825 co-treatment in HCT116 cells. Bottom left: Representative IF images of HCT116 cells following treatment with triptolide and/or ARV-825. EU incorporation was used to assess RNA synthesis. Right: Quantification of integrated γH2AX and EU intensity per nucleus quantified from >300 cells per condition as in Figure 1A (mean ± SEM from n = 3 independent experiments). Significance assessed using ANOVA followed by Tukey's test ($\gamma H2AX_{DMSO\ vs ARV\text{-}825}$ **Adjusted P = 0.0051, 95% CI = -755.3 to -117.1; $\gamma H2AX_{ARV\text{-}825\ vs\ Triptolide\ +\ ARV\text{-}825}$ ****Adjusted P < 0.0001, 95% CI = 335.5 to 973.7; $EU_{DMSO\ vs\ Triptolide}$ ****Adjusted P < 0.0001, 95% CI = 536.4 to 709.9; $EU_{DMSO\ vs\ Triptolide+\ ARV\text{-}825}$ ****Adjusted P < 0.0001, 95% CI = 527.9 to 701.4). Scale bar = 2.5 µm. **B)** Left: Representative fluorescence microscopy of neutral comet single cell gel electrophoresis assay showing tail moments in HCT116 cells pretreated with triptolide and co-treated with ARV-825. Box-whisker plots drawn as in Suppl. Fig. 1B (DMSO: Min = 1.034, Max = 39.70, Q_1 = 1.326, Q_2 = 2.48, Q_3 = 23.15; Triptolide: Min = 1.002, Max = 38.68, Q_1 = 1.327, Q_2 = 2.024, Q_3 = 38.68; ARV-825: Min = 1.158, Max = 52.81, Q_1 = 23.88, Q_2 = 37.66, Q_3 = 42.41; Triptolide+ ARV-825: Min = 1.012, Max = 48.70, Q_1 = 1.555, Q_2 = 4.822, Q_3 = 33.61), n = 3 independent experiments. Significance assessed using ANOVA followed by Tukey's test ($Tail\ Moment_{DMSO\ vs\ ARV\text{-}825}$ ****Adjusted P < 0.0001, 95% CI = -28.43 to -14.95; $Tail\ Moment_{ARV\text{-}825\ vs\ Triptolide+\ ARV\text{-}825}$ ****Adjusted P < 0.0001, 95% CI = 8.048 to 22.15). Source data are provided as a Source Data file.

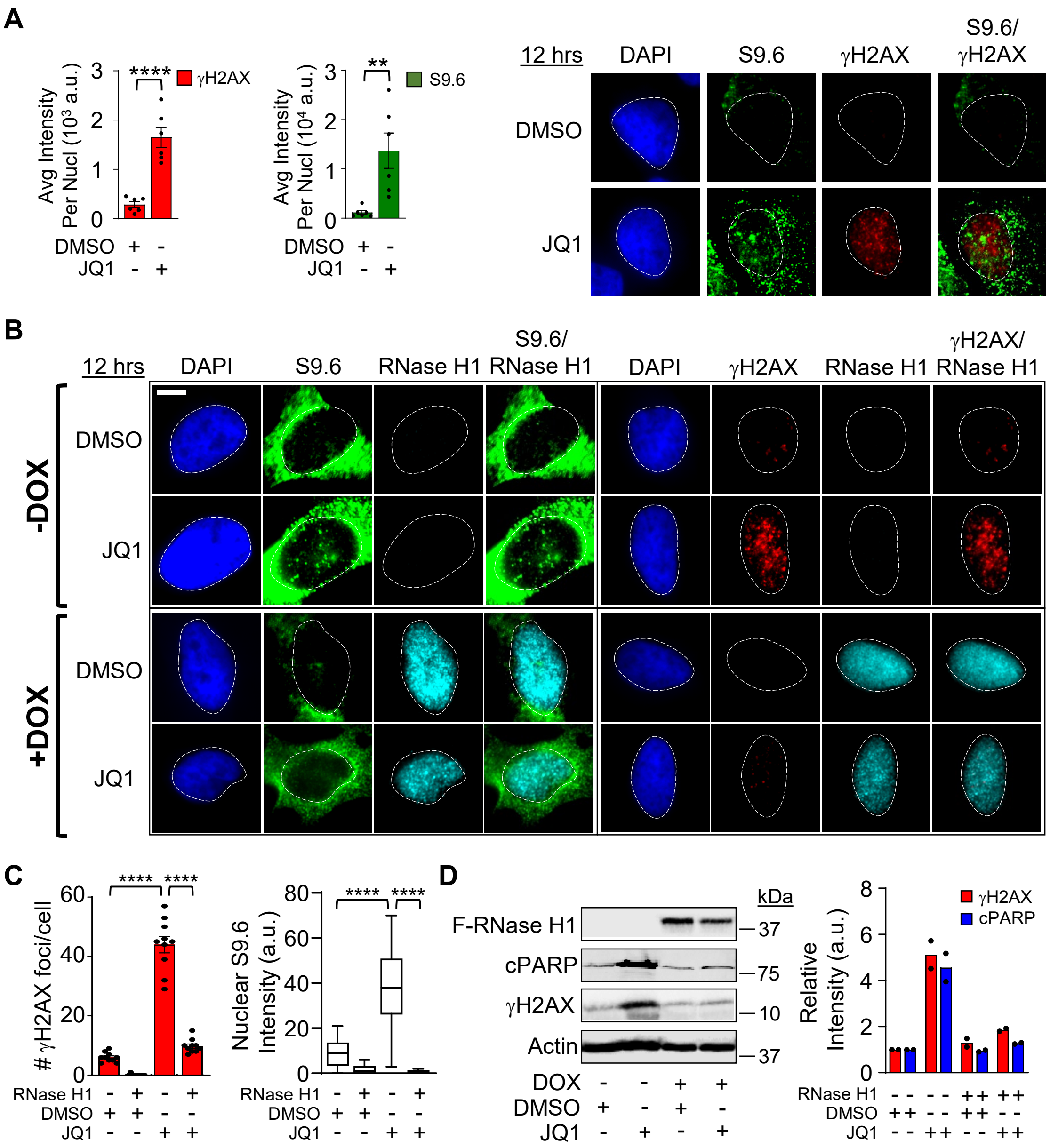

Supplementary Figure 3. DNA damage caused by BRD4 inhibition is dependent on R-loops. (Related to Figure 3). **A)** Quantification and representative IF images of nuclear S9.6 and γH2AX intensity in HeLa cells following treatment with DMSO or 500 nM JQ1 for 12 hrs. Quantification of nuclear S9.6 intensity performed as described in Fig. 3A. Data presented as mean ± SEM (n = 3 independent experiments). Significance assessed using two-tailed unpaired *t* test (γH2AX ****P < 0.0001; S9.6 **P = 0.0060). **B, C)** Representative IF images (B) and quantification of γH2AX foci and nuclear S9.6 intensity (C) in HeLa cells expressing inducible FLAG-RNase H1 the absence (-DOX) or presence (+DOX) of doxycycline treatment for 24 hrs, followed by treatment with JQ1 for 12 hrs. Data presented as mean ± SEM, n = 3 independent experiments. Box-whisker plots represent middle 50% of scores ($DMSO_{RNaseH1\ (-)}$: Min = 0, Max = 21, Q_1 = 3.75, Q_2 = 9, Q_3 = 13.25; $DMSO_{RNaseH1\ (+)}$: Min = 0, Max = 6, Q_1 = 0, Q_2 = 1, Q_3 = 3; $JQ1_{RNaseH1\ (-)}$: Min = 3, Max = 70, Q_1 = 26.5, Q_2 = 38, Q_3 = 50.50; $JQ1_{RNaseH1\ (+)}$: Min = 0, Max = 2, Q_1 = 0, Q_2 = 0, Q_3 = 1). Significance assessed using ANOVA followed by Tukey's test ($\gamma H2AX_{DMSO\ RNaseH1\ (-)\ vs\ JQ1\ RNaseH1\ (-)}$ ****Adjusted P < 0.0001, 95% CI = -43.53 to -32.47; $\gamma H2AX_{JQ1\ RNaseH1\ (-)\ vs\ JQ1\ RNaseH1\ (+)}$ ****Adjusted P < 0.0001, 95% CI = 28.67 to 39.73; $S9.6_{DMSO\ RNaseH1\ (-)\ vs\ JQ1\ RNaseH1\ (-)}$ ****Adjusted P < 0.0001, 95% CI = -34.95 to -24.63; $S9.6_{JQ1\ RNaseH1\ (-)\ vs\ JQ1\ RNaseH1\ (+)}$ ****Adjusted P < 0.0001, 95% CI = 31.44 to 43.87). **D)** WB (left) and quantification (right) of γH2AX and cPARP in lysates from cells treated with JQ1 upon expression of RNase H1 (F-RNase H1). Data presented as mean (n = 2 independent experiments). Actin serves as a loading control. Scale bars in **A** and **B** = 2.5 μm. Source data are provided as a Source Data file.

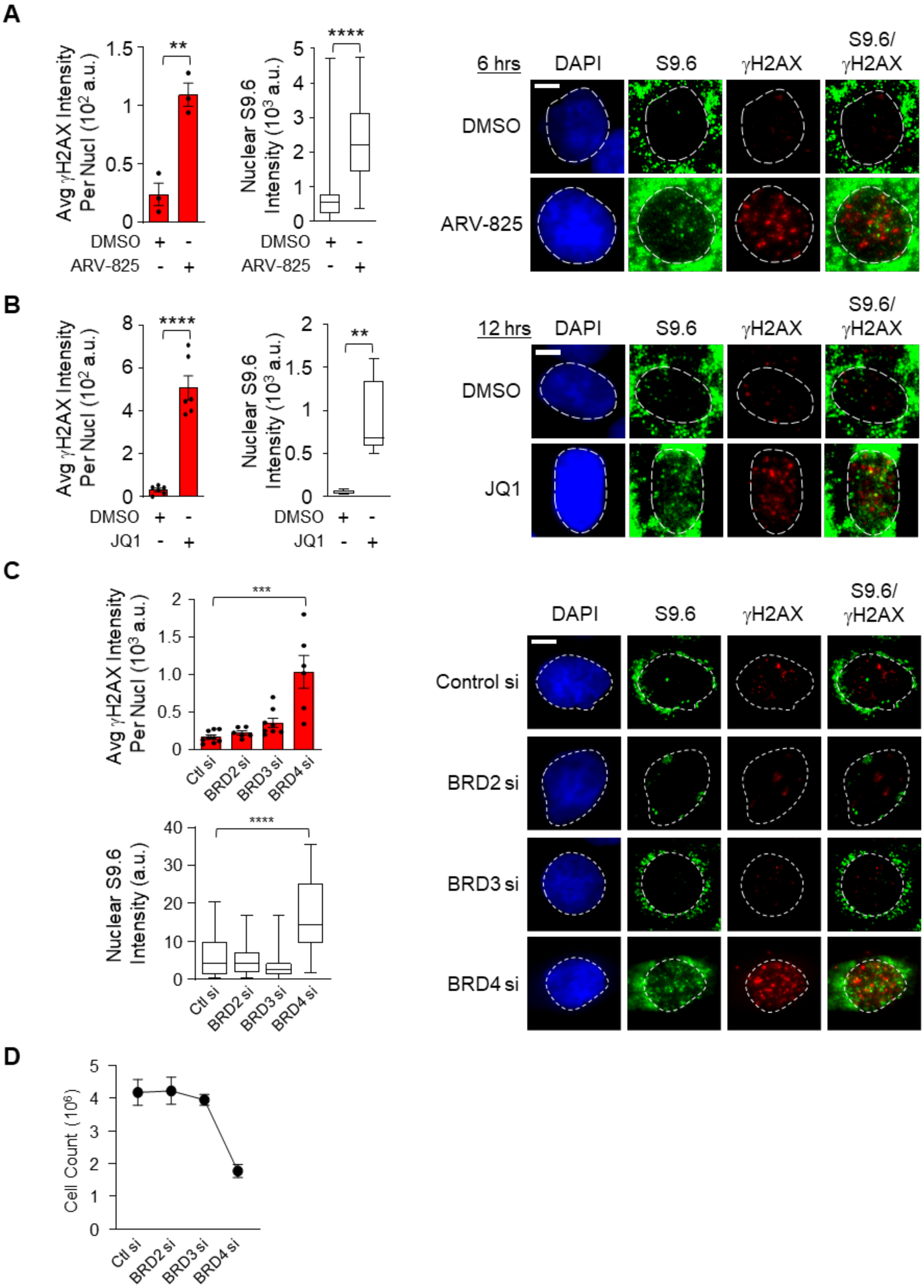

Supplementary Figure 4. BRD4 loss leads to R-loop-induced DNA damage in HCT116 cells. (Related to Figures 4&5.) A, B) Quantification (left) and representative IF images (right) of nuclear S9.6 and γH2AX intensity in HCT116 cells treated with DMSO or ARV-825 for 6 hrs (A) or DMSO or JQ1 for 12 hrs (B) from n = 3 independent experiments. γH2AX data shown as mean ±SEM. S9.6 intensity shown as box-whisker plots drawn as in Suppl. Fig. 1B. Plot in **A:** (DMSO: Min = 0, Max = 47033, Q_1 = 2265, Q_2 = 5556, Q_3 = 7762; ARV-825: Min = 3756, Max = 47349, Q_1 = 14457, Q_2 = 22196, Q_3 = 31175). Plot in **B:** DMSO: Min = 31.14, Max = 89.15, Q_1 = 35.08, Q_2 = 39.54, Q_3 = 74.16; JQ1: Min = 502.5, Max = 1604, Q_1 = 585.2, Q_2 = 680.7, Q_3 = 1340). Significance assessed using two-tailed unpaired *t*-test in **A** (γH2AX **P = 0.0035, S9.6 ****P < 0.0001) and **B** (γH2AX ****P < 0.0001, S9.6 **P = 0.0026). **C)** Quantification (left) and representative IF images (right) of nuclear S9.6 and γH2AX fluorescence in HCT116 cells following transfection of control siRNA or siRNAs against BRD2, BRD3, or BRD4. γH2AX data shown as mean ±SEM. S9.6 intensity shown as box-whisker plot as in panels **A** and **B**, (Control si: Min = 501, Max = 20424, Q_1 = 1645, Q_2 = 4299, Q_3 = 9857; BRD2 si: Min = 405, Max = 17003, Q_1 = 2068, Q_2 = 4146, Q_3 = 6917; BRD3 si: Min = 229.7, Max = 16805, Q_1 = 1404, Q_2 = 2702, Q_3 = 4182; BRD4 si: Min = 1732, Max = 35497, Q_1 = 9765, Q_2 = 14310, Q_3 = 25198). Significance assessed using ANOVA followed by Tukey's test ($\gamma H2AX_{Ctl\ si\ vs\ BRD4\ si}$ ***Adjusted P = 0.0002, 95% CI = -1155 to -542.6; $S9.6_{Ctl\ si\ vs\ BRD4\ si}$ ****Adjusted P < 0.0001, 95% CI = -15370 to -5902). **D)** Cell counts measured at 72 hrs following transfection of control siRNA or siRNAs against BRD2, BRD3, or BRD4 from n = 4 independent experiments shown as mean ± SEM. Scale bars in all panels = 2.5 µm. Source data are provided as a Source Data file.

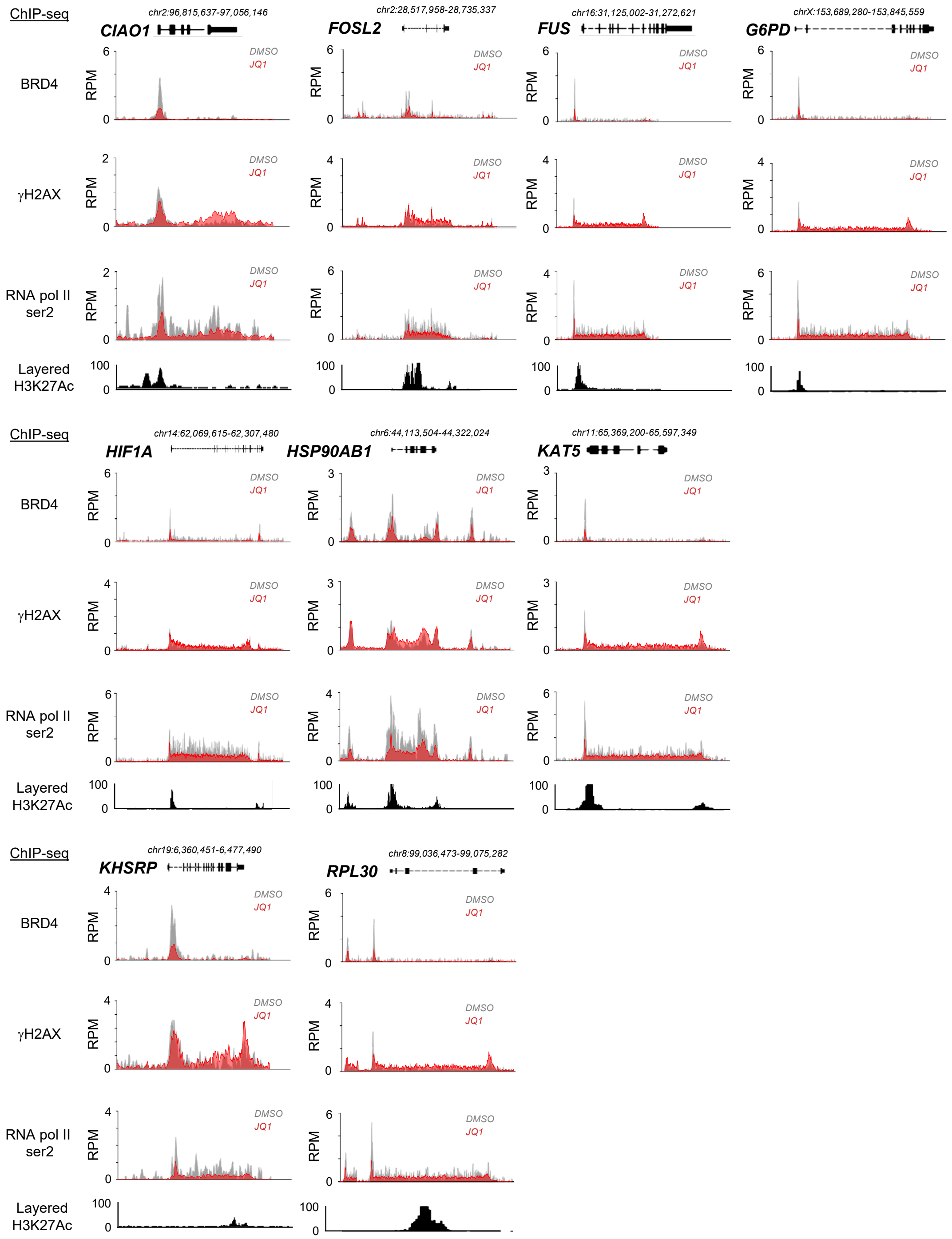

Supplementary Figure 5. BRD4 inhibition increases DNA damage at BRD4, JMJD6, and CHD4 co-regulated genes. (Related to Figure 7). BDR4, γH2AX, and RNAPII ser2 ChIP-Seq plots at select gene loci following treatment with 500 nM JQ1 for 12 hrs. Layered H3K27Ac ChIP-Seq plots from ENCODE database signify regions of active transcription. BRD4 regulated genes: *KAT5, KHSRP, RPL30*. BDR4 and CHD4 co-regulated genes: *CIAO1*, *FOSL2, FUS, HSP90AB01*. BRD4 and JMJD6 co-regulated genes: *CIAO1, G6PD, HIF1A*. Source data are provided as a Source Data file.

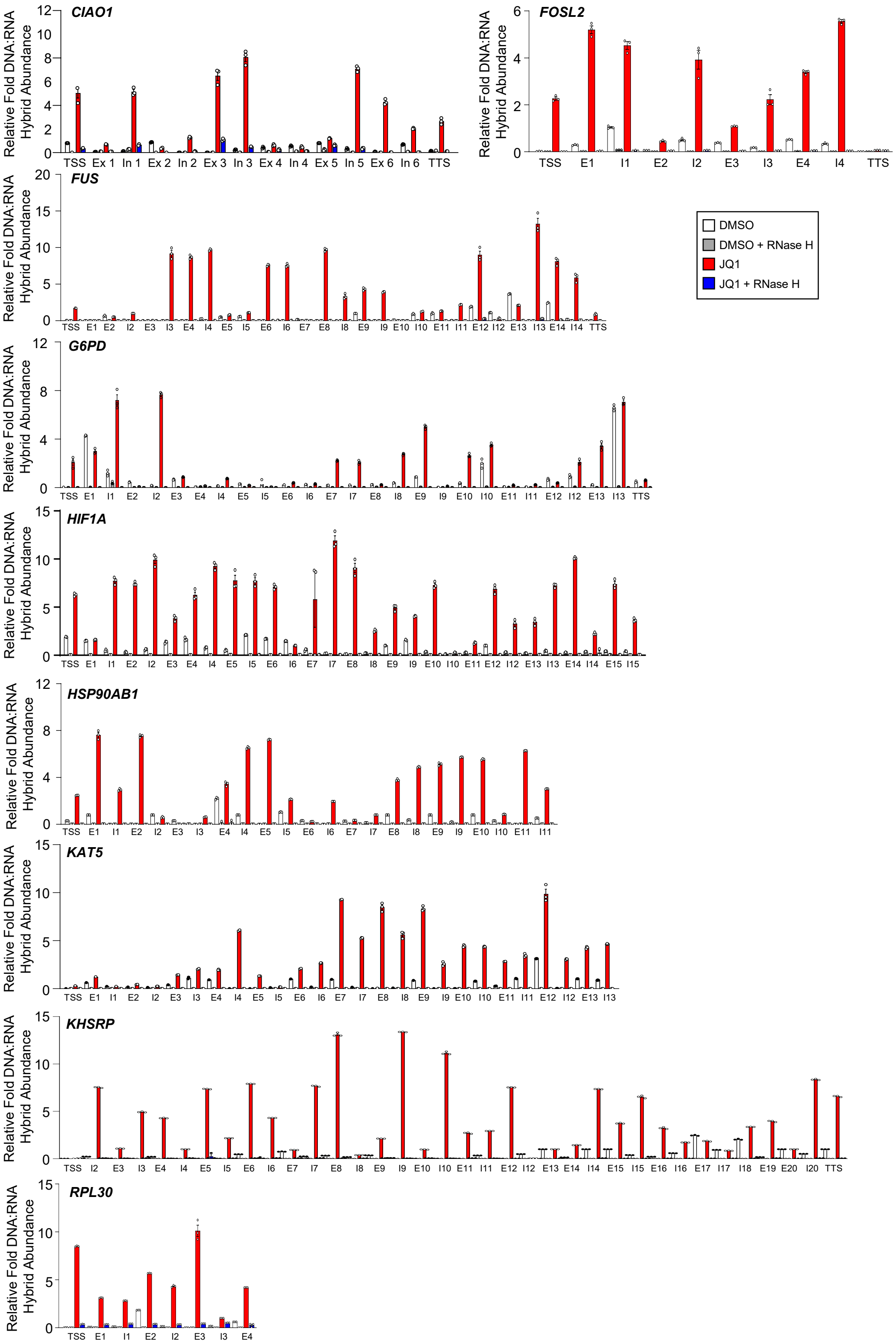

Supplementary Figure 6. BRD4 inhibition increases accumulation of R-loops at BRD4, JMJD6, and CHD4 co-regulated genes. (Related to Figure 7). Quantification of relative abundance of DNA:RNA hybrids spanning the gene loci reported in Figure S5 following treatment with DMSO (white bars), DMSO+RNase H1 (gray bars), JQ1 (red bars), JQ1+RNase H1 (blue bars) using DRIP-qPCR (TSS = transcription start site, E = exon, I = intron, TTS = transcription termination site). Data presented as mean ± SEM (n = 3 independent experiments). Source data are provided as a Source Data file.

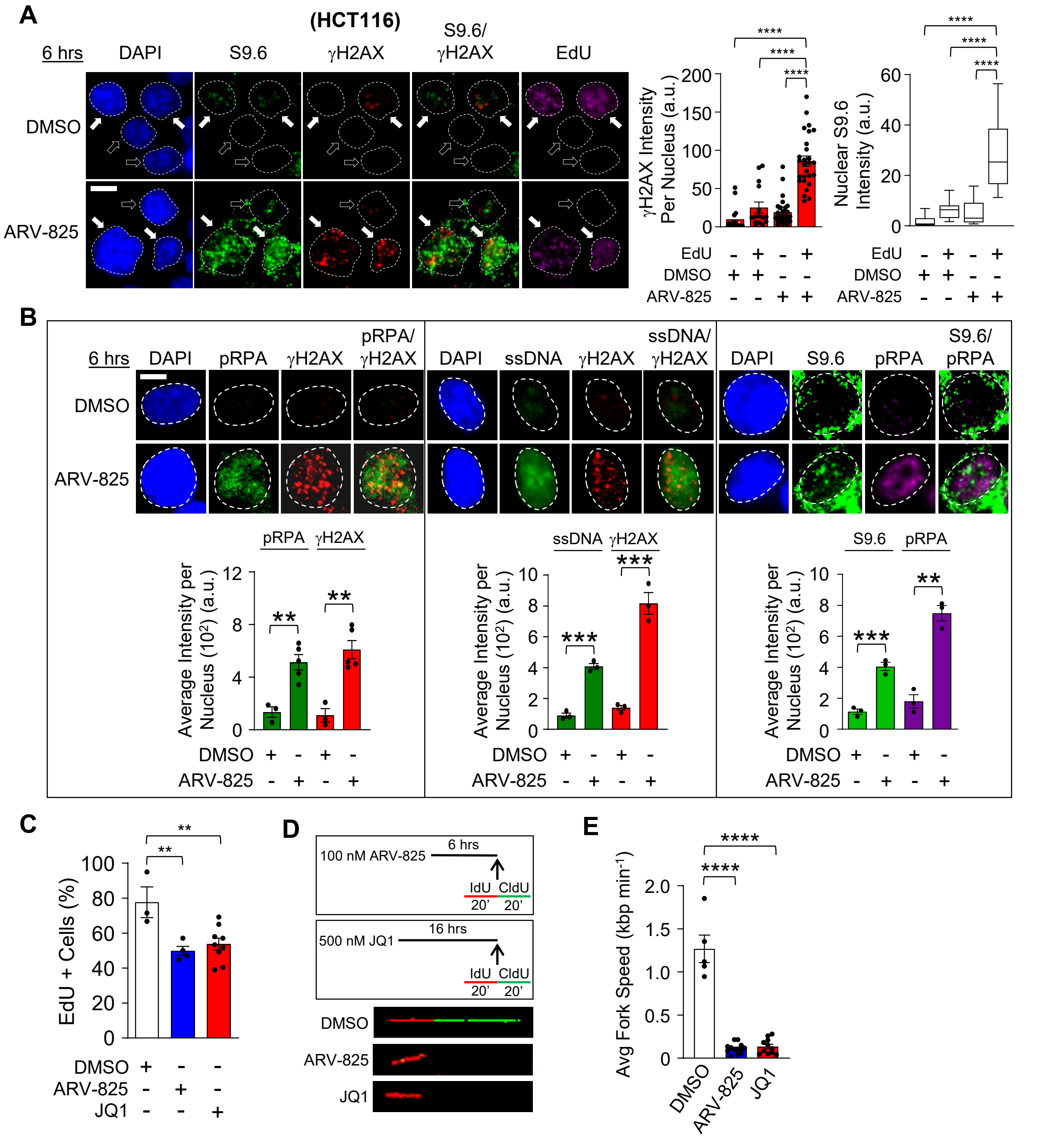

Supplementary Figure 7. BRD4 loss increases DNA damage signaling in S phase cells, replication stress, and fork stalling. (Related to Figure 8). **A)** IF (left) and quantification (right) of nuclear γH2AX and S9.6 fluorescence intensity in EdU(-) or EdU(+) (solid arrows) HCT116 cells following treatment with DMSO or 100 nM ARV-825 for 6 hrs (n = 3 independent experiments). γH2AX data shown as mean ± SEM. S9.6 intensity data shown as box-whisker plots as in Suppl. Fig. 1B ($DMSO_{EdU(-)}$: Min = 157.5, Max = 3427, Q_1 = 246.8, Q_2 = 391.8, Q_3 = 1448; $DMSO_{EdU(+)}$: Min = 843.5, Max = 7062, Q_1 = 1649, Q_2 = 3199, Q_3 = 3956; $ARV\text{-}825_{EdU(-)}$: Min = 399.7, Max = 7874, Q_1 = 780.7, Q_2 = 1517, Q_3 = 4489; $ARV\text{-}825_{EdU(+)}$: Min = 5625, Max = 28196, Q_1 = 8371, Q_2 = 12671, Q_3 = 28196). Significance assessed using ANOVA followed by Tukey's test ($γH2AX_{DMSO\ EdU(-)\ vs\ ARV\text{-}825\ EdU(+)}$ ****Adjusted P < 0.0001, 95% CI = -9734 to -5474; $γH2AX_{DMSO\ EdU(+)\ vs\ ARV\text{-}825\ EdU(+)}$ ****Adjusted P < 0.0001, 95% CI = -8248 to -3900; $γH2AX_{ARV\text{-}825\ EdU(-)\ vs\ ARV\text{-}825\ EdU(+)}$ ****Adjusted P < 0.0001, 95% CI = -8504 to -4862; $S9.6_{DMSO\ EdU(-)\ vs\ ARV\text{-}825\ EdU(+)}$ ****Adjusted P < 0.0001, 95% CI = -16339 to -9702; $S9.6_{DMSO\ EdU(+)\ vs\ ARV\text{-}825\ EdU(+)}$ ****Adjusted P < 0.0001, 95% CI = -13999 to -7463; $S9.6_{ARV\text{-}825\ EdU(-)\ vs\ ARV\text{-}825\ EdU(+)}$ ****Adjusted P < 0.0001, 95% CI = -14808 to -7640). Scale bar = 5 µm. **B)** IF images (top) and quantification (bottom) of pRPA2 ser33 (pRPA), γH2AX, native BrdU (ssDNA), and S9.6 immunofluorescence in cells treated with DMSO or ARV-825. Data shown as mean ± SEM from n = 3 independent experiments. Scale bar = 2.5 µm. Significance assessed using two-tailed unpaired *t* test in left panel (pRPA **P = 0.0038, γH2AX **P = 0.0024); middle panel (ssDNA ***P = 0.0002, γH2AX ***P = 0.0007); and right panel (S9.6 ***P = 0.0008, pRPA **P = 0.0010). **C)** Quantification of the percentage of EdU(+) cells following treatment with DMSO (black), ARV-825 (blue), or JQ1 (red) with mean ± SEM from n = 3 independent experiments. Significance assessed using ANOVA followed by Tukey's test (DMSO vs ARV-825 **P = 0.0098, 95% CI = 6.010 to 41.99; DMSO vs JQ1 **P = 0.0089, 95% CI = 7.277 to 48.50). **D)** Experimental scheme for DNA fiber combing experiments in HCT116 cells. Representative fluorescent images of DNA fibers following treatment with either DMSO, ARV-825, or JQ1 showing normal replication and two examples of fork stalling. **E)** Quantification of fork speed following treatment with DMSO (black), ARV-825 (blue), or JQ1 (red) with mean ±SEM shown from n = 3 independent experiments). Significance assessed using ANOVA followed by Tukey's test (DMSO vs ARV-825 ****P < 0.0001, 95% CI = 0.9518 to 1.359; DMSO to JQ1 ****P < 0.0001, 95% CI = 9.247 to 1.343). Source data are provided as a Source Data file.

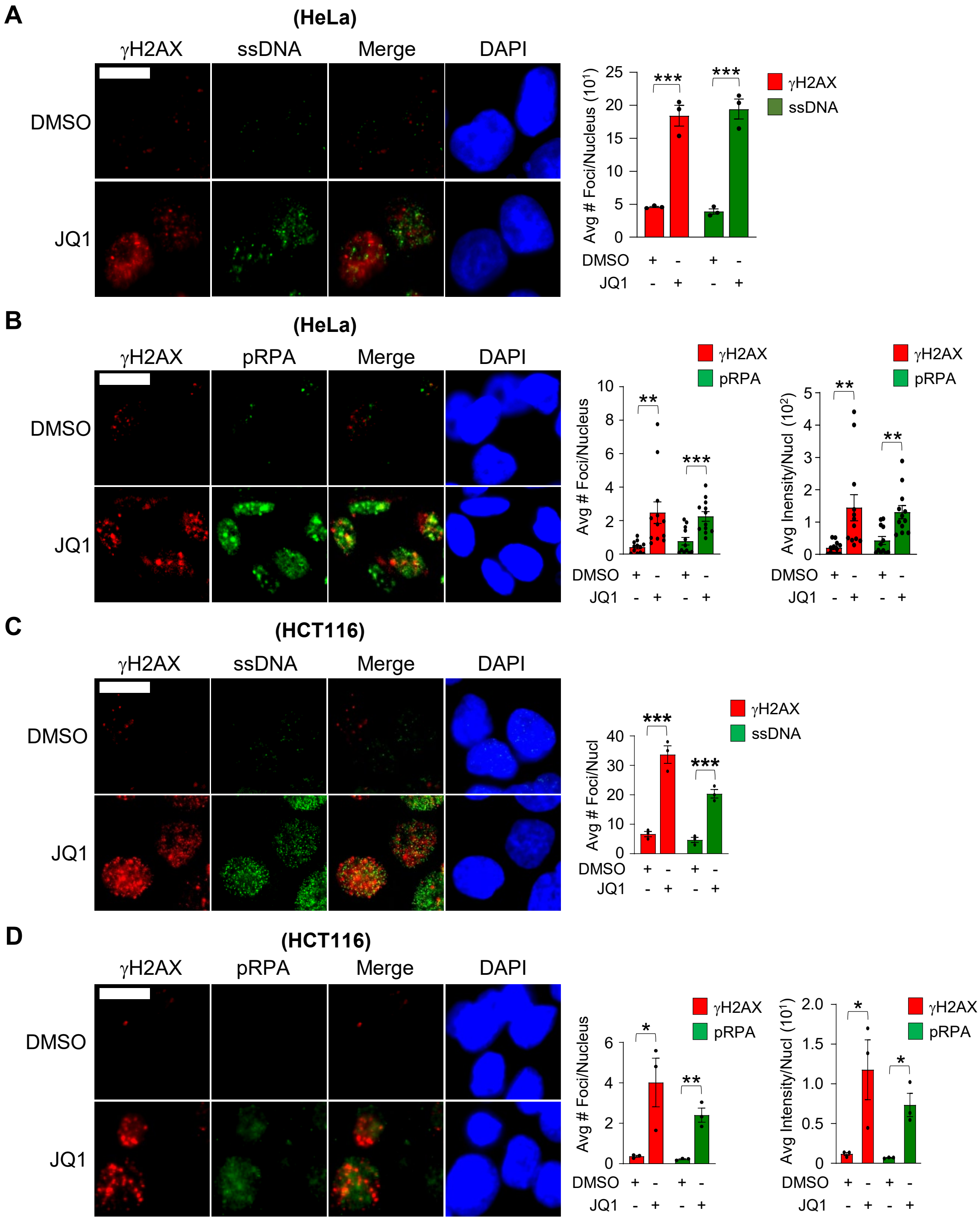

Supplementary Figure 8. BET bromodomain inhibition increases markers of replication stress. (Related to Figure 8). A&C) Representative IF images (left) and quantification (right) of nuclear staining of γH2AX and native BrdU (ssDNA), and **B&D)** γH2AX and pRPA immunofluorescence in cells treated with either DMSO or JQ1. Data in all panels quantified as in Fig. 1A and presented as mean ± SEM (n = 3 independent experiments). Significance assessed using two-tailed unpaired *t*-test in **A** (γH2AX foci/nucleus ***P = 0.0009; ssDNA foci/nucleus ***P = 0.0006); **B** (γH2AX foci/nucleus **P = 0.0046; pRPA foci/nucleus ***P = 0.0005; γH2AX intensity/nucleus **P = 0.0059; pRPA intensity/nucleus **P = 0.0012); **C** (γH2AX foci/nucleus ***P = 0.0009; pRPA foci/nucleus ***P = 0.0008); and **D** (γH2AX foci/nucleus *P = 0.0389; pRPA **P = 0.0034; γH2AX intensity/nucleus *P = 0.0485; pRPA intensity/nucleus *P = 0.0125). Scale bars = 5 μm in all panels. Source data are provided as a Source Data file.

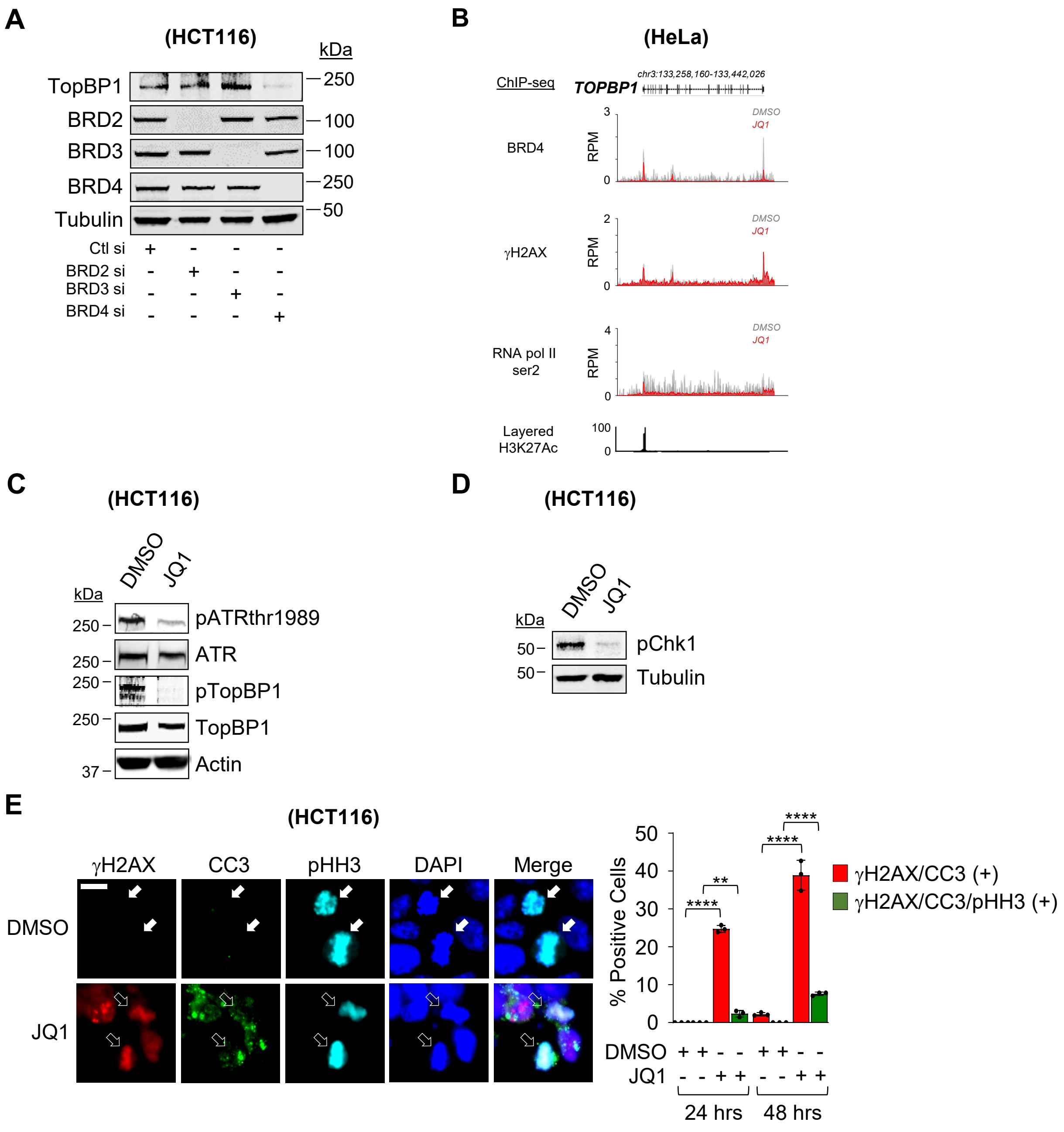

Supplementary Figure 9. BET bromodomain inhibition leads to downregulation of the ATR-TopBP1-Chk1 DNA damage checkpoint. (Related to Figure 10). A) WB of TopBP1, BRD2, BRD3, BRD4, and tubulin following transfection of control siRNA, or siRNA against BRD2, BRD3, or BRD4 in HCT116 cells. **B)** BRD4, γH2AX, RNAPII ser2 ChIP-Seq plots at the *TOPBP1* loci following treatment with DMSO (grey) or JQ1 (red) in HeLa cells. **C,D)** WB of pATRthr1989, ATR, pTopBP1, TopBP1, and actin (C) and pChk1ser345 and tubulin (D), following treatment with DMSO or JQ1 in HCT116 cells. **E)** IF images (left) and quantification (right) of nuclear γH2AX, CC3, and pHH3 immunostaining in HCT116 cells following treatment with DMSO or JQ1. Data was analyzed as in Fig. 1A and presented as mean ± SEM (n = 3 independent experiments). Significance assessed using two-tailed unpaired *t* test (24 hrs $\mathrm{DMSO}_{\gamma H2AX/CC3\ (+)}$ vs $\mathrm{JQ1}_{\gamma H2AX/CC3\ (+)}$ ****P < 0.0001; 48 hrs $\mathrm{DMSO}_{\gamma H2AX/CC3\ (+)}$ vs $\mathrm{JQ1}_{\gamma H2AX/CC3\ (+)}$ ****P < 0.0001; 24 hrs $\mathrm{DMSO}_{\gamma H2AX/CC3/pHH3\ (+)}$ vs $\mathrm{JQ1}_{\gamma H2AX/CC3/pHH3\ (+)}$ **P = 0.0084; 48 hrs $\mathrm{DMSO}_{\gamma H2AX/CC3/pHH3\ (+)}$ vs $\mathrm{JQ1}_{\gamma H2AX/CC3/pHH3\ (+)}$ **** P < 0.0001). Scale bar = 5 µm. Source data are provided as a Source Data file.

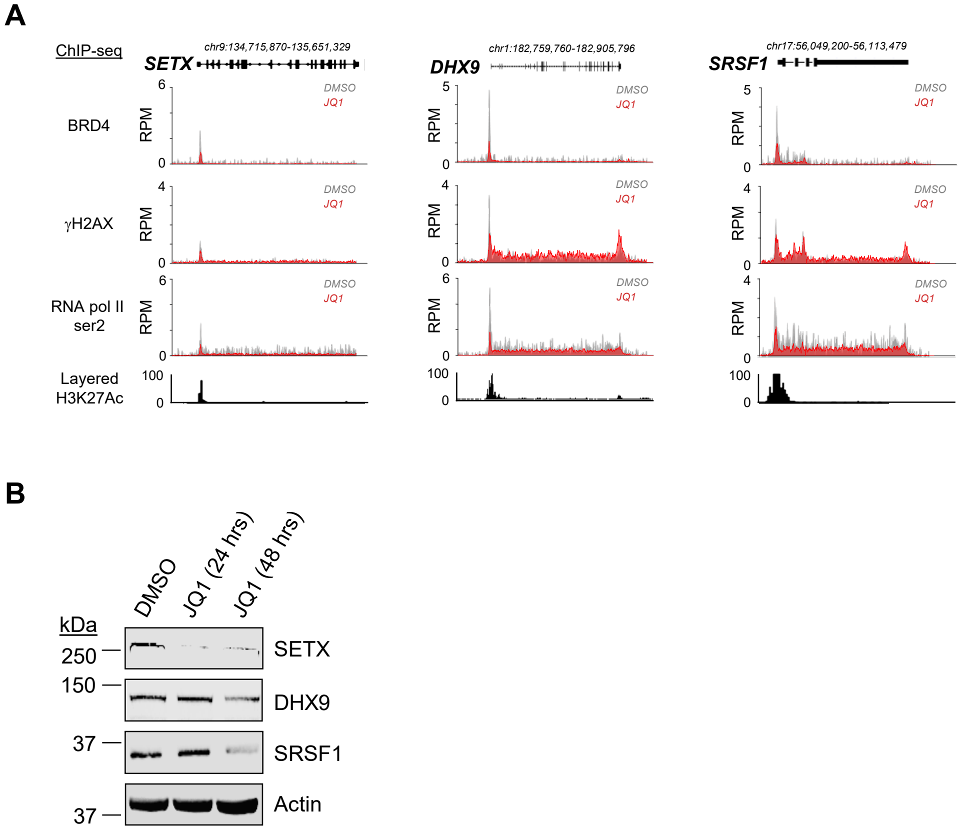

Supplementary Figure 10. BRD4 regulates transcription of R-loop processing proteins. A) BRD4, γH2AX, RNAPII ser2 ChIP-Seq plots at the *SETX, DHX9,* and *SRSF1* loci following treatment with DMSO (grey) or JQ1 (red) in HeLa cells. **B)** WB of SETX, DHX9, SRSF1, and actin following 24 and 48 hrs treatment with JQ1 in HeLa cells. Source data are provided as a Source Data file.

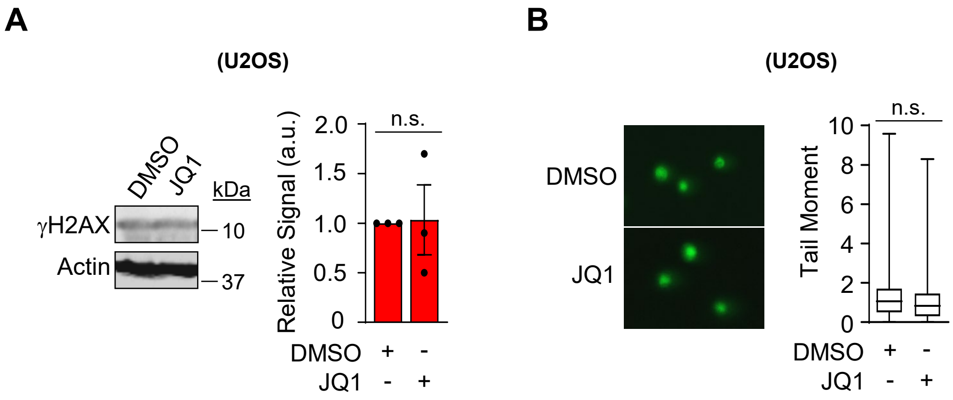

Supplementary Figure 11. BET bromodomain inhibition does not increase DNA damage or DSB formation in U2OS cells. A) WB of γH2AX and actin (left), and quantification of γH2AX band intensity relative to DMSO treatment (right) in cells treated with either DMSO of 1 μM of JQ1 for 48 hrs. Data presented as mean ± SEM (n = 3 independent experiments). **B)** Example of fluorescence microscopy images of neutral comet single cell gel electrophoresis assay showing tail moments indicating DSBs in U2OS cells treated with either DMSO or 1 μM of JQ1 for 48 hrs (n = 3 independent experiments). Box-whisker plots drawn as in Suppl. Fig. 1B (DMSO: Min = 0, Max = 9.582, Q_1 = 0.5097, Q_2 = 1.057, Q_3 = 1.706; Triptolide: Min = 1.002, Max = 38.68, Q_1 = 1.327, Q_2 = 2.024, Q_3 = 38.68; JQ1: Min = 0, Max = 8.299, Q_1 = 0.3091, Q_2 = 0.8332, Q_3 = 1.464). Statistical significance in panels **A** and **B** assessed using two-tailed unpaired *t* test (n.s. = not significant). Source data are provided as a Source Data file.

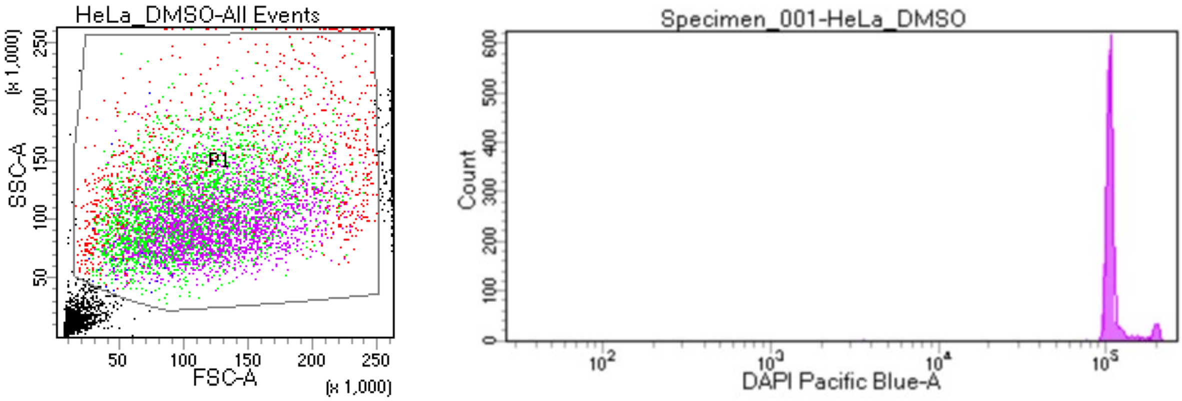

Supplementary Figure 12. Representative FACS sequential gating strategy for cell cycle analysis. (Related to Figure 9). Left panel demonstrates the gating strategy on a SSC vs FSC plot following capture of 10,000 events on a FACS LSR-II HTS-1 flow cytometer (BD Biosciences). This gate setting used in all subsequent sample runs. Right panel demonstrates typical cell cycle plot (DAPI) obtained using these gate settings

Supplementary Table

BRD4-regulated genes that show increased DNA damage and R-loop accumulation following bromodomain inhibition

BRD4 Regulated Genes	BRD4 & JMJD6 Regulated Genes				BRD4 & CHD4 Regulated Genes		
ABHD14B	ABHD14A-ACY1	ELOVL1	MRPS25	RPS15A	ABCD1	GALE	PNN
ACAT1	ACTN4	ENSA	MRPS34	RPS24	ABHD1	GEMIN8	POLR2L
ACY1	ACTR1B	EXOC7	MSL1	RPS6	ABHD14B	GGA2	POP5
ANXA2	AEN	EXOSC1	MSX1	RRAGA	ABHD4	GIGYF1	PPP1R15A
ARMCX3	AHSA2	EZR	MSX2	RSBN1	ABHD5	GLI3	PPP2R1A
ATXN2L	AIP	FAM127B	MXD3	SCARB2	ACTN4	GLOD4	PRDX1
BCLAF1	ALDOA	FAM69B	MDX4	SEC62	ACTR1B	GNAS	PREB
BIRC5	AMH	FASN	MYBBP1A	SEMA3C	ACY1	GPATCH4	PRMT1
C11orf58	ANP32B	FBXO21	MYEOV2	SERINC1	AHSA2	GPS1	PSAP
C5orf45	AP1M1	FOXK1	MYH10	SF3B14	ALDOA	GRSF1	PSMD6
CCNB1	APEX1	FOXP1	MYH9	SF3B2	AMH	GSPT1	PSMD8
CDKN1A	APRT	FOXRED1	MYL12A	SKIL	ANXA2	GTPBP4	PTBP1
CDKN1B	ARFGAP1	FTSJ1	MYL6	SLC25A39	AP1M1	H1F0	PTDSS2
CHPF2	ARID5B	FUS	MYLIP	SLC38A2	APOA1BP	HMBS	PTMA
CKS2	ARRDC3	G6PD	NAPA	SCL7A5	APRT	HMGN2	PURA
CLK1	ATIC	GALE	NCRNA00245	SCL9A3R1	ARID5B	HMOX2	RAC3
DLEU2	ATP5B	GEMIN8	NDUFA11	SMAD7	ATIC	HNRNPL	RFWD3
EIF2S	ATP5I	GGA2	NDUFAF3	SMARCD1	ATPIF1	HNRNPM	RHOB
FOSL2	ATP5J	GHITM	NDUFB10	SNHG9	ATXN2L	ID2	RNASEH2C
GPATCH4	ATPIF1	GIGYF1	NDUFS6	SNRPA	B4GALT1	IDH3B	RNF167
KIF20A	B4GALT1	GLI3	NEAT1	SNX5	BCOR	ILF3	RPL36
MRPS22	BCOR	GLOD4	NELF	SP3	BIRC5	INSIG1	RPL38
NCRNA00201	BRP44L	GNAS	NFE2L1	SPCS1	BRP44L	KDERL2	RPN2
PABPC1	C11orf57	GPS1	NHP2L1	SPSB3	C17orf85	KHSRP	RPS24
PAPOLA	C14orf2	GPS2	NME3	SQSTM1	C19orf28	KIAA0644	RRAGA
PGRMC2	C17orf48	GRSF1	NPTX1	SRSF7	CALM1	KRT8	RSBN1
RABGGTB	C17orf85	GSPT1	NR2F2	STK25	CARHSP1	KIAA0644	SAP30L
RFWD3	C19orf28	GTPBP4	NT5C	STX16-NPEPL1	CBS	KRT8	SCARB2
RPL38	C1orf35	H1F0	NT5DC2	SUMO3	CCNE1	LETM1	SF3B2
SAP30L	C20orf24	HAUS8	NUBP2	SUN1	CCT5	LGI1	SKIL
SLC25A3	C4orf46	HES1	NUP210	TBC1D9	CDK4	LMAN2	SLC25A39
SNHG3	C9orf103	HK2	PCMTD2	TCF25	CDKN1A	LPCAT1	SCL38A2
SNRPF	C4orf46	HMBS	PDZD11	TERF21P	CDKN1B	LRIG1	SCL7A5
SRSF3	C9orf103	HMGN2	PFKP	TFRC	CFL1	LSM14B	SLC9A3R1
TAF1C	CALM1	HMOX2	PFN1	TGIF2-C20orf24	CIAO1	LUC7L	SMAD7
TOR1A	CARHSP1	HNRNPH3	PGP	TIMM8B	CKS2	MARS	SMARCAD1
UCKL1	CBS	HNRNPL	PHGDH	TMBIM6	CLK1	MED13L	SNRPA
WNT5A	CCDC47	HNRNPM	PIM3	TMEM129	CLN5	MED22	SPSB3
	CCNE1	HNRNPUL2	PIN1	TMEM9	CLPTM1L	MOAP1	SQSTM1
	CCT5	HOX1A10-HOXA9	PKM2	TMPO	COG1	MRP63	STK25
	CDK16	HOXB8	PNN	TOR1B	COMTD1	MRPL38	SUMO3
	CDK4	HSBP1	POFUT2	TRIM11	CPNE1	MRPS34	TAF1C
	CFL1	ID2	POLD3	TRIP6	CSNK1D	MSL-1	TBC1D9
	CHERP	IDH3B	POLDIP2	TRMT2A	CSNK1E	MSX1	TCF25
	CHMP1A	ILF3	POLR2L	TTLL12	CTSD	MXD3	TFAP2A
	CHTOP	INPPL1	POP5	TTYH3	CUTA	MXD4	TFRC
	CIAO1	INSIG1	PPP1R15A	TUFM	DBN1	MYBBP1A	TMEM129
	CLN5	IQGAP3	PPP1R7	TYMS	DCXR	MYH10	TMPO
	CLPTM1L	IRX3	PPP2R1A	UBA1	DDIT4	MYH9	TOR1A
	COG1	KDELR2	PRDX1	UBB	DDX56	MYLIP	TPM1
	COMTD1	KDM3A	PRDX3	UBE2Q1	DERL1	NAPA	TRIM11
	COPS6	KDM5B	PREB	UBR7	DGCR8	NDUFA11	TRIP6
	COX4I1	KHSRP	PRMT1	UPF1	DHCR7	NDUFB10	TTLL12
	CPNE1	KIAA0141	PSAP	USP22	DNMT1	NME3	TTYH3
	CSNK1D	KIAA0664	PSMA7	VPS35	DYNLL1	NR2F2	TYMS
	CSNK1E	KRT8	PSMD6	WBSCR22	EEF2	NT5C	UBA1
	CST3	LETM1	PTBP1	WDR1	EIF3D	NT5DC2	UBE2Q1
	CTTN	LIG1	PTDSS2	WDR13	ELOVL1	NUBP2	UCKL1
	DBN1	LMAN2	PTMA	WDR6	ENSA	NUP210	UPF1
	DCAF8	LPCAT1	PURA	WDR75	EXOSC1	PAPOLA	USP16
	DCXR	LRIG1	RAC3	WFS1	FASN	PCMTD2	WSB1
	DDIT4	LRRC47	RBBP8	WSB1	FBXO21	PFKP	YWHAH
	DDOST	LSM14B	RBCK1	XBP1	FOSL2	PFN1	ZC3H14
	DDX39A	LUC7L	RBM28	YWHAH	FOXK1	PGRMC2	ZFP36L1
	DDX56	LZTR1	RBM3	ZC3H14	FOXP1	PHGDH	ZNF503
	DERL1	MAP7D3	RER1	ZFP36L1	FOXRED1	PIM3	ZNF527
	DGCR8	MARS	RHOB	ZMIZ2	FUS	PIN1	ZNF664
	DHCR7	MECOM	RNASEH2C	ZNF282	G6PD	PKM2	ZNF703
	DKFZP586I1420	MED13L	RNF167	ZNF503			
	DNMT1	MED22	ROMO1	ZNF527			
	DUSP3	MESDC2	RPL14	ZNF664			
	DYNLL1	MFSD10	RPL22	ZNF703			
	ECH1	MOAP1	RPL30				
	EEF2	MRP63	RPL36				
	EIF3D	MRPL38	RPN2				

Supplementary Table 1. List of BRD4, JMJD6, and CHD4 co-regulated genes that display increased DNA damage and R-loop accumulation following BRD4 inhibition. BRD4 regulated genes are listed in black. BRD4 and JMJD6 co-regulated genes are listed in red. BRD4 and CHD4 co-regulated genes are listed in blue.

Full Western Blots

Figure 1C

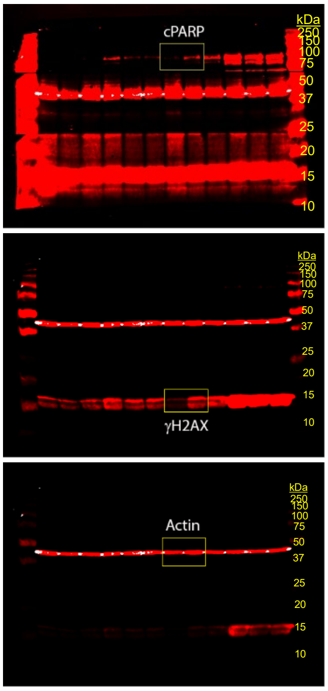

Figure 1E

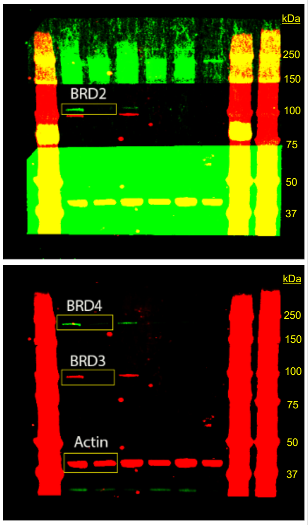

Figure 1H

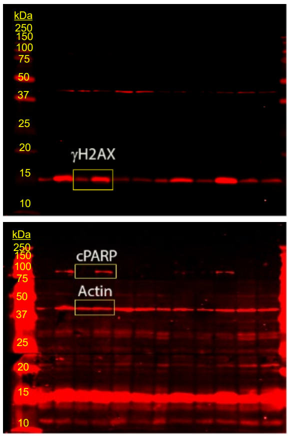

Figure 2B

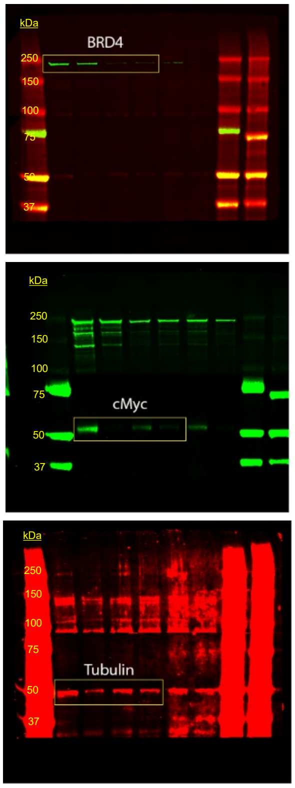

Figure 2D

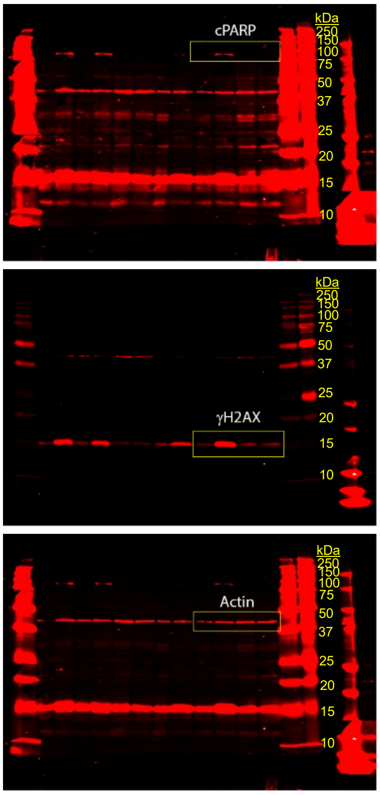

Figure 3D

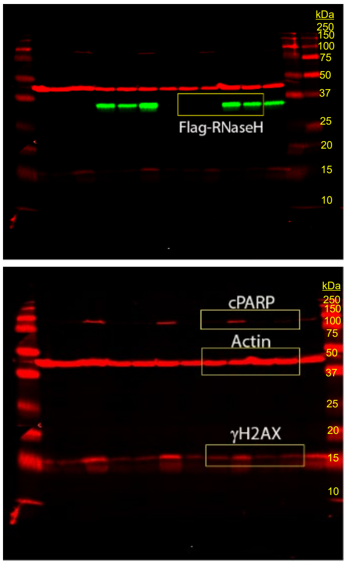

Figure 4A

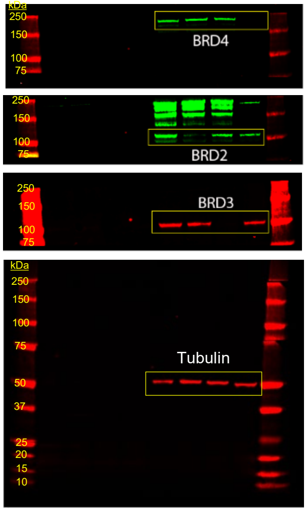

Figure 4B

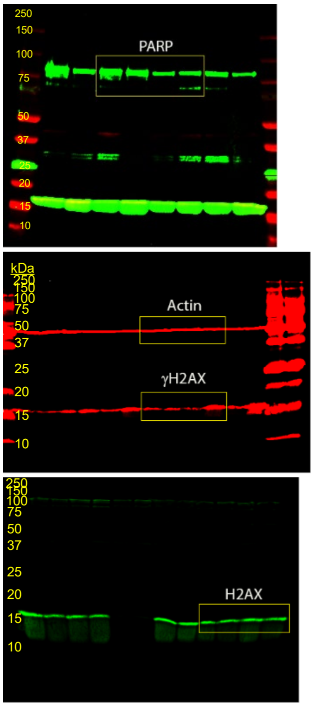

Figure 4D

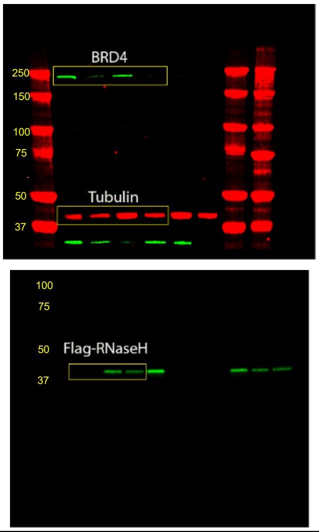

Figure 4F

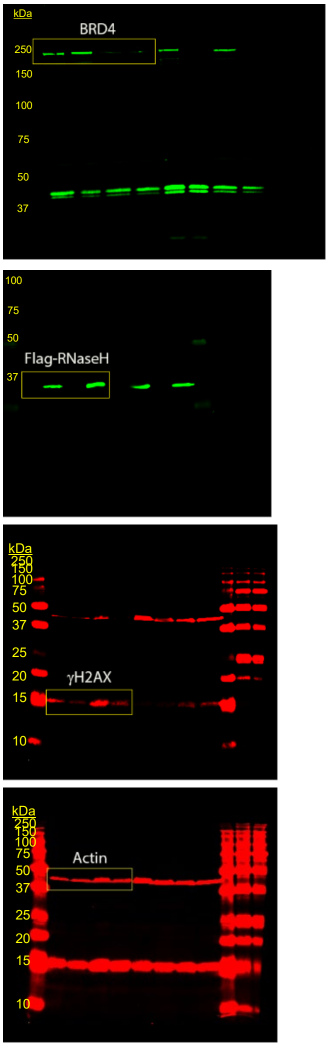

Figure 5A

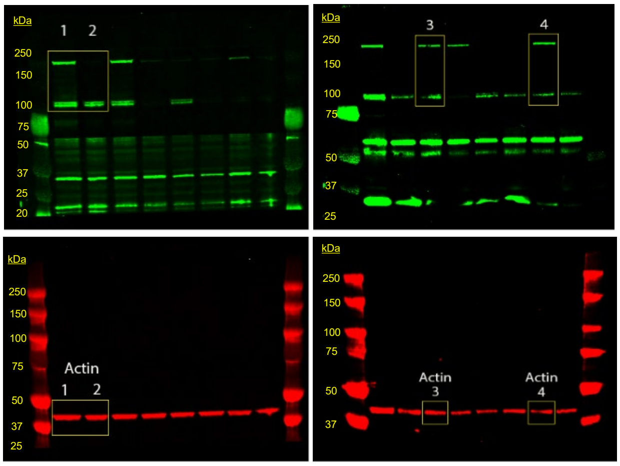

Figure 10C

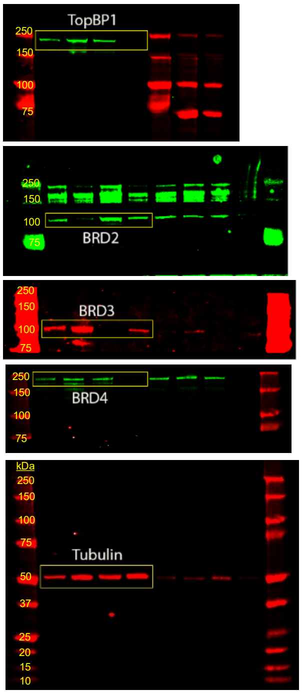

Figure 10D

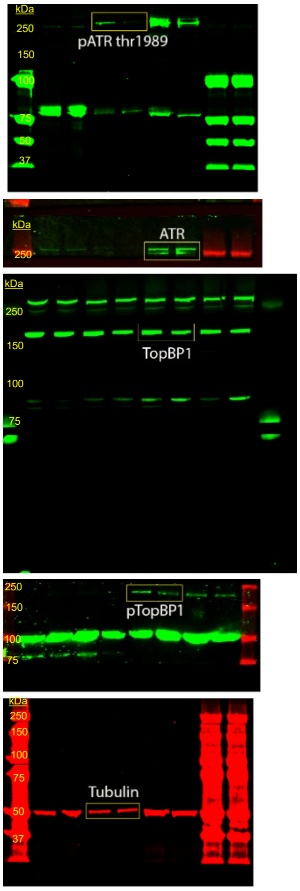

Figure 10E

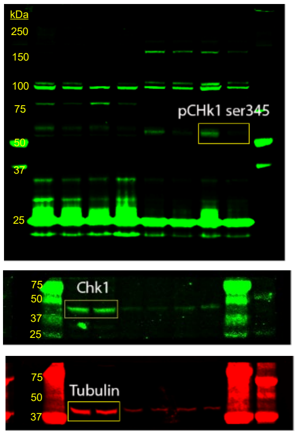

Suppl Figure 1C

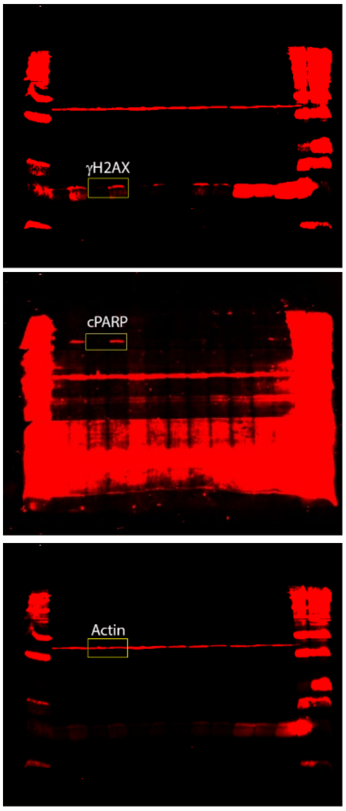

Suppl Figure 1E

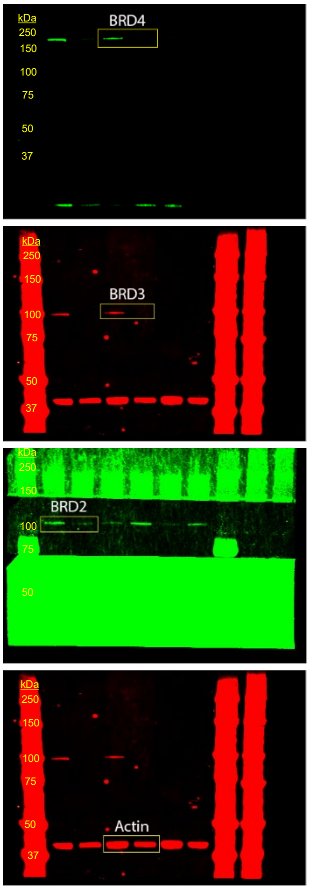

Suppl Figure 1H

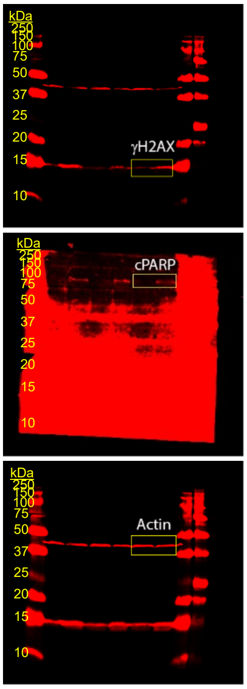

Suppl Figure 3D

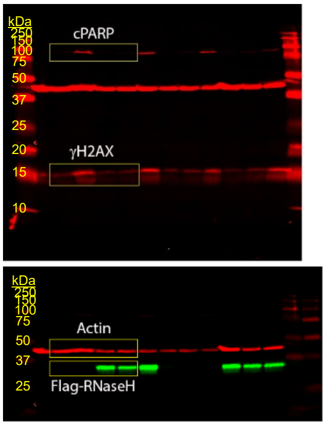

Suppl Figure 9A

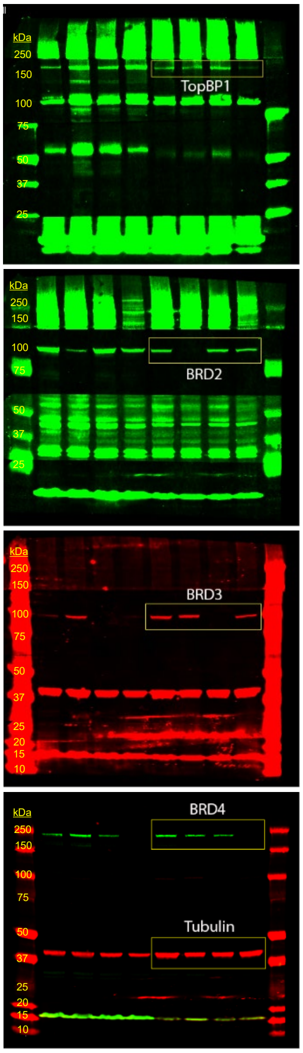

Suppl Figure 9C

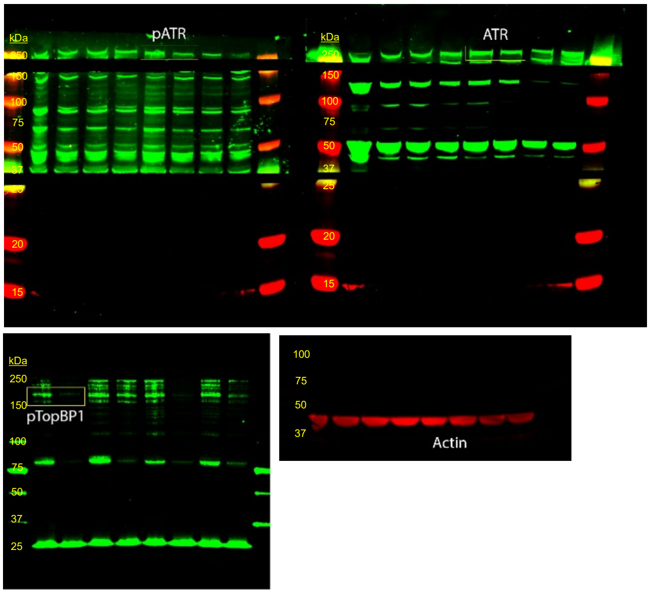

Suppl Figure 9D

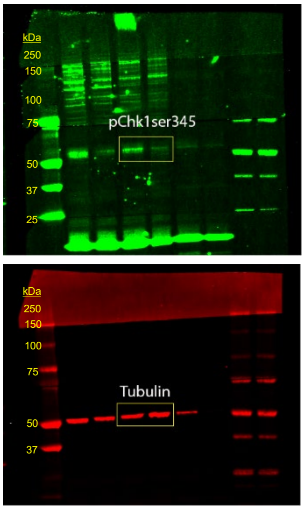

Suppl Figure 10B

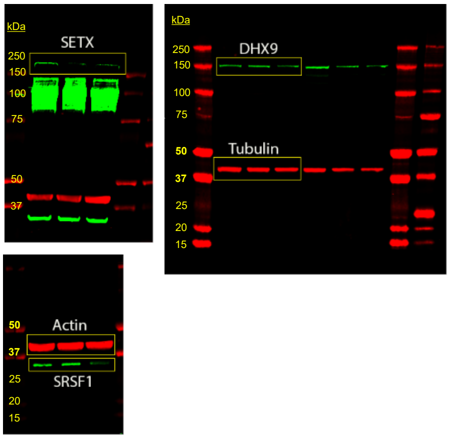

Suppl Figure 11A

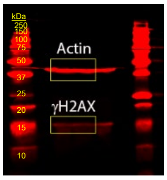

Supplement: Supplementary file 1 — Supplementary Information [file 41467_2020_17503_MOESM1_ESM.pdf]
