# Supplementary material for: BRD4 prevents the accumulation of R-loops and protects against transcription–replication collision events and DNA damage
Source: Nat Commun. 2020 Aug 14;11:4083. doi: 10.1038/s41467-020-17503-y (PMC7428008; doi:10.1038/s41467-020-17503-y)
Supplement: Supplementary file 3 — Reporting Summary [file 41467_2020_17503_MOESM3_ESM.pdf]

nature research

Corresponding author(s): Michael B Yaffe and Fred Lam

Last updated by author(s): June 15, 2020

Reporting Summary

Nature Research wishes to improve the reproducibility of the work that we publish. This form provides structure for consistency and transparency in reporting. For further information on Nature Research policies, see our Editorial Policies and the Editorial Policy Checklist.

Statistics

For all statistical analyses, confirm that the following items are present in the figure legend, table legend, main text, or Methods section.

n/a	Confirmed	
☐	☒	The exact sample size (*n*) for each experimental group/condition, given as a discrete number and unit of measurement
☐	☒	A statement on whether measurements were taken from distinct samples or whether the same sample was measured repeatedly
☐	☒	The statistical test(s) used AND whether they are one- or two-sided<br>*Only common tests should be described solely by name; describe more complex techniques in the Methods section.*
☒	☐	A description of all covariates tested
☒	☐	A description of any assumptions or corrections, such as tests of normality and adjustment for multiple comparisons
☐	☒	A full description of the statistical parameters including central tendency (e.g. means) or other basic estimates (e.g. regression coefficient) AND variation (e.g. standard deviation) or associated estimates of uncertainty (e.g. confidence intervals)
☐	☒	For null hypothesis testing, the test statistic (e.g. *F*, *t*, *r*) with confidence intervals, effect sizes, degrees of freedom and *P* value noted<br>*Give P values as exact values whenever suitable.*
☒	☐	For Bayesian analysis, information on the choice of priors and Markov chain Monte Carlo settings
☒	☐	For hierarchical and complex designs, identification of the appropriate level for tests and full reporting of outcomes
☒	☐	Estimates of effect sizes (e.g. Cohen's *d*, Pearson's *r*), indicating how they were calculated

Our web collection on statistics for biologists contains articles on many of the points above.

Software and code

Policy information about availability of computer code

Data collection

No software was used for data collection.

Data analysis

Paired-end ChIP-Seq sequencing data were mapped against the human genome hg19 assembly using the Burroughs-Wheeler Aligner BWA-MEM v. 0.7.12-r1039 with flag –t 4 and otherwise default parameters [bio-bwa.sourceforge.net].The resulting bam files were sorted and indexed using samtools v. 1.5 [http://www.htslib.org and LI2009], and duplicates were marked using Picard v. 2.9.0-1-gf5b9f50-SNAPSHOT (https://broadinstitute.github.io/picard/) MarkDuplicates with flags MAX_SEQUENCES_FOR_DISK_READ_ENDS_MAP=50000 MAX_FILE_HANDLES_FOR_READ_ENDS_MAP=8000 SORTING_COLLECTION_SIZE_RATIO=0.25 REMOVE_DUPLICATES=false ASSUME_SORTED=false DUPLICATE_SCORING_STRATEGY=SUM_OF_BASE_QUALITIES PROGRAM_RECORD_ID=MarkDuplicates PROGRAM_GROUP_NAME=MarkDuplicates OPTICAL_DUPLICATE_PIXEL_DISTANCE=100 VERBOSITY=INFO QUIET=false VALIDATION_STRINGENCY=SILENT COMPRESSION_LEVEL=5 MAX_RECORDS_IN_RAM=500000 CREATE_INDEX=false REMOVE_SEQUENCING_DUPLICATES=false TAGGING_POLICY=DontTag READ_NAME_REGEX=<optimized capture of last three ':' separated fields as numeric values> CREATE_MD5_FILE=false GA4GH_CLIENT_SECRETS=client_secrets.json . Bam files with duplicate reads marked were sorted and indexed again prior to being processed for downstream analyses.ChIP-Seq peaks were called using MACS2 v. 2.1.1.20160309 callpeak function with parameters –g hs –call-summits–p 1e-3 –nomodel –B with –ext matching the calculated insert size of each libraries , and using above-mentioned bam files from whole-cell extract and the chromatin-associated protein of interest as control and treatment, respectively. The resulting "narrowPeak" files were used for peak identification. In addition, wig files were prepared for each libraries using igvtool's count function, with –e matching the calculated insert size of the library and -w 25 , which were converted to BigWig using UCSC's wigToBigWig tool with default parameters and hg19's chromosome sizes as an input.

Coordinates of candidate genes were retrieved and expanded to a suitable distance 5' and 3' of the gene boundaries. Read densities were calculated as described in Marson200880, extending each read to 200 bps and tallying read counts over 25-bp bins tiling the regions of interest. Final read counts were normalized by each library's sequencing depth.

Genomic features were retrieved from the ENSEMBL GRCh37 v. 75 annotation of the hg19 genome assembly and reads were sequentially apportioned to transcription start sites (TSS) regions (defined as 50 bp upstream to 300 bp downstream of annotated TSSs), 5′ UTRs, transcription termination site regions (TTS, defined as 50 bp upstream and 200 bp downstream of annotated 3′ ends of transcripts), 3′UTR, exonic, intronic and intergenic regions using bedtools v. 2.26.0 intersect function.

Analysis of flow cytometry was performed using FlowJo v.X.

Analyses of statistics and plotting of graphs were performed using GraphPad Prism v.8.2.1.

Analysis of immunofluorescence micrographs and tail moments were performed on Fuji ImageJ v. 1.51n.

Analysis of tail moments was performed using the OpenComet plugin in ImageJ (http://www/opencomet.org)

For manuscripts utilizing custom algorithms or software that are central to the research but not yet described in published literature, software must be made available to editors and reviewers. We strongly encourage code deposition in a community repository (e.g. GitHub). See the Nature Research guidelines for submitting code & software for further information.

Data

Policy information about availability of data

All manuscripts must include a data availability statement. This statement should provide the following information, where applicable:

- Accession codes, unique identifiers, or web links for publicly available datasets
- A list of figures that have associated raw data
- A description of any restrictions on data availability

Data supporting the findings in this study are available within the article and the associated Supplementary Information Section. Any other data are available from the corresponding authors upon reasonable request.

Field-specific reporting

Please select the one below that is the best fit for your research. If you are not sure, read the appropriate sections before making your selection.

☒ Life sciences ☐ Behavioural & social sciences ☐ Ecological, evolutionary & environmental sciences

For a reference copy of the document with all sections, see nature.com/documents/nr-reporting-summary-flat.pdf

Life sciences study design

All studies must disclose on these points even when the disclosure is negative.

Sample size	For in vitro cell line experiments comparing drug effects on cellular protein levels using western blotting, each condition was performed in a 10 cm plate containing ~ 5 million cells at the beginning of the experiment. This sample size would provide more than ample total cellular lysates for loading of adequate amounts of protein for detection using validated antibodies.<br><br>For siRNA knockdown experiments, each 10 cm plate of cells treated with siRNA was approximately 50% confluent at the time of transfection, containing ~ 4 million cells per plate. This sample size would provide more than sufficient material (total protein or DNA) to perform western blotting or qPCR.<br><br>For ChIP seq and DRIP qPCR experiments, cells from 5 confluent 15 cm plates (~ 50 million cells) were pooled per drug condition per antibody used for ChIP or DRIP. This amount of cells would provide sufficient genomic DNA to perform ChIP or DRIP for downstream massive parallel sequencing or qPCR analyses.<br><br>For experiments using immunofluorescence,10k cells were plated onto 1 cm diameter glass cover slips and allowed to adhere for 24 hrs prior for use in experiments. This number of cells per cover slip is sufficient for imaging across multiple high power fields that contain at least 40 cells per statistical analysis.
Data exclusions	No data were excluded from the analyses.
Replication	All figures presented with error bars were derived from experiments that were successfully repeated in at least 3 biological replicates. Figures without error bars were repeated in 2 biological replicates, not because the third replication was unsuccessful but because we did not have the opportunity to repeat the experiment a third time.
Randomization	Experiments were randomized using randomly assigned plates of cells per condition or randomly plated and assigned cover slips of cells per condition. Vials of cells frozen down on different dates were randomly selected and thawed from liquid nitrogen stocks for biologic replicates. Drugs were randomly made fresh per experiment or pre-aliquoted frozen stocks of drugs were randomly selected for drugging of cells across replicates and different experiments.
Blinding	Samples were harvested and processed by technicians/scientists without knowledge of hypothesized treatment effects. ChIP seq samples DRIP qPCR samples were assigned generic labels (i.e. tube 1, tube 2, tube 3) and handed over for downstream processing by technicians/ scientists. Results were then properly labeled and assigned once data processing had been completed.

Reporting for specific materials, systems and methods

We require information from authors about some types of materials, experimental systems and methods used in many studies. Here, indicate whether each material, system or method listed is relevant to your study. If you are not sure if a list item applies to your research, read the appropriate section before selecting a response.

Materials & experimental systems

n/a	Involved in the study
☐	☒ Antibodies
☐	☒ Eukaryotic cell lines
☒	☐ Palaeontology and archaeology
☒	☐ Animals and other organisms
☒	☐ Human research participants
☒	☐ Clinical data
☐	☐ Dual use research of concern

Methods

n/a	Involved in the study
☐	☒ ChIP-seq
☐	☒ Flow cytometry
☒	☐ MRI-based neuroimaging

Antibodies

Antibodies used

Monoclonal antibodies against γH2AX were purchased from Millipore Sigma (Catalog No. 05-636) and Cell Signaling Technologies (Catalog No. 9718S); H2AX (Cell Signaling Technologies, Catalog No. 7631); actin (Sigma, Catalog No. A5441); cleaved PARP (BD Pharmigen, Catalog No. 552596); PARP (Cell Signaling Technologies, Catalog No. 9532S); cMyc (Cell Signaling Technologies, Catalog No. 5605S); BRD4 (Cell Signaling Technologies, Catalog No. 13440S); BRD2 (Cell Signaling Technologies, Catalog No. 5848S); BRD3 (Abcam, Catalog No. ab50818); tubulin (Sigma, Catalog No. T5168); FLAG peptide (Cell Signaling Technologies, Catalog No. 14793S); S9.6 (Kerafast, Catalog No. ENH001); RNAPII CTD repeat YSPTSPS phospho S2 (Abcam, Catalog No. ab5095); senataxin (Abcam, Catalog No. ab220827); DHX9 (ThermoFisher, Catalog No. PA5-19542); SRSF1 (Thermofisher, Catalog No. 32-4600); ATR (Cell Signaling Technology, Catalog No. 2790-S); phosphorylated ATR threonine 1989 (Genetex, Catalog No. GTX128145); phosphorylated RPA32 serine 33 (Bethyl, Catalog No. A300-246A); TopBP1 (Bethyl, Catalog No. A300-111A); phosphorylated TopBP1 serine 1138 (Raybiotech, Catalog No. 102-15561); phosphorylated Chk1 serine 345 (Cell Signaling Technology, Catalog No. 2348S); Chk1 (Cell Signaling Technology, Catalog No. 2360S). Pan-BRD4 polyclonal antibody was a gift from Cell Signaling Technologies (Clone PP12).

Validation

gH2AX - Millipore Sigma Cat No. 05-636. MQ600 Certified Validation Level. Immunoblot Analysis: 0.05-1 µg/ml of this antibody detected phosphorylated histone H2A.X (Ser139) in acid extracted histone lysates from Jurkat cells treated with 0.5 µM staurosporine (Catalog # 19-123). Immunocytochemistry: 2 µg/ml of this antibody detected phosphorylated histone H2A.X in HeLa cells treated with 0.5 µM staurosporine for 4-6 hours.
gH2AX - CST Cat No. 9718S. CST website antibody validation: 1) Western blot analysis of extracts from untreated or UV-treated 293 cells shows single band at expected MW of 15 kDa. 2) Immunohisto-chemical analysis of paraffin-embedded HT-29 cells untreated or UV-treated, shows increased nuclear staining in UV treated cells. Certificate of Analysis from CST website - https://media.cellsignal.com/coa/9718/17/9718-lot-17-coa.pdf.

H2AX – CST Cat No. 7631. CST website antibody validation: 1) Western blot analysis of extracts from various cell lines shows single band at expected MW of 15 kDa. 2) Immunohistochemical analyses of paraffin-embedded human breast, prostate carcinoma shows nuclear staining. Certificate of Analysis from CST website - https://media.cellsignal.com/coa/7631/6/7631-lot-6-coa.pdf.
Actin - Millipore Sigma Cat No. A5441. Monoclonal Anti-β-Actin (mouse IgG1 isotype) is derived from the AC-15 hybridoma produced by the fusion of mouse myeloma cells and splenocytes from an immunized mouse. Immunogen - A slightly modified β-cytoplasmic actin N-terminal peptide, Ac-Asp-Asp-Asp-Ile-Ala-Ala-Leu-Val-Ile-Asp-Asn-Gly-Ser-Gly-Lys, conjugated to KLH. Western blotting of HeLa, JURKAT, COS-7. NIH-3T3, PC-12, RAT2, CHO, MDBK, and MDCK cells shows single band of expected MW of 42 kDa. Certificate of Analysis from Millipore Sigma website - https://www.sigmaaldrich.com/Graphics/COfAInfo/SigmaSAPQM/COFA/A5/A5441/A5441-BULK_________079M4799V_.pdf.
Cleaved PARP - BD Pharmigen Cat No. 552596. Immunogen - A peptide corresponding to the N-terminus of the cleavage site (Asp 214) of human PARP was used as the immunogen. The F21-852 monoclonal antibody reacts only with the 89-kDa fragment of human PARP-1 that is downstream of the Caspase-3 cleavage site (Asp214) and contains the automodification and catalytic domains. It does not react with intact human PARP-1. Western blotting of JURKAT cells untreated or treated with camptothecin 4 uM for 4 hrs to induce apoptosis shows a single band at expected MW of ~ 89 kDa.

PARP – CST Cat No. 9532S. CST website antibody validation: 1) Western blot analysis of extracts from control HEK293 cells or PARP knockout HEK293 cells shows the absence of signal in the PARP knockout HEK293 cells of a single band of expected MW of 116 kDa, confirming the specificity of the antibody for PARP. 2) Western blot analysis of extracts from THP-1 cells, untreated or treated with TNF-α and cycloheximide as well as control extracts from SW620 and A20 cell lines shows a single band of full-length PARP at the expected MW of 116 kDa and single band of cleaved PARP at the expected MW of 89 kDa. Certificate of Analysis from CST website - https://media.cellsignal.com/coa/9532/9/9532-lot-9-coa.pdf.
cMyc – CST Cat No. 5605S. CST website antibody validation: Western blot analysis of extracts from control HEK293 cells or c-Myc knockout HEK293 cells. The absence of signal in the c-Myc knockout HEK293 cells of a single band at the expected MW of ~57-65 kDa confirms specificity of the antibody for c-Myc. Certificate of Analysis from CST website - https://media.cellsignal.com/coa/5605/15/5605-lot-15-coa.pdf.

BRD4 – CST Cat No. 13440S. CST website antibody validation: 1) Western blot analysis of extracts from RL-7, 293T, and Jurkat cells shows single band of expected MW of ~ 200 kDa. 2) Immunoprecipitation of BRD4 from RL-7 cell extracts using Rabbit (DA1E) mAb IgG XP® Isotype Control or BRD4 (E2A7X) Rabbit mAb shows a single band at the expected MW of ~ 200 kDa in the BRD4 IP lane and absence of a band in the Isotype Control lane confirming specificity of the antibody for BRD4. Lane with 10% input shows single band

at the expected MW of ~ 200 kDa. Certificate of Analysis from CST website - https://media.cellsignal.com/coa/13440/6/13440-lot-6-coa.pdf.
BRD2 – CST Cat No. 5848S. CST website antibody validation: 1) Western blot analysis of extracts from MOLT-4 and NCCIT cells shows single band of expected MW of 110 kDa. 2) Chromatin immunoprecipitations were performed with cross-linked chromatin from NCCIT cells and either Brd2 (D89B4) Rabbit mAb or Normal Rabbit IgG #2729 using SimpleChIP® Plus Enzymatic Chromatin IP Kit (Magnetic Beads) #9005. The enriched DNA was quantified by real-time PCR using human GTF3C6 exon 1 primers, SimpleChIP® Human SF3B3 Exon 1 Primers #62858, and SimpleChIP® Human MyoD1 Exon 1 Primers #4490. The amount of immunoprecipitated DNA in each sample is represented as signal relative to the total amount of input chromatin, which is equivalent to one. Brd2 ChIP signal shows relative enriched signal compared to Rabbit IgG showing specficity of the antibody for Brd2 at known Brd2 target genes. 3) Chromatin immunoprecipitations followed by parallel sequencing (ChIP-seq) were performed with cross-linked chromatin from NCCIT cells and Brd2 (D89B4) Rabbit mAb, using SimpleChIP® Plus Enzymatic Chromatin IP Kit (Magnetic Beads) #9005. DNA Libraries were prepared using SimpleChIP® ChIP-seq DNA Library Prep Kit for Illumina® #56795. The figure shows binding across GTF3C6, a known target gene of BRD2 (see additional figure containing ChIP-qPCR data). For additional ChIP-seq tracks, please download the product data sheet. Certificate of Analysis from CST website - https://media.cellsignal.com/coa/5848/3/5848-lot-3-coa.pdf.

BRD3 – Abcam Cat No. ab50818. Abcam website antibody validation: 1) Western blot analysis of Anti-BRD3 antibody [2088C3a] (ab50818) at 1/200 dilution using F2408 whole cell lysate at 50 µg shows single band of expected MW ~ 80 kDa. 2) Western blot analysis of Anti-BRD3 antibody [2088C3a] (ab50818) at 1/50 dilution using HeLa whole cell lysate at 50 µg shows single band of expected MW ~ 80 kDa. "
Tubulin – Sigma Cat No. T5168. Sigma website antibody validation: 1) Monoclonal Anti-α-Tubulin (mouse IgG1 isotype) is derived from the B-5-1-2 hybridoma produced by the fusion of mouse myeloma cells and splenocytes from an immunized mouse. 2) Recognizes an epitope located at the C-terminal end of the α-tubulin isoform in a variety of organisms. 3) Immunogen: Sarkosyl-resistant filaments from Strongylocentrotus purpuratus (sea urchin) sperm axonemes. 4) Western blot of HeLa, JURKAT, COS7, NIH-3T3, PC-12, RAT2, CHO, MDBK, and MDCK cell lysates shows a single band of expected MW ~ 47 kDa.

FLAG (DYKDDDDK) tag – CST Cat No. 14793S. CST website antibody validation: 1) Western blot analysis of extracts from 293T cells, mock transfected (-) or transfected with DYKDDDDK-GFP (N-terminal DDK-Tag; +), GFP-DYKDDDDK (C-terminal DDK-Tag; +), human CASQ1-DYKDDDDK (C-terminal DDK-Tag; +), or human FoxG1-DYKDDDDK (C-terminal DDK-Tag; +) as indicated, using DYKDDDDK Tag (D6W5B) Rabbit mAb. Certificate of Analysis from CST website - https://media.cellsignal.com/coa/14793/5/14793-lot-5-coa.pdf.
S9.6 – Kerafast Cat No. ENH001. Kerafast website antibody validation: 1) This mouse monoclonal antibody was generated against a ΦX174 bacteriophage-derived synthetic DNA–RNA antigen and recognizes RNA-DNA hybrids of various lengths. 2) Chromatin Immunoprecipitation (ChIP) Analysis: A representative lot detected increased DNA RNA hybrids at four actively transcribed genes upon shRNA-mediated knockdown of BRCA1 or BRCA2, but not PCID2 or RAD51 in HeLa cells (Bhatia, V., et al. (2014). Nature. 511(7509):362-365). 3) Chromatin Immunoprecipitation (ChIP) Analysis: A representative lot detected R-loops formed over beta-actin gene using HeLa chromatin preparation. RNase H treatment of the chromatin preparation prevented clone S9.6 from immunoprecipitating target chromatin fragments (Skourti-Stathaki, K., et al. (2011). Mol. Cell. 42(6):794-805). 4) Immunocytochemistry Analysis: Representative lots immunolocalized nuclear R loops by fluorescent immunocytochemistry staining of methanol-fixed H1 human embryonic stem cells (hESCs) and formaldehyde-fixed HeLa cells (Bhatia, V., et al. (2014). Nature. 511(7509):362-365; Ginno, P.A., et al. (2012). Mol. Cell. 45(6):814-825). 5) Immunoprecipitation Analysis: A representative lot immunoprecipitated in vitro transcribed R-loop substrate (DNA-RNA hybrid), but not doouble-stranded DNA (dsDNA) (Ginno, P.A., et al. (2012). Mol. Cell. 45(6):814-825). Antibody developed from the laboratory of Stephen H. Leppla, PhD, National Institute of Allergy and Infectious Diseases/NIH.

RNAPII CTD YSPTSPS phospho S2 – Abcam Cat No. ab5095. Abcam website antibody validation: 1) This antibody recognises the phosphorylated serine found in the amino acid 2 position of the C-terminal domain repeat YSPTSPS. 2) Immunogen - synthetic peptide corresponding to Saccharomyces cerevisiae RNA polymerase II CTD repeat YSPTSPS aa 1600-1700 conjugated to keyhole limpet haemocyanin. Database link: P04050 3) This antibody recognises the phosphorylated serine found in the amino acid 2 position of the C-terminal domain repeat YSPTSPS. 4) ChIP of RAW macrophages expressing c-fms locus (MCSF receptor gene locus) compared to 3T3 fibroblasts (not expressing c-fms) shows enrichment at upstream control, promoter, intronic enhancer, and exon 3 in RAW macrophages but not 3T3 fibroblasts. 5) Western blot of HeLa, S. cerevisiae lysates shows single band at expected MW ~ 217 kDa.
Senataxin – Abcam Cat No. ab243904. Abcam website antibody validation: 1) Immunogen - Synthetic peptide within Human Senataxin aa 900-950. The exact sequence is proprietary. NP_055861.3 and Gene ID 23064. 2) Western blot of immunoprecipitate of senataxin immunoprecipitated from 1.0 mg of HeLa whole cell lysate using the antibody shows a single band of expected MW at ~ 270 kDa. 3) Western blot of HeLa, HEK293T, MCF7, HepG2, A549, SW 620, SK-MEL-28, JURKAT, K562, RPMI 8226, and 786-O whole cell lysates shows a single band of expected MW at ~ 270 kDa.
DHX9 – ThermoFisher Cat No. PA5-19542. ThermoFisher website antibody validation: 1) Western blot of A-431 cells transfected with DHX9 siRNA showing reduction in signal of a single band at expected MW of ~ 149 kDa compared to cells that were transfected with scramble siRNA and untransfected cells. 2) Western blot of HeLa, HEK-293, NIH-3T3, and A-431 whole cell lysates show a single band at expected MW of ~ 149 kDa.

SRSF1 – ThermoFisher Cat No. 32-4600. ThermoFisher website antibody validation: 1) Western blot of Caco-2, HEK-293, HeLa, MCF7, A-431, A-549, NIH-3T3 shows single band at expected MW ~ 32 kDa 2) Immunogen - recombinant SF2/ASF protein.
ATR – CST Cat No. 2790S. CST website antibody validation: 1) Western blot of HeLa and HT-29 whole cell lysates show single band at expected MW ~ 300 kDa. 2) Polyclonal antibodies are produced by immunizing animals with a synthetic peptide corresponding to central residues of human ATR. Antibodies are purified by protein A and peptide affinity chromatography. Certificate of Analysis from CST website - https://media.cellsignal.com/coa/2790/9/2790-lot-9-coa.pdf.
phospho ATR thr1989 – Genetex Cat No. GTX128145. GeneTex website antibody validation: 1) Western blot of Raji and Raji nuclear extracts shows single band at expected MW ~ 280 kDa. 2) Immunohisto-chemistry of human colon and breast cancers show nuclear staining. 3) Immunoprecipitation of ATR (phospho Thr1989) protein from HCT-116 whole cell extracts treated with HU for 6 hrs shows single band at expected MW ~ 300 kDa.

phospho RPA32 serine 33 – Bethyl Cat No. A300-246A. Bethyl website antibody validation: 1) Western blot of JURKAT cells mock treated or etoposide treated cells shows single band at expected MW ~ 33 kDA in etoposide treated cells. 2) Immunogen - surrounding serine 33 of RPA32.
TopBP1 – Bethyl Cat No. A300-111A. Bethyl website antibody validation: 1) Western blot of HeLa, HEK293T, and JURKAT whole cell lysates shows single band at expected MW ~ 200 kDa. 2) Western blot of immunoprecipitation of HeLa whole cell lysates using anti-

TopBP1 antibody but not Control IgG shows single band at expected MW ~ 200 kDa. 3) Immunogen - between aa 1400 and C-terminus of TopBP1.

phospho TopBP1 ser 1138 – Raybiotech Cat No. 102-15561. Raybiotech website antibody validation: 1) Dot blot analysis of 50 ng of phospho-peptide or non-phosphopeptide blotted with 0.6 ug per mL of anti-TopBP1 S1138 shows a dot signal with the phospho-peptide. 2) This TOPBP1 Antibody is generated from rabbits immunized with a KLH conjugated synthetic phosphopeptide corresponding to amino acid residues surrounding S1138 of human TOPBP1.
phospho Chk1 ser 345 – CST Cat No. 2348S. CST website antibody validation: 1) Western blot analysis of whole cell lysates of HeLa, COS, NIH-3T3, and C6 cells untreated or UV-treated shows single band at expected MW ~ 56 kDa. 2) Monoclonal antibody is produced by immunizing animals with a synthetic phosphopeptide corresponding to residues surrounding Ser345 of human Chk1. Certificate of Analysis - https://media.cellsignal.com/coa/2348/18/2348-lot-18-coa.pdf.

Chk1 – CST Cat No. 2360S. CST website antibody validation: 1) Western blot analysis of whole cell lysates of A431, HeLa, and NIH-3T3 cells shows single band at expected MW ~ 56 kDa. 2) Western blot analysis of HeLa cell extracts transfected with Control siRNA or 2 different Chk1 siRNAs shows reduction in signal in cells transfected with Chk1 siRNAs.

Pan BRD4 Polyclonal Antibody (Clone PP12) – CST, gift from CST Testing lab. CST testing lab has performed Western blot analysis of lysates of HEK293 cells transiently overexpressing Brd4 isoforms A, B, or C. The PP12 clone detected bands corresponding to the expected MW of the different isoforms of Brd4 (Isoform A ~ 200 kDa; Isoform B ~ 180 kDa; and Isoform C ~ 100 kDa). We designed siRNAs targeting the different isoforms of Brd4 and have shown reduced signal in HeLa cell lysates on western blot of the appropriate sized bands corresponding to Isoform A, B, and C (see Figure 5A of manuscript).

Eukaryotic cell lines

Policy information about cell lines

Cell line source(s)	HeLa and HCT116 cells were purchased through ATCC. RNase H1-inducible HeLa cells were a gift from Karlene Cimprich's Lab at Stanford University.
Authentication	None of the cell lines used were authenticated.
Mycoplasma contamination	Cell lines were tested regularly for mycoplasma contamination and were all confirmed negative for contamination.
Commonly misidentified lines (See ICLAC register)	No commonly misidentified cell lines were used in this study.

Dual use research of concern

Policy information about dual use research of concern

Hazards

Could the accidental, deliberate or reckless misuse of agents or technologies generated in the work, or the application of information presented in the manuscript, pose a threat to:

No	Yes	
☒	☐	Public health
☒	☐	National security
☒	☐	Crops and/or livestock
☒	☐	Ecosystems
☒	☐	Any other significant area

Experiments of concern

Does the work involve any of these experiments of concern:

No	Yes	
☒	☐	Demonstrate how to render a vaccine ineffective
☒	☐	Confer resistance to therapeutically useful antibiotics or antiviral agents
☒	☐	Enhance the virulence of a pathogen or render a nonpathogen virulent
☒	☐	Increase transmissibility of a pathogen
☒	☐	Alter the host range of a pathogen
☒	☐	Enable evasion of diagnostic/detection modalities
☒	☐	Enable the weaponization of a biological agent or toxin
☒	☐	Any other potentially harmful combination of experiments and agents

ChIP-seq

Data deposition

☒ Confirm that both raw and final processed data have been deposited in a public database such as GEO.

☒ Confirm that you have deposited or provided access to graph files (e.g. BED files) for the called peaks.

Data access links<br>*May remain private before publication.*	https://www.ncbi.nlm.nih.gov/geo/query/acc.cgi?acc=GSE151038<br>Token for access: ojirqesevjupzir<br><br>(Accession number GSE151038)
Files in database submission	GSM4564682: DMSO Input, GSM4564683: DMSO IgG ChIP-Seq, GSM4564684: DMSO BRD4 ChIP-Seq, GSM4564685: DMSO gH2AX ChIP-Seq, GSM4564686: DMSO RNApolII ser2 ChIP-Seq, GSM4564687: JQ1 InputGSM4564688, JQ1 IgG ChIP-Seq, GSM4564689: JQ1 BRD4 ChIP-Seq, GSM4564690: JQ1 gH2AX ChIP-Seq, GSM4564691: JQ1 RNApolII ser2 ChIP-Seq
Genome browser session<br>(e.g. UCSC)	https://genome.ucsc.edu/cgi-bin/hgTracks?db=hg19&lastVirtModeType=default&lastVirtModeExtraState=&virtModeType=default&virtMode=0&nonVirtPosition=&position=chr7%3A155089486%2D155101945&hgsid=836900253_aAgaPETbPVomcoyV6PbwXnzFRSFE

Methodology

Replicates	We performed 2 biological replicates.
Sequencing depth	We performed 75 nt paired-end reads at 40 million read depth per sample on an Illumina NextSeq 500.
Antibodies	BRD4 antibody (Cell Signaling Technologies, Catalog No. 13440S), gH2AX (Millipore Sigma, Catalog No. 05-636), or RNAPII CTD repeat YSPTSPS phospho S2 (Abcam, Catalog No. ab5095). All ChIP antibodies were validated ChIP grade as per the manufacturer. Normal rabbit IgG (EMD Millipore, Catalog No. 12-370.)
Peak calling parameters	ChIP-Seq peaks were called using MACS2 v. 2.1.1.20160309 callpeak function with parameters –g hs –call-summits–p 1e-3 –nomodel –B with –ext matching the calculated insert size of each libraries , and using above-mentioned bam files from whole-cell extract and the chromatin-associated protein of interest as control and treatment, respectively. The resulting “narrowPeak” files were used for peak identification. In addition, wig files were prepared for each libraries using igvtool’s count function, with –e matching the calculated insert size of the library and -w 25 , which were converted to BigWig using UCSC’s wigToBigWig tool with default parameters and hg19’s chromosome sizes as an input.
Data quality	Paired-end ChIP-Seq sequencing data were mapped against the human genome hg19 assembly using the Burroughs-Wheeler Anigner BWA-MEM v. 0.7.12-r1039 with flag –t 4 and otherwise default parameters [bio-bwa.sourceforge.net]77. The resulting bam files were sorted and indexed using samtools v. 1.5 [http://www.htslib.org and LI2009], and duplicates were marked using Picard v. 2.9.0-1-gf5b9f50-SNAPSHOT (https://broadinstitute.github.io/picard/) MarkDuplicates with flags MAX_SEQUENCES_FOR_DISK_READ_ENDS_MAP=50000 MAX_FILE_HANDLES_FOR_READ_ENDS_MAP=8000 SORTING_COLLECTION_SIZE_RATIO=0.25 REMOVE_DUPLICATES=false ASSUME_SORTED=false DUPLICATE_SCORING_STRATEGY=SUM_OF_BASE_QUALITIES PROGRAM_RECORD_ID=MarkDuplicates PROGRAM_GROUP_NAME=MarkDuplicates OPTICAL_DUPLICATE_PIXEL_DISTANCE=100 VERBOSITY=INFO QUIET=false VALIDATION_STRINGENCY=SILENT COMPRESSION_LEVEL=5 MAX_RECORDS_IN_RAM=500000 CREATE_INDEX=false REMOVE_SEQUENCING_DUPLICATES=false TAGGING_POLICY=DontTag READ_NAME_REGEX=<optimized capture of last three ':' separated fields as numeric values> CREATE_MD5_FILE=false GA4GH_CLIENT_SECRETS=client_secrets.json . Bam files with duplicate reads marked were sorted and indexed again prior to being processed for downstream analyses.
Software	Paired-end ChIP-Seq sequencing data were mapped against the human genome hg19 assembly using the Burroughs-Wheeler Anigner BWA-MEM v. 0.7.12-r1039 with flag –t 4 and otherwise default parameters [bio-bwa.sourceforge.net]77. The resulting bam files were sorted and indexed using samtools v. 1.5 [http://www.htslib.org and LI2009], and duplicates were marked using Picard v. 2.9.0-1-gf5b9f50-SNAPSHOT (https://broadinstitute.github.io/picard/).<br><br>ChIP-Seq peaks were called using MACS2 v. 2.1.1.20160309 callpeak function with parameters –g hs –call-summits–p 1e-3 –nomodel –B with –ext matching the calculated insert size of each libraries , and using above-mentioned bam files from whole-cell extract and the chromatin-associated protein of interest as control and treatment, respectively. The resulting “narrowPeak” files were used for peak identification. In addition, wig files were prepared for each libraries using igvtool’s count function, with –e matching the calculated insert size of the library and -w 25 , which were converted to BigWig using UCSC’s wigToBigWig tool with default parameters and hg19’s chromosome sizes as an input.

Flow Cytometry

Plots

Confirm that:

☒ The axis labels state the marker and fluorochrome used (e.g. CD4-FITC).

☒ The axis scales are clearly visible. Include numbers along axes only for bottom left plot of group (a 'group' is an analysis of identical markers).

☒ All plots are contour plots with outliers or pseudocolor plots.

☒ A numerical value for number of cells or percentage (with statistics) is provided.

Methodology

Sample preparation	For cell cycle studies, HeLa, HCT116, and RNase H-inducible HeLa cells were treated with DMSO or 500 nM JQ1 for 48 hrs, harvested, fixed, and stained using DAPI for DNA content.
Instrument	FACS LSR II HTS-1 flow cytometer (BD Biosciences).
Software	Flow data was analyzed using FlowJo software.
Cell population abundance	Ten thousand events were recorded per sample on a FACS LSR II HTS-1 flow cytometer. Greater than 90% of events were selected using the SSC vs FSC plots centered around the most centrally dense clustering of cells.
Gating strategy	To obtain DAPI content for cell cycle plots, no further gating was performed once greater than 90% of events were selected using the SSC vs FSC plots. DNA content histograms were obtained using the DAPI setting on the X-axis in the FloJo software. A representative figure of gating strategies is proved in the Supplementary Information (Supplementary Figure 12).

☒ Tick this box to confirm that a figure exemplifying the gating strategy is provided in the Supplementary Information.
