# Supplementary material for: BRD4 prevents the accumulation of R-loops and protects against transcription–replication collision events and DNA damage
Source: Nat Commun. 2020 Aug 14;11:4083. doi: 10.1038/s41467-020-17503-y (PMC7428008; doi:10.1038/s41467-020-17503-y)
Supplement: Supplementary file 2 — Peer Review [file 41467_2020_17503_MOESM2_ESM.pdf]

Reviewers' comments:

Reviewer #1 (Remarks to the Author):

This manuscript provides new insights into how DSBs are induced by inhibition of BET family proteins including BRD4. Previously, these authors demonstrated that a variant of BRD4 protected chromatin from DNA damage response by insulating chromatin from DNA damage signaling. The source of this damage upon inhibition of BET family was not clear. Here, this manuscript tries to address this question by showning that BRD4 inhibition induces transcription-replication conflicts as a result of R-loop formation. This suggests that the BET family proteins are important for the correct loading of transcription co-activator complexes such as pTEFb to regulate transcription and avoid R-loop formation. The observation that R-loop formation by BRD4 inhibition is novel and builds upon this groups previous work showing that inhibition of BRD4 induced DNA damage responses. Overall this work is interesting but there are still several issues that need to be addressed before publication.

Major comment:

1. Inhibition of BRD4 induced transcription-replication conflicts in S phase. In this state, BRD4 knock down cells are likely to be arrested in S and G2 phase. However, in these groups previous studies, BRD4 knockdown cells do not display any cell cycle defects (Scott R. Floyd et al., Nature, 2013). In addition, JQ1 treatment also did not induce phospho-H2AX (Figure 4e of this paper). One possibility is that this phenomenon occurs in a sub-set of cancer cells and its mechanisms are not universal. These data seem at odds and need to be explained.

2. Given that the authors claim that these effects occur in S-phase, cell cycle analyses for all key experiments need to be performed. This includes Triptolide and RNase H1 o/e analyses. In addition, growth curves -/+ JQ1 with and without RNase H1 should be performed to see how these treatments effect cell proliferation and not just the phenotypic readouts of DNA damage.

3. The data in figure 5B, in the quantification graph show that BRD4 knock down cells only increased phospho-H2AX level but in WB image, BRD3 knockdown cells also increased phospho-H2AX even if the actin level was less than control and BRD4 knockdown cells. The graph and WB image seem inconsistent. Given the poor quality of these western blots, new data should be provided and for these experiments unmodified H2AX should be used and total PARP staining should be analyzed.

4. There are three isoform of BRD4 in the cells (Scott R. Floyd et al., Nature, 2013). Which BRD4 isoform affects R-loop formation?

5. In this paper, BRD4 inhibition induces DNA damage and it would be interesting to know if this relates with deregulated transcription. Is it dependent on dysfunction of pTEFb on transcription? One might think that BRD4 inhibition should reduce transcription. Similarly, Triptolide also inhibits transcription but has opposite effects. This should be explained.

6. Based on these data, it is curious that BRD4 isoform A knock down in cells does not increase phospho-H2AX levels in undamaged cells ((Scott R. Floyd et al., Nature, 2013)? BRD4 isoform A contain the CTM domain, which is critical for interaction with pTEFb. Does BRD4 CTM mutant show the same phenotype as BRD4 knock down?

7. If R-loop formation induces DNA damage, why do the foci of S9.6 not co-localize with DNA damage markers. This is a major issue that must be fully addressed. One possibility is that the phospho-H2AX seen is a result from apoptosis and not R-loops. All the data shown shows that phospho-H2AX and apoptosis are occurring. Perhaps altering treatment conditions could provide a scenario where DNA

damage is induced but not enough to provoke apoptosis. One could also block apoptosis and show that DNA damage still occurs.

Minor comment:

1. In the figure1, panel C, phospho-H2AX should be white bar not black.

2. In page 6, 2nd low, DBS formation should be DSB formation.

Reviewer #2 (Remarks to the Author):

In this manuscript, the authors uncover a previously unappreciated function of BET bromodomain proteins, particularly BRD4, in the regulation transcription-replication conflicts by preventing R-loop formation. They show that inhibition of BET bromodomain proteins leads to increased DSB formation and apoptosis in different cancer cell lines. The authors use a specific transcription initiation inhibitor (triptolide) to show that the DNA damage and apoptotic phenotype observed with BET bromodomain inhibitors requires active transcription. Next, they propose that this damage originates primarily as a consequence of replication-transcription conflicts because it occurs primarily within the S-phase. Finally, they conclude that DNA damage is due to the accumulation of R-loops by combining experiments with the S9.6 antibody that specifically detects R-loops and RNase H1 that suppresses R-loop formation. Lastly, they selectively knockdown distinct BET bromodomain proteins by siRNA and suggest that BRD4 is primarily responsible for the observed phenotype. Collectively, this is an interesting study that points to a novel function of BET bromodomain proteins in regulating R-loop formation and replication-transcription conflicts. The studies are however too preliminary and have not been developed to the point that it should be considered for publication in a high-profile journal.

Major concerns:

1. The authors’ statement that BET bromodomain proteins are important to prevent R-loop formation is not supported by the data. The authors cannot distinguish whether loss/inhibition of BET bromodomain proteins leads to increased R-loop accumulation because these proteins are needed to prevent R-loop formation, as the authors suggest, or to favor R-loop resolution. The authors should include further experiments to distinguish between these two alternative possibilities.

2. The authors should include more experiments to provide mechanistic insights into the actual role of BRD4 in R-loop regulation.

3. The authors should explore the role of the previously reported interactions of BRD4 with the positive elongation factor (P-TEFb) and the transcriptional co-activators JMJD6 and CHD4 in regulating transcription-replication conflicts and R-loop formation.

4. The authors should include additional experiments to strengthen their conclusion that the DNA damage observed with BET bromodomain inhibitors originates from transcription-replication conflicts. For example, they could use single-molecule DNA fiber assays to directly monitor replication perturbations and demonstrate that replication is impaired when cells are treated with the with BET bromodomain inhibitors.

5. The authors should include a quantification for the EdU incorporation experiments shown in Figure 2A.

6. Figure 3. The authors should measure the percentage of S-phase cells. On the basis of the authors' model that R-loop accumulation perturbs replication progression, they might find an increased percentage of S-phase cells following treatment with BET bromodomain inhibitors, due to S-phase arrest. Along the same line, they might find a decreased EdU incorporation because of the increased fork stalling associated with R-loop accumulation.

Reviewer #3 (Remarks to the Author):

This article describes the discovery that BRD4 inhibition leads to transcription-coupled DNA damage. This damage is at least partly driven by the accumulation of R-loops which the authors speculate is due to transcriptional dysregulation. Overall the story presented is very straightforward and the authors have been rigorous in applying multiple reagents to inhibit or deplete BRD proteins and show their results are consistent in different cell lines. The flip side of this is that the story can be viewed as quite superficial from a mechanistic perspective, and the authors themselves point out that the mechanism of R-loop induced DNA damage in BRD4-depleted cells remains unknown, even concluding the discussion with a suggested experiment: DRIP-sequencing of BRD4-depleted cells.

The work is solid and supports the conclusions, but is at an observational stage, rather than a mechanistic stage. The observations are interesting and below I make suggestions for improvements.

1. The S9.6 antibody has now been used repeatedly for precipitation of R-loops. I suggest that DRIP experiments, followed by qPCR at known BRD4-target loci, (or by deep sequencing if feasible), would enhance the work. It would also allow some direct tests of mechanisms. For example, to show that BRD4 ChIPs to the same locus, then upon depletion, R-loops accumulate at that locus. This would at least implicate a direct model of R-loop induction rather than other indirect effects on gene expression networks.

2. The article uses comet assays and total g-H2AX staining intensity throughout to monitor DNA damage. While they do correlate the damage with S-phase, exploration of the damage response is quite limited. The authors might consider native BrdU immunofluorescence, or monitoring RPA2-S33 phosphorylation, and probing the ATR signaling pathway to really establish that DNA replication stress is arising from the BRD protein depletions.

3. I found it unfortunate that chemical inhibitors with multiple targets were used for all experiments except those in Figure 5 where siRNA was used to specifically implicate BRD4. It would be nice to at least repeat the triptolide experiments in the BRD4-siRNA setting, or probe the mechanism more deeply with the BRD4-siRNA reagents which would appear to be more specific than the inhibitors/degraders.

4. While the model of R-loop associated replication stress rests on considerable literature, the idea that 'deregulated transcription' leads to these events in BRD4-depleted cells is totally unclear. In the discussion the idea is raised that transcriptional pausing in the absence of BRD4 is increasing the opportunity of a nascent RNA to re-anneal with the template strand to create an R-loop. However, the authors do not provide much data to support this view. As above, additional experiments targeting a specific locus that accumulates R-loops in BRD4 depleted cells for co-accumulation of paused RNA polymerase could be important.

5. In general a clearer statement of the proposed mechanism (i.e. what does transcriptional deregulation mean as a predisposing factor for R-loop induced DNA damage) and supporting data

would significantly increase my enthusiasm for this study. There are many possible experiments that would be helpful and I don't mean to suggest that all avenues need to be explored, but testing some specific models is required.
For example, if RNA-seq data on transcriptional dysregulation after BRD protein depletion was available, then correlating these data with published R-loop maps might be instructive (i.e. if you predict that promoter-proximal pausing is driving R-loops then perhaps BRD4 targets would be R-loop prone). Alternatively, many groups have now had success using proximity ligation to show R-loop association in cells, or an increase in replication-transcription conflicts in situ.

Minor points:
The authors state that "Despite potent suppression of MYC, treatment with triptolide alone did not result in increased DNA damage signaling, DSBs, or apoptosis in cells…". Surely triptolide would eventually kill cells. I think the time frame of the experiment is what allows the observations with triptolide to exert the effects seen and that this should be acknowledged.

- Why is the time frame of ARV-825 treatment in Figure 3 (6 hours), different than in Figures 1 and 2 (3 hours)? It would seem that consistency between experiments or an explanation would be preferred.

- The authors state in the discussion that "… it cannot be excluded that the RNA:DNA hybrid within the transcription bubble can remain annealed and be extended as RNAP moves forward." How likely do the authors think this is? RNAPII extrudes nascent RNA through an exit tunnel that is physically displaced from the DNA template. This just seemed unlikely to be correct based on what we know about RNAPII.

Reviewers' comments:

We are grateful to all of the reviewers for their helpful comments and suggestions on how to improve the manuscript.

Reviewer #1 (Remarks to the Author):

This manuscript provides new insights into how DSBs are induced by inhibition of BET family proteins including BRD4. Previously, these authors demonstrated that a variant of BRD4 protected chromatin from DNA damage response by insulating chromatin from DNA damage signaling. The source of this damage upon inhibition of BET family was not clear. Here, this manuscript tries to address this question by showing that BRD4 inhibition induces transcription-replication conflicts as a result of R-loop formation. This suggests that the BET family proteins are important for the correct loading of transcription co-activator complexes such as pTEFb to regulate transcription and avoid R-loop formation. The observation that R-loop formation by BRD4 inhibition is novel and builds upon this groups previous work showing that inhibition of BRD4 induced DNA damage responses. Overall this work is interesting but there are still several issues that need to be addressed before publication.

Major comment:

1. Inhibition of BRD4 induced transcription-replication conflicts in S phase. In this state, BRD4 knock down cells are likely to be arrested in S and G2 phase. However, in these groups previous studies, BRD4 knockdown cells do not display any cell cycle defects (Scott R. Floyd et al., Nature, 2013). In addition, JQ1 treatment also did not induce phospho-H2AX (Figure 4e of this paper). One possibility is that this phenomenon occurs in a sub-set of cancer cells and its mechanisms are not universal. These data seem at odds and need to be explained.

This is a good point. We appreciate the reviewer's careful read of our prior work and astute observations. We would point out that in our previous work, we found BRD4 as a modulator of the DNA damage response to ***ionizing radiation,*** and that for those prior experiments, the source of DNA damage was ionizing radiation. The original screen, and nearly all followup experiments were performed on ***irradiated*** cells, and in cell lines that were viable after BRD4 loss-of-function. Additionally, for drug experiments (JQ1), the ***doses were modest (250 nM)*** and we exposed cells for ***minimal times (4-16 hours)*** prior to irradiation. Importantly, we and others have found variable responses to BRD4 LOF in the absence of radiation in different cell types, and these findings agree with published reports of variable responses of cell lines and tissues to BET bromodomain inhibitors (BDi). In our prior study, the principle cell line used for most experiments was U2OS, where we observed very minimal, if any induction of DNA damage with prototypical BET-bromodomain inhibitors (BDi) such as JQ1 in unirradiated cells. This result has now been replicated in a recently-published study using the U2OS cell line (Bowry *et al.,* Cell Reports 2018).

In fact, it was the stark contrast between the effects of JQ1 on (unirradiated) U2OS cells and MYC-driven lymphoma cells (Emu-Myc model of Burkitt's lymphoma) that was the original impetus for the current study (please see accompanying Reviewer Fig. 1 comparing effects in U2OS and Emu-Myc cells).

As a specific example, we also compared the effects of JQ1 on (unirradiated) untransformed mouse fibroblasts, and fibroblasts expressing mutant K-ras via cre-lox induction of a Lox-STOP-Lox K-RasG12D allele. Normal cells show no appreciable γH2AX or phospho-RPA S33 response to JQ1, while the K-ras -transformed cells show a robust reponse to JQ1 (Reviewer Fig. 2). These data indicate that oncogene transformation can induce susceptibility to BRD4 inhibition, a result that is in line with other published reports on transcription-replication conflicts in oncogene-transformed cells (Kotsantis et al*., Nat Comm*, 2016).

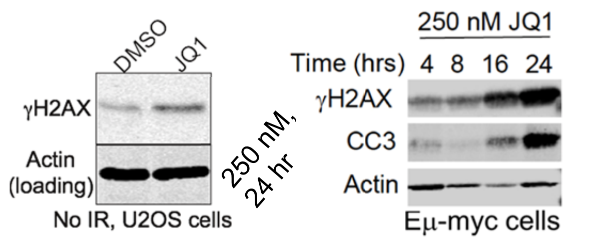

Review Fig. 1: effects of JQ1 on U2OS and Emu-Myc cells. U2OS were exposed to JQ1 for 12 hours, causing modest increase un γH2AX, while exposure of Emu-myc lymphoma cells caused robust γH2AX and apoptotic cell death.

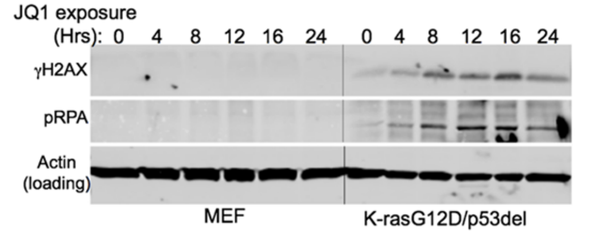

Review Fig. 2: JQ1 effects in normal and Ras-transformed fibroblasts. Mouse embryonic fibroblasts, or mutant Ras expressing fibroblasts were exposed to JQ1 for indicated times and γH2AX and phospho-S33 RPA measured.

We now explicitly address this in the revised Discussion section (pages 24), where we state, citing the appropriate references:

"Importantly, the ability of bromodomain inhibition to cause DNA damage and replication stress varies across cancer cell lines. Our previous work characterizing the effects of bromodomain inhibition on ionizing radiation-induced DNA damage in U2OS cells showed that exposure of U2OS cells to JQ1 alone did not elicit a DNA damage response[1], in contrast to what we observed here in HeLa, HCT 116, and previously in U87MG and GL261 cells[2], and what others have observed in human myeloid leukemic cell lines[3]. We further characterized this absence of DNA damage signaling (Fig. S10A) and double-strand break formation (Fig. S10B) in U2OS cells following prolonged treatment with 1 μM JQ1, suggesting that these cells are inherently insensitive to bromodomain inhibitor-induced DNA damage. This finding was recapitulated in a study by Zhang et al in which they failed to detect DNA damage or cell cycle perturbation in U2OS cells treated with another bromodomain inhibitor, AZD5153, although they did observe elevated γH2AX levels in OVCAR3 and OVCAR4 cells treated with the same drug for 24 hrs[4]. Furthermore, while bromodomain inhibition alone in U2OS had little effect, it synergized with replication stress-inducing agents including HU-treatment and with ATR inhibition to cause increased γH2AX signaling. Taken together, their findings and ours suggest that BRD4 plays an important general role in regulating replication stress responses, and that the effect of BRD4 inhibitors alone on inducing DNA damage in cancer cells may depend on the basal level of oncogene-induced replication stress. Additional experiments will be required to confirm or refute this hypothesis."

If the reviewer would like, we can also include the Reviewer Figures 1 and 2 as Supplemental Figures, as long as the journal policy limiting the number of supplemental figures is congruent with this.

2. Given that the authors claim that these effects occur in S-phase, cell cycle analyses for all key experiments need to be performed. This includes Triptolide and RNase H1 o/e analyses. In addition, growth curves -/+ JQ1 with and without RNase H1 should be performed to see how these treatments effect cell proliferation and not just the phenotypic readouts of DNA damage.

As requested, we have now performed cell cycle analyses in HeLa (Fig. 9A), HCT116 (Fig. 9B), and RNase H-inducible HeLa cells in the presence and absence of induced RNase H expression (Figure 9C), following treatment with 500 nM JQ1 for 48 hrs, as well as growth curves for cells treated with JQ1 or DMSO in the presence or absence of RNase H expression (Figure 9D). Induction of RNase H expression in HeLa cells restored the cell cycle distribution to pretreatment levels [Fig. 9C (+RNase H)] and also partially rescued the JQ1-induced block in cell proliferation (Fig. 9D), suggesting that removal of transcriptional roadblocks cause by the accumulation of R-loops following BET bromodomain inhibition leads to resolution of transcription-replication conflicts and restoration of normal cell cycle kinetics.

These results are now described on pages 18 of the revised manuscript:

"Our findings that BET bromodomain protein loss or inhibition causes R-loop-mediated replication stress, fork stalling, and DNA damage led us to investigate whether these effects resulted in subsequent changes in cell cycle progression/distribution and checkpoint signaling. Cell cycle analysis using flow cytometry following bromodomain inhibition with 48 hrs of JQ1 demonstrated a slight increase in the percentage of both G_1 and G_2/M cells and a corresponding decrease in the percentage of S-phase cells (Figs. 9A and 9B, HeLa and HCT116 cells respectively). Our results showing G_1 accumulation with fewer S phase cells agrees with studies from Ozato's lab colleagues that identified multiple roles for BRD4 in modulating entry into S-phase[5], including promoting transcription of genes important for G_1/S progression[6] and with our prior observation that bromodomain inhibition or degradation decreases the percentage of EdU + cells (Figs. 8C and S7C). The increased population of cells in G_2/M, however, was unexpected, suggesting possible aberrant signaling of the intra-S or G_2/M checkpoint in the presence of S phase DNA damage. Since induction of RNase H1 expression abrogated the DNA damage signaling and apoptosis in cells treated with either ARV-825 or JQ1 (Figs. 3B and S3B, respectively), we examined whether the induction of RNase H1 in JQ1-treated HeLa cells would also restore the cell cycle distribution of HeLa cells to its pre-treatment level. Indeed, as shown in Figure 9C, the modest increase in G_1 and G_2/M populations, and reduction in the S-phase population caused by JQ1 treatment was abrogated by RNase H1 expression, which also partially rescued the JQ1-induced block in cell proliferation (Fig. 9D). These results suggest that removal of transcriptional roadblocks caused by the accumulation of R-loops following BET bromodomain protein loss leads to resolution of TRCs and restoration

of normal cell cycle kinetics." The phenotypic and mechanistic basis for these cell cycle changes are then further explored in the next section of the revised manuscript.

3. The data in figure 5B, in the quantification graph show that BRD4 knock down cells only increased phospho-H2AX level but in WB image, BRD3 knockdown cells also increased phospho-H2AX even if the actin level was less than control and BRD4 knockdown cells. The graph and WB image seem inconsistent. Given the poor quality of these western blots, new data should be provided and for these experiments unmodified H2AX should be used and total PARP staining should be analyzed.

As requested, we have repeated these experiments several more times. As now shown in Figure 4B, by western blot, only knockdown of BRD4 leads to increases in γH2AX and PARP cleavage products (probed using a total PARP antibody) without significant changes in total H2AX levels, consistent with the IF images (Fig. 4C). These results are highlighted on page 9 of the revised manuscript.

4. There are three isoforms of BRD4 in the cells (Scott R. Floyd et al., Nature, 2013). Which BRD4 isoform affects R-loop formation?

To address this, we performed isoform-specific BRD4 knockdown and rescue experiments. Using siRNA against isoform A, B, and C (new Fig. 5A), we now show that knockdown of isoform A but not isoforms B or C leads to increased nuclear S9.6 and γH2AX immunostaining on immunofluorescence (IF), indicating that it is isoform A that affects R-loop accumulation and DNA damaging signaling. We next performed rescue experiments using transient expression of GFP-tagged isoform A or C (Isoforms A and C were examined because of their well-established functions in effecting transcription, where little is known for the role of isoform B in transcriptional control). Immunostaining for γH2AX revealed that cells expressing GFP-Iso A had significantly reduced γH2AX DNA damage foci and nuclear γH2AX intensity following treatment with JQ1 (new Fig. 5C, upper panel, solid arrows) compared to non-expressing cells (Fig. 5C, upper panel, open arrows). In contrast, cells that transiently expressed GFP-Iso C showed persistently increased numbers of γH2AX foci and nuclear γH2AX intensity following treatment with JQ1 (Fig. 5C lower panel, solid arrows) that was essentially identical to that observed in cells that did not express GFP-Iso C (Fig. 5C lower panel, open arrows). These results indicate that the normal function of the long isoform of BRD4 in transcriptional elongation prevents transcription-associated DNA damage in cells. These results are now discussed in detail in a new section of the manuscript entitled "The long isoform of BRD4 suppresses R-loop mediated DNA damage" which is on pages 11 to 12 of the revised manuscript.

5. In this paper, BRD4 inhibition induces DNA damage and it would be interesting to know if this relates with deregulated transcription. Is it dependent on dysfunction of pTEFb on transcription? One might think that BRD4 inhibition should reduce transcription. Similarly, Triptolide also inhibits transcription but has opposite effects. This should be explained.

The reviewer raises an excellent point: we find that the DNA damage effects of BRD4 inhibition are closely tied to its roles in transcriptional elongation, which likely includes its role as a coactivator of pTEFb. As mentioned above, knockdown of Isoform A (which contains the C-terminal pTEFb interacting domain), but not isoform B or C, leads to increased DNA damage signaling (γH2AX foci) and R-loops (nuclear S9.6 staining) suggesting that interaction with pTEFb contributes to BRD4's role in regulating the DNA damage response (Fig. 5B). Similarly, as mentioned above, rescue experiments using transient overexpression of GFP-Iso A abrogated the DNA damage effects of JQ1 but not GFP-Iso C (Fig. 5C), again suggesting that interaction between BRD4, pTEFb and RNA Pol II is required for preventing deregulated transcription. However, BRD4 also plays a number of additional roles in regulating transcriptional elongation besides helping to recruit pTEFb to facilitate the switch from transcriptional initiation to elongation. Keiko Ozato's group has shown that BRD4 is required for processive elongation of RNA Pol II all along the gene body of BRD4-dependent genes independently of its pTEFb recruitment function[7], while a paper from Keh-Chuang Chin's lab examining BRD4 function in CD4+ T-cells showed that only 20%of BRD4 binding sites mapped by ChIP to proximal promoters, with the vast majority of BRD4 binding sites localizing within gene bodies and intragenic regions. Furthermore, JQ1 treatment resulted in reduced RNA Pol II Ser2 phosphorylation at both the promoters and throughout the gene bodies in that study[8]. Our demonstration of persistent R-loops, increased γH2AX, and decreased Ser2 phosphorylated RNA Pol II throughout the bodies of affected genes, shown in the new data presented in the revised manuscript (Figs 7, S5 and S6) is in excellent agreement with this work. Finally, we now also show that interference with BRD4 function also impairs the transcription of several key proteins involved in R-loop suppression (SETX, DHX9, SRSF1 in Fig. 7). These data indicate that there are multiple mechanisms by which BRD4 loss contributes to deregulated transcriptional control. This is summarized in the highlighted

section of the revised Discussion on pages 21-24.

Regarding BRD4 inhibition and total transcriptional activity - interestingly, it turns out that BRD4 inhibition alone does not significantly reduce global gene transcription. A recent publication that was published after our initial submission shows that bromodomain inhibition actually increases transcription of histone and non-polydadenylated non-coding RNA genes, causing transcription-replication conflicts and replication fork slowing (Bowry et al, *Cell Reports*, 2018)[9]. When we directly measured EU incorporation in our triptolide and ARV-825 experiments, we did observe an apparent ~35% decrease in EU incorporation (signifying de novo RNA synthesis) following treatment with ARV-825 alone (Figs 2A and S2A), but this failed to reach statistical significance, suggesting that bromodomain protein loss does not globally inhibit transcription. In contrast, triptolide significantly did reduce EU incorporation and also abrogated DNA damage signaling (nuclear γ2AX intensity) (Figs. 2A and S2A), and double-strand break formation (Figs. 2C and S2B) following bromodomain protein degradation by ARV-825 co-treatment, supporting our model that removal of transcriptional roadblocks that are caused by BRD4 loss, by either collapse of transcription bubbles following triptolide treatment, or removal of R-loops by RNase H, prevents transcription-replication conflicts and DNA damage induced by BRD4 inhibition. This is described in detail on pages 6-8 of the revised manuscript.

6. Based on these data, it is curious that BRD4 isoform A knock down in cells does not increase phospho-H2AX levels in undamaged cells ((Scott R. Floyd et al., Nature, 2013)? BRD4 isoform A contain the CTM domain, which is critical for interaction with pTEFb. Does BRD4 CTM mutant show the same phenotype as BRD4 knock down?

We very much appreciate of the reviewer's careful reading of our prior work. As explained in our response to points 1 and 4 above, the prior study was conducted in U2OS cells, which do not exhibit DNA damage upon BRD4 inhibition in the absence of exogenous sources of DNA damage. These findings in U2OS cells are now corroborated by a recent study (Bowry et al., *Cell Reports*, 2018)[9] indicating that BRD4 inhibition does not lead to DNA damage in this cell line. Please refer to our response to question 4 above outlining that our results in Fig. 5B show increased accumulation of R-loops and DNA damage following knockdown of BRD4 isoform A (which contains the P-TEFb interaction domain in its C-terminal) while knockdown of BRD4 isoforms B or C do not induce DNA damage. While we have not directly tested the necessity of the C-terminal interaction domain in causing this damage phenotype via mutation experiments, our isoform A rescue experiment (Fig. 5C) indicates that the P-TEFb-interacting C-terminal domain is essential in abrogating the DNA damage phenotype. These results are discussed in detail in the new highlighted text on pages 11 to 12 of the revised manuscript.

7. If R-loop formation induces DNA damage, why do the foci of S9.6 not co-localize with DNA damage markers. This is a major issue that must be fully addressed. One possibility is that the phospho-H2AX seen is a result from apoptosis and not R-loops. All the data shown shows that phospho-H2AX and apoptosis are occurring. Perhaps altering treatment conditions could provide a scenario where DNA damage is induced but not enough to provoke apoptosis. One could also block apoptosis and show that DNA damage still occurs.

We are very grateful to the reviewer for this observation and have now performed the excellent experiment she/he suggested in which we co-treated cells with the pan-caspase inhibitor ZVAD-FMK in the presence of JQ1 to decrease the diffuse nuclear distribution of γH2AX immunostaining due to apoptosis (Fig. 6A) and have been able to show that a subset of γH2AX foci co-localizes with S9.6 R-loop immunostaining (Fig. 6B, i-iv). These results are now described on page 13 of the revised manuscript, where we state:

"It has recently been shown that while cells have the propensity to accumulate R-loops at multiple loci when natural processes of R-loop removal are affected (i.e. loss of RNase-H function)[10], DNA damage appears to occur at only a fraction of these sites[11]. This, coupled with other observations that the persistence of a subset of R-loops can impair the expression of specific genes [12-14] led us to examine whether a subset of R-loops is responsible for the DNA damage foci observed upon BRD4 inhibition in cells. To address this, cells were co-stained for γH2AX and DNA-RNA hybrids using the S9.6 monoclonal antibody. Importantly, because prolonged bromodomain inhibition results in pan-nuclear γH2AX foci on a faint diffuse γH2AX staining background, likely as a consequence of apotosis (Figs. 1A and 1F), we reduced the treatment time and used a slightly higher amount of JQ1 (1 µM) in combination with the pan-caspase inhibitor ZVAD-FMK (Fig 6A). Treatment of cells with 1 µM JQ1 alone for 8 hrs still resulted in pan-nuclear γH2AX foci (Fig. 6A, JQ1), however, co-treatment of cells with JQ1 and ZVAD-FMK eliminated the faint diffuse γH2AX background, and resulted in fewer but brighter, more well-defined γH2AX foci, without significantly affecting the S9.6 staining

(Fig. 6A, JQ1+ZVAD). This confirms that it is a specific subset of R-loops are associated with DNA damage foci (Fig. 6B, i-iv), suggesting that not every R-loop causes DNA damage following BRD4 inhibition."

Importantly, we then performed genome-wide BRD4 and γH2AX ChIP-Seq experiments to identify the subset of BRD4, JMJD6, and CHD4 co-regulated genes that demonstrated increased DNA damage following bromodomain inhibition (Figs. 7A, B and S5). S9.6 DNA:RNA hybrid IP (DRIP) followed by qPCR (DRIP-qPCR) showed increased accumulation of R-loops at these select gene loci following JQ1 treatment (Figs. 7C and S6), further corroborating our immunofluorescence experiments in Fig. 6 that a subset of DNA damage foci colocalizes with increased R-loops following bromodomain inhibition. These results are now discussed in the new highlighted text on pages 13 to 15 of the revised manuscript.

Minor comment:

1. In the figure1, panel C, phospho-H2AX should be white bar not black.

We thank the reviewer for noticing this error and we have correctly labeled the bar white.

2. In page 6, 2nd low, DBS formation should be DSB formation.

We have corrected this typo.

Reviewer #2 (Remarks to the Author):

In this manuscript, the authors uncover a previously unappreciated function of BET bromodomain proteins, particularly BRD4, in the regulation transcription-replication conflicts by preventing R-loop formation. They show that inhibition of BET bromodomain proteins leads to increased DSB formation and apoptosis in different cancer cell lines. The authors use a specific transcription initiation inhibitor (triptolide) to show that the DNA damage and apoptotic phenotype observed with BET bromodomain inhibitors requires active transcription. Next, they propose that this damage originates primarily as a consequence of replication-transcription conflicts because it occurs primarily within the S-phase. Finally, they conclude that DNA damage is due to the accumulation of R-loops by combining experiments with the S9.6 antibody that specifically detects R-loops and RNase H1 that suppresses R-loop formation. Lastly, they selectively knockdown distinct BET bromodomain proteins by siRNA and suggest that BRD4 is primarily responsible for the observed phenotype. Collectively, this is an interesting study that points to a novel function of BET bromodomain proteins in regulating R-loop formation and replication-transcription conflicts. The studies are however too preliminary and have not been developed to the point that it should be considered for publication in a high-profile journal.

Major concerns:

1. The authors' statement that BET bromodomain proteins are important to prevent R-loop formation is not supported by the data. The authors cannot distinguish whether loss/inhibition of BET bromodomain proteins leads to increased R-loop accumulation because these proteins are needed to prevent R-loop formation, as the authors suggest, or to favor R-loop resolution. The authors should include further experiments to distinguish between these two alternative possibilities.

We thank the reviewer for this observation and suggestion, and have performed additional experiments to distinguish between the alternative possibilities, as shown in the new Figures 6, 7, S5, and S6. In fact, the reviewer was absolutely correct; the main effect of BRD4 loss of function is failure of R-loop resolution. We have changed the text throughout to now indicate that BET bromodomain proteins are required to prevent R-loop accumulation, rather than to prevent R-loop formation, since the accumulation of R-loops upon BRD4 loss or inhibition is what the experimental data actually show. Moreover, we also followed the reviewer's advice and performed genome-wide BRD4, γH2AX, and RNA pol II ser2 ChIP-Seq experiments in the presence of JQ1 and have also observed transcriptional downregulation of genes involved that have been shown to be involved in R-loop suppression and transcriptional termination, namely senataxin (Cohen et al, *Nat Commun*, 2018)[15], DHX9 (Cristini et al, *Cell Reports*, 2018)[16], SRSF1 (Cristini et al, *Cell Reports*, 2018)[16], and SRSF2 (Chen et al, *Mol Cell*, 2018)[17], resulting in potent downregulation of senataxin protein levels by 24 hrs of JQ1 treatment, followed by DHX9, and SRSF1 by 28 hrs (Fig. 7F). Our DRIP-qPCR results also show increased accumulation of R-loops at BRD4, JMJD6, and CHD4 co-regulated genes following bromodomain inhibition (Figs. 7C and S6). Taken together, these new results best support a model where BRD4 inhibition results in R-loop accumulation by interfering with efficient transcriptional elongation, and is further contributed to by the subsequent loss of R-loop processing factors, rather than by preventing R-loop formation in the first place. These results are now reported in detail in the new text on pages 13 to 15 of the revised manuscript. Thank you again for noticing this poor choice of initial wording and for suggesting experiments to further clarify the

mechanism.

2. The authors should include more experiments to provide mechanistic insights into the actual role of BRD4 in R-loop regulation.

We thank the reviewer for this suggestion. We believe that BRD4 plays multiple roles in R-loop regulation. First, our RNAPII ser2 ChIP-Seq experiments show corresponding JQ1-dependent decreases in RNAPII ser2 at the promoters and throughout the entire gene bodies of BRD4 regulated genes, indicating a reduction in productive RNAPII elongation along these genes (Figs. 7B and S5). This data is in excellent agreement with published data from Ozato and colleagues[7], and Chin and colleagues[8] that BRD4 plays a critical role in transcriptional elongation throughout the gene body by interacting with acetylated histones and also correlates with a study by Rosenfeld and colleagues that BRD4 loss of function leads to RNAPII stalling as evidenced by decreased RNAPII travelling ratios at RNAP II transcribed genes[18]. Fu and colleagues have shown that RNAPII stalling leads to the accumulation of R-loops in cells[19]. Our DRIP-qPCR experiments show accumulation of R-loops along gene bodies of a subset of BRD4, JMJD6, and CHD4 co-regulated genes following bromodomain inhibition (Figs. 7C and S6), suggesting that RNAP II stalling following BRD4 inhibition contributes to the accumulation of R-loops. Second, loss of function of the DNA helicase senataxin (SETX) prevents the resolution of R-loops at the 3' end of genes, causing further RNAPII stalling distal to the polyadenylation sequence and defective transcription termination[15]. This is also seen in cells following loss of other R-loop processing factors including DHX9 and SRSF1 [16]. Our BRD4 ChIP-Seq experiments demonstrate transcription downregulation of *SETX, DHX9, and SRSF1* following bromodomain inhibition (Figs. 7B, D-F), suggesting that BRD4 is involved in transcriptional maintenance of key R-loop processing factors. (See also our response to point 5 of Reviewer 1). These results are now presented on pages 13 to 15 of the revised manuscript, the text of which is reproduced in the response to point 3 immediately below. In addition, these multiple mechanisms by which BRD4 contributes to R-loop regulation are summarized on pages 21-24 of the revised Discussion.

3. The authors should explore the role of the previously reported interactions of BRD4 with the positive elongation factor (P-TEFb) and the transcriptional co-activators JMJD6 and CHD4 in regulating transcription-replication conflicts and R-loop formation.

Another excellent suggestion. The role of BRD4 interaction with P-TEFb was explored using isoform-specific knockdowns and rescue experiments, which are summarized in our response to Reviewer #1, point 4. In brief, only knock-down of BRD4 isoform A, with its P-TEFb-interacting C-terminal domain, resulted in R-loop formation and DNA damage signaling, while exogenous expression of isoform A, but not isoform C, blunted the effects of BET bromodomain inhibition by JQ1 (Figures 5B and 5C, respectively). The roles of the transcriptional co-activators JMJD6 and CHD4 was explored by performing BRD4, γH2AX, and RNA pol II ser2 ChIP-Seq and then mapping the increased DNA damage and R-loop accumulation to 297 known BRD4 and JMJD6 co-regulated genes and to 205 known BRD4 and CHD4 co-regulated genes (Figs. 7, S5, and S6). These results suggest BRD4 inhibition leads to deregulated transcription and transcription-associated DNA damage largely at loci that require a functional interaction between BRD4 and JMJD6 or BRD4 and CHD4. These results are now reported on pages 13 to 15 of the revised text where we state:

"To further identify the regions of the genome that were susceptible to R-loop-associated DNA damage following bromodomain inhibition, we performed BRD4, γH2AX, and RNAPII ser2 chromatin immunoprecipitation followed by massive parallel sequencing (ChIP-Seq). Alignment of BRD4, γH2AX, and RNAPII ser2 ChIP-Seq peaks identified greater than 2-fold enrichment of γH2AX at 39 genes that are known to be regulated by BRD4 in humans following treatment of cells with JQ1 (Fig. 7A and Table 1)[18]. As transcriptional regulation of genes by BRD4 often require interactions with coregulatory proteins including JMJD6 and CHD4[20], we also looked for enrichment of γH2AX at genes that are known to be co-regulated by BRD4 and JMJD6 [18] or by BRD4 and CHD4[20] and found enrichment at 297 genes that are known to be co-regulated by the former, and 205 genes that are known to be co-regulated by the latter following JQ1 treatment (Fig. 7A and Table 1). Representative ChIP-Seq profiles for 11 of these genes, belonging to all three classes, are shown in Fig. S5. Alignment of BRD4, γH2AX, and RNAPII ser2 ChIP-Seq peaks with known H3K4Ac histone ChIP-Seq peaks from existing ENCODE datasets demonstrated that DNA damage at these BRD4 co-regulated genes correlates with mapped regions of active transcription, (c.f. Fig. S5) further supporting our hypothesis that deregulated transcription following bromodomain inhibition leads to DNA damage. We observed a corresponding JQ1-dependent decrease in BRD4 and RNAPII ser 2 at the promoters and throughout the gene bodies indicating a reduction in productive RNAPII elongation along the gene.

Interestingly, γH2AX enrichment following JQ1 treatment also not only occurred at the promoter regions where BRD4 is known to be enriched, but was similarly propagated throughout the entire gene body (Fig. S5), consistent with DNA damage occurring throughout the gene.

We next performed DNA-RNA hybrid immunoprecipitation (DRIP) using the S9.6 antibody followed by qPCR (DRIP-qPCR) on a subset of the genes that were identified to have increased DNA damage following treatment with JQ1 in order to characterize regions within these genes that were susceptible to the accumulation of R-loops. DRIP-qPCR showed increased relative abundance of R-loops throughout the transcription start site, exonic, intronic, and termination regions of BRD4 co-regulated genes when the cells were treated with JQ1 (Fig. S6, red bars). This finding is consistent with our prior S9.6 immunofluorescence results (Fig. S3A and 6A, JQ1 alone). To confirm the specificity of DNA:RNA hybrid enrichment, we pretreated whole genomic extracts with RNase H1 to specifically degrade R-loops prior to performing DRIP. This resulted in loss of the qPCR signal, indicating that the signal enrichment was due to the accumulation of R-loops throughout the gene bodies at these loci following bromodomain inhibition (Fig. S6, blue bars), again consistent with the loss of S9.6 immunofluoresence signal in cells following induced expression of RNAseH1 (Fig. S3B). Taken together, these ChIP-Seq and DRIP-qPCR data demonstrate increased DNA-RNA hybrids in a subset of BRD4-regulated genes that show lower levels of actively elongating RNA Pol II, consistent with increased RNA Pol II stalling, pausing, and/or decreased R-loop resolution.

Cristini et al.[16] performed mass spectrometry on S9.6 immunopreciptates in HeLa cells and identified key proteins in the R-loop interactome that are involved in R-loop suppression and transcription termination. That study identified previously known proteins involved in R-loop biology such senataxin (SETX) and the serine/arginine-rich splicing factor 1 (SRSF1), and also identified novel functions for the helicase DHX9 in R-loop suppression and transcription termination. Intriguingly, transcription of *DHX9* is known to be regulated by CHD4[21]. Our ChIP-Seq analysis identified loss of BRD4 and RNAPII ser2 with increased γH2AX at *DHX9* (Fig. 7B) with increased R-loop accumulation throughout the gene body following treatment with JQ1 (Fig. 7C). This corresponded to gradual suppression of DHX9 protein levels over a 48 hr time course of JQ1 treatment (Fig. 7F). Transcription of *SETX* and *SRSF1* has not been previously reported to be regulated by BRD4, however, analysis of the *SETX* and *SRSF1* loci, showed BRD4 and RNAPII ser2 peaks at their promoters under basal DMSO conditions which were reduced following treatment with JQ1 (Figs. 7D and E, respectively), along with correspondingly decreased SETX and SRSF1 protein levels (Fig. 7F). Taken together, these data are in good agreement with previously established roles for BRD4 in transcriptional elongation[7, 8, 20, 22-26], and suggest novel roles for BRD4 in regulating R-loop suppression and R-loop-associated DNA damage at a subset of BRD4-regulated or co-regulated genes, including several genes which are themselves involved in mechanisms of R-loop suppression (Fig. 7G)."

4. The authors should include additional experiments to strengthen their conclusion that the DNA damage observed with BET bromodomain inhibitors originates from transcription-replication conflicts. For example, they could use single-molecule DNA fiber assays to directly monitor replication perturbations and demonstrate that replication is impaired when cells are treated with the with BET bromodomain inhibitors.

We thank the reviewer for this suggestion and have performed the suggested DNA fiber combing assays in the presence of a BET bromodomain degrader ARV-825 and the BET bromodomain inhibitor JQ1. Our results show reduced incorporation of labelled nucleotide analogues (Figs. 8D and S7D) and marked slowing of the replication fork in HeLa and HCT116 cells (Figs. 8E and S7E, respectively) following treatment with ARV-825 and JQ1. Replication fork stalling following JQ1 treatment has also been previously demonstrated by Da Costa et al, in which they also show decreased nucleotide analogue incorporation and reduced fork speeds (Fig. 6, Da Costa et al, *Blood Cancer J*, 2013)[27]. These results are described in the new text, highlighted on page 17 of the revised manuscript.

5. The authors should include a quantification for the EU incorporation experiments shown in Figure 2A.

We thank the reviewer for this suggestion. We have now quantified the EU incorporation and γH2AX intensity as requested in Figure 2A. These results are shown in the left panels of the new Figure 2A, and described on page 8 of the manuscript where we state:

"HeLa cells treated with triptolide showed significantly decreased levels of EU incorporation (Fig. 2A) and suppression of MYC protein levels (Fig. 2B), demonstrating inhibition of transcription. …..

Despite potent suppression of MYC, treatment with triptolide alone did not result in increased DNA damage signaling, DSBs, or apoptosis in cells, while treatment with ARV-825 alone was again associated with increased DNA damage, PARP-mediated apoptosis and DSB formation over the same time course (Figs. 2A, C and D)….. Cells pretreated with triptolide followed by co-treatment with ARV-825 showed abrogation of DNA damage signaling, DSB formation, and apoptosis (Figs. 2A, C and D, respectively), suggesting that DNA damage caused by BRD4 loss requires the presence of active transcription bubbles."

6. Figure 3. The authors should measure the percentage of S-phase cells. On the basis of the authors' model that R-loop accumulation perturbs replication progression, they might find an increased percentage of S-phase cells following treatment with BET bromodomain inhibitors, due to S-phase arrest. Along the same line, they might find a decreased EdU incorporation because of the increased fork stalling associated with R-loop accumulation.

Another excellent point. As requested we performed the proposed cell cycle experiments, as described/summarized in our response to Reviewer 1 point 2, which is reproduced below:

"Cell cycle analysis using flow cytometry following bromodomain inhibition with 48 hrs of JQ1 demonstrated a slight increase in the percentage of both G_1 and G_2/M cells and a corresponding decrease in the percentage of S-phase cells (Figs. 9A and 9B, HeLa and HCT116 cells respectively). Our results showing G_1 accumulation with fewer S phase cells agrees with studies from Ozato's lab colleagues that identified multiple roles for BRD4 in modulating entry into S-phase[5], including promoting transcription of genes important for G_1/S progression[6] and with our prior observation that bromodomain inhibition or degradation decreases the percentage of EdU + cells (Figs. 8C and S7C). The increased population of cells in G_2/M, however, was unexpected, suggesting possible aberrant signaling of the intra-S or G_2/M checkpoint in the presence of S phase DNA damage. Since induction of RNase H1 expression abrogated the DNA damage signaling and apoptosis in cells treated with either ARV-825 or JQ1 (Figs. 3B and S3B, respectively), we examined whether the induction of RNase H1 in JQ1-treated HeLa cells would also restore the cell cycle distribution of HeLa cells to its pre-treatment level. Indeed, as shown in Figure 9C, the modest increase in G_1 and G_2/M populations, and reduction in the S-phase population caused by JQ1 treatment was abrogated by RNase H1 expression, which also partially rescued the JQ1-induced block in cell proliferation (Fig. 9D). These results suggest that removal of transcriptional roadblocks caused by the accumulation of R-loops following BET bromodomain protein loss leads to resolution of TRCs and restoration of normal cell cycle kinetics." The phenotypic and mechanistic basis for these cell cycle changes are then further explored in the next section of the revised manuscript.

Interestingly, our results showed a decrease in percentage of S-phase cells following treatment with JQ1 in HeLa, HCT116, and RNase H-inducible HeLa cells in the absence of RNase H [Figs. 9A, B, and C (left panel, - RNase H), respectively]. We go on to show that this paradoxic decrease in the S-phase population results from an increase in cell death of the DNA-damaged S-phase cells following bromodomain inhibition (Figs. 8A and 10F) accompanied by downregulation of the ATR-TopBP1-Chk1 replication stress DNA damage checkpoint due to transcription downregulation of TopBP1 following BRD4 inhibition (Figs. 10B-E and S9A-D), leading to increased slippage of cells into mitosis and death by mitotic catastrophe (Figs. 10F and S9E). Indeed, induced expression of RNase H in HeLa cells partially rescues these effects of JQ1 on cell cycle perturbations (Fig. 9C, right panel, + RNase H) and partially restores normal cellular proliferation (Fig. 9D). These results are described in the new text, highlighted on pages 18 to 20 of the revised manuscript.

Furthermore, exactly as hypothesized by the reviewer, we did observe decreased EdU incorporation following bromodomain inhibition in HeLa and HCT116 cells (Figs. 8C and S7C) consistent with the observed increased replication fork stalling (Figs. 8D-E and S7D-E).

Reviewer #3 (Remarks to the Author):

This article describes the discovery that BRD4 inhibition leads to transcription-coupled DNA damage. This damage is at least partly driven by the accumulation of R-loops which the authors speculate is due to transcriptional dysregulation. Overall the story presented is very straightforward and the authors have been rigorous in applying multiple reagents to inhibit or deplete BRD proteins and show their results are consistent in different cell lines. The flip side of this is that the story can be viewed as quite superficial from a mechanistic perspective, and the authors themselves point out that the mechanism of R-loop induced DNA damage in BRD4-depleted cells remains unknown, even concluding the discussion with a suggested experiment: DRIP-sequencing of BRD4-depleted cells.

The work is solid and supports the conclusions, but is at an observational stage, rather than a mechanistic stage. The observations are interesting and below I make suggestions for improvements.

1. The S9.6 antibody has now been used repeatedly for precipitation of R-loops. I suggest that DRIP experiments, followed by qPCR at known BRD4-target loci, (or by deep sequencing if feasible), would enhance the work. It would also allow some direct tests of mechanisms. For example, to show that BRD4 ChIPs to the same locus, then upon depletion, R-loops accumulate at that locus. This would at least implicate a direct model of R-loop induction rather than other indirect effects on gene expression networks.

We thank the reviewer for this excellent suggestion and have performed the suggested DRIP-qPCR using the S9.6 antibody at BRD4, JMJD6, and CHD4 co-regulated loci demonstrating increased relative abundance of R-loops throughout these loci following treatment with JQ1 (Figs. 7C and S6, red bars). This increased abundance of R-loops corresponds to depletion of BRD4 at these loci, as show in our BRD4 ChIP-Seq dataset following JQ1 treatment (Figs. 7B and S5). We have further tested the specificity of the S9.6 ChIP by pretreating nuclear lysates with RNase H to degrade R-loops prior to performing DRIP-qPCR and show that this results in a marked loss of the qPCR signal (Figs. 7C and S6, white bars), supporting the specificity of the S9.6 antibody at pulling-down R-loops as opposed to other RNA-containing structures. Using BRD4, γH2AX, and RNA pol II ser2 ChIP-Seq, we further map transcriptional downregulation and DNA damage through the gene bodies of BRD4, JMJD6, and CHD4 co-regulated genes that are responsible for R-loop suppression (Figs. 7B and S5, DHX9 and SRSF2) as well as transcriptional downregulation of genes that are known R-loop suppressors but have not previously been shown to be regulated by BRD4 (Figs. 7D and E, senataxin and SRSF1). This supports our model that inhibition of BRD4 leads to loss of R-loop suppression and the accumulation of R-loops, leading to increased transcription-replication conflicts and DNA damage (Fig. 10G). These results are now described in the new text highlighted on pages 13 to 15 of the revised manuscript.

2. The article uses comet assays and total g-H2AX staining intensity throughout to monitor DNA damage. While they do correlate the damage with S-phase, exploration of the damage response is quite limited. The authors might consider native BrdU immunofluorescence, or monitoring RPA2-S33 phosphorylation, and probing the ATR signaling pathway to really establish that DNA replication stress is arising from the BRD protein depletions.

We thank the reviewer again for this suggestion. We have now performed the requested native BrdU and RPA2-S33 phosphorylation immunofluorescence experiments in HeLa and HCT116 cells following bromodomain degradation with ARV-825 and inhibition with JQ1. As shown in Figures 8B and S8, both ARV-825 and JQ1 lead to increased BrdU (ssDNA) and RPA2-S33 (pRPA) immunostaining in cells, suggesting increased replication stress. This leads to increased fork stalling as demonstrated in Figures 8D-E and S7D-E using DNA fiber combing assays. Interestingly, BRD4 inhibition leads to paradoxic failed activation of the ATR-TopBP1-Chk1 replication stress checkpoint due to downregulation of TopBP1 expression by BRD4 (Figs. 10B-E and S9B-D), leading to increased death in S-phase cells (Fig. 10F, upper panel) and slippage of cells into mitosis leading to mitotic catastrophe (Figs. 10F, lower panel and S9E). These results are now described in the new highlighted text on page 17 and pages 19 to 20 of the revised manuscript.

3. I found it unfortunate that chemical inhibitors with multiple targets were used for all experiments except those in Figure 5 where siRNA was used to specifically implicate BRD4. It would be nice to at least repeat the triptolide experiments in the BRD4-siRNA setting, or probe the mechanism more deeply with the BRD4-siRNA reagents which would appear to be more specific than the inhibitors/degraders.

We thank the reviewer for this suggestion. We tried to perform the triptolide experiments in the BRD4 siRNA setting but found that our cell lines could not tolerate prolonged periods of triptolide treatment in the timeframe required for siRNA depletion of BRD4 (at least 48 hrs). To address the reviewers point, we therefore performed new experiments using induced RNase H expression in HeLa cells in the setting of BRD4 siRNA knockdown. This data, shown in Figures 4D-G, supports the mechanism in which removal of transcriptional roadblocks via degradation of R-loops by RNase H abrogates transcription-associated DNA damage that specifically follows BRD4 loss of function. As mentioned previously, we also performed genome-wide BRD4, γH2AX, and RNA pol II ser2 ChIP-Seq (Figs. 7A, B and S5) as well as DRIP-qPCR (Figs. 7C and S6) showing increased DNA damage and R-loop accumulation at loci that are co-regulated by BRD4, JMJD6, and CHD4, with transcription downregulation of known R-loop suppressors senataxin, DHX9, SRSF1, and SRSF2 following bromdomain inhibition (Figs. 7B, D-F, and S6). These data support a more specific role for BRD4 in regulating R-loop suppression and transcription-replication conflicts, and these results are described in the highlighted new text on pages 13 to 15 of the revised manuscript.

4. While the model of R-loop associated replication stress rests on considerable literature, the idea that 'deregulated transcription' leads to these events in BRD4-depleted cells is totally unclear. In the discussion the idea is raised that transcriptional pausing in the absence of BRD4 is increasing the opportunity of a nascent RNA to re-anneal with the template strand to create an R-loop. However, the authors

do not provide much data to support this view. As above, additional experiments targeting a specific locus that accumulates R-loops in BRD4 depleted cells for co-accumulation of paused RNA polymerase could be important.

The reviewer is correct, we did not present definitive experimental data showing transcriptional pausing, although this has been shown recently by others[18, 25, 28]. In fact, the new experimental data that we obtained following the reviewers' suggestions have greatly clarified the mechanism, as explained in our response to point 5 of reviewer 1, point 1 of reviewer 2, and point 1 of this reviewer.

As outlined in our response to point 1 above, and shown in Figures 7, S5, and S6, ChIP-Seq and DRIP-Seq experiments showed accumulation of R-loops and reduced RNA pol II ser2 present throughout the gene bodies of genes regulated by BRD4, BRD4 & JMJD6, and BRD4 & CHD4, not just at the promoter or transcription start site. Consequently, we have now removed all text in the discussion related to RNA Pol II pausing (at least as "pausing" was defined by Kevin Struhl[29]) as a mechanism for increased R-loop accumulation following bromodomain inhibition. Instead, our ChIP-Seq and DRIP-Seq results show that BRD4 inhibition leads to transcriptional downregulation and reduced elongation, as well as DNA damage through the entire gene bodies of BRD4-dependent genes, in excellent agreement with published data from Ozato and colleagues[7] and Chin and colleagues[8] that BRD4 plays a critical role in transcriptional elongation throughout the gene body by interacting with acetylated histones). In addition, we now show that genes whose transcription is affected by BRD4 loss include key known R-loop processing factors (senataxin, DHX9, SRSF1, and SRSF2), suggesting that the accumulation of R-loops might also result, in part, from loss of the R-loop suppression (Figs. 7B, D-F and S5). These results are discussed in the new text highlighted on pages 13 to 15 of the Results, and summarized in the new text highlighted on pages 22-24 of the revised manuscript.

5. In general a clearer statement of the proposed mechanism (i.e. what does transcriptional deregulation mean as a predisposing factor for R-loop induced DNA damage) and supporting data would significantly increase my enthusiasm for this study. There are many possible experiments that would be helpful and I don't mean to suggest that all avenues need to be explored, but testing some specific models is required.
For example, if RNA-seq data on transcriptional dysregulation after BRD protein depletion was available, then correlating these data with published R-loop maps might be instructive (i.e. if you predict that promoter-proximal pausing is driving R-loops then perhaps BRD4 targets would be R-loop prone). Alternatively, many groups have now had success using proximity ligation to show R-loop association in cells, or an increase in replication-transcription conflicts in situ.

Again, thank you. The revised manuscript now includes large amounts of new data and many additional experiments that allows us to make a clearer statement of the proposed mechanism based on the supporting data, as described in the response to point 4 above. We believe that our new data using BRD4, γH2AX, and RNA Pol II ser2 ChIP-Seq with corresponding DRIP-qPCR to identify a subset of known gene loci that are co-regulated by BRD4, JMJD6, and CHD4 (Fig. 7A), together with our demonstration that depletion of BRD4 and increased DNA damage (Figs. 7B and S5) correlates with accumulation of R-loops (Figs. 7C and S6) following bromodomain inhibition, and that the DNA damage is restricted to S-phase cells provides further evidence for the proposed mechanism of collisions between R-loops and the replication fork. To further support this mechanism we have added additional new data showing that bromodomain protein loss leads to increased markers of replication stress, namely increased native BrdU and RPA2 ser33 immunostaining in HeLa and HCT116 cells (Figs. 8B and S7B), causing increased replication fork stalling, as demonstrated in Figures 8D and S7D, with decreased incorporation of nucleotide analogues into DNA, and in Figures 8E and S7E, demonstrating decreased replication fork speeds, using DNA fiber combing assays. The addition of new cell cycle analysis in the absence and presence of induced-RNase H expression in HeLa cells partially restores normal cell cycle dynamics (Fig. 9C, right panel, + RNase H) and partially rescues the alterations in cellular proliferation following treatment with JQ1 (Fig. 9D, JQ1 + RNase H), further demonstrates that removal of transcriptional roadblocks by degradation of R-loops abrogates transcription-replication conflicts and collision-associated DNA damage. Finally, our ChIP-Seq and DRIP-qPCR findings show that DNA damage and R-loop formation after BRD4 inhibition occurs at gene loci of known R-loop suppressors, including DHX9 (Cristini et al, *Cell Reports*, 2018)[16] and SRSF2 (Chen et al, *Mol Cell*, 2018)[17], both of which are known to be regulated by BRD4 (Lui et al, *Cell*, 2013)[18], as well as at senataxin (Cohen et al, *Nat Commun*, 2018)[15] and SRSF1 (Cristini et al, *Cell Reports*, 2018)[16] loci, which have not been previously shown to be regulated by BRD4. Importantly we now show that this is accompanied by reduced protein levels of sentataxin, SRSF1, and DHX9 after JQ1 treatment (Figures 7D-F) providing a clearer potential mechanism for failure of R-loop suppression following BRD4 inhibition, leading to increased accumulation of R-loops and transcription-replication conflicts. These data are discussed in the new highlighted sections of the revised text.

Minor points:

The authors state that "Despite potent suppression of MYC, treatment with triptolide alone did not result in increased DNA damage signaling, DSBs, or apoptosis in cells…" Surely triptolide would eventually kill cells. I think the time frame of the experiment is what allows the observations with triptolide to exert the effects seen and that this should be acknowledged.

The reviewer is absolutely correct: prolonged exposure to RNA pol II inhibitors is quite toxic to almost all cells. The timing of this experiment is an important factor, and text to acknowledge this point has now been specifically added to the text, highlighted on pages 8 to 9 of the revised manuscript. In addition, we note that the timing of these triptolide experiments makes genetic experiments that more specifically target BRD4 difficult, as most conventional gene targeting strategies require 16-24 hours to accomplish gene loss of function, making triptolide co-treatment impossible.

Why is the time frame of ARV-825 treatment in Figure 3 (6 hours), different than in Figures 1 and 2 (3 hours)? It would seem that consistency between experiments or an explanation would be preferred.

We thank the reviewer for picking up on this inconsistency. In Figure 1, the blots showing BRD2, 3, and 4 protein loss after ARV-825 treatment (Figure 1E) were performed after 3 hrs of treatment to show rapid degradation. The immunofluorescence experiments showing increased γH2AX staining after ARV-825 treatment (Figure 1F), the comet assays (Figure 1G), and the initial onset of PARP cleavage (Figure 1H) were performed after 6 hrs of ARV-825 treatment because we reasoned that it might take some time after BRD4 loss for enough damage to occur to be detectable. In Figures 2 and 3, all of the ARV-825 treatments were for 6 hrs. This is now clarified in the Figures, figure captions, and relevant text.

The authors state in the discussion that "… it cannot be excluded that the RNA:DNA hybrid within the transcription bubble can remain annealed and be extended as RNAP moves forward." How likely do the authors think this is? RNAPII extrudes the nascent RNA through an exit tunnel that is physically displaced from the DNA template. This just seemed unlikely to be correct based on what we know about RNAPII.

The reviewer makes an excellent point. This statement has been removed from the revised manuscript.

REFERENCES

1. Floyd, S.R. et al. The bromodomain protein Brd4 insulates chromatin from DNA damage signalling. *Nature* **498**, 246-250 (2013).
2. Lam, F.C. et al. Enhanced efficacy of combined temozolomide and bromodomain inhibitor therapy for gliomas using targeted nanoparticles. *Nat Commun* **9**, 1991 (2018).
3. Pericole, F.V. et al. BRD4 Inhibition Enhances Azacitidine Efficacy in Acute Myeloid Leukemia and Myelodysplastic Syndromes. *Front Oncol* **9**, 16 (2019).
4. Zhang, J. et al. BRD4 facilitates replication stress-induced DNA damage response. *Oncogene* **37**, 3763-3777 (2018).
5. Maruyama, T. et al. A Mammalian bromodomain protein, brd4, interacts with replication factor C and inhibits progression to S phase. *Mol Cell Biol* **22**, 6509-6520 (2002).
6. Mochizuki, K. et al. The bromodomain protein Brd4 stimulates G1 gene transcription and promotes progression to S phase. *J Biol Chem* **283**, 9040-9048 (2008).
7. Kanno, T. et al. BRD4 assists elongation of both coding and enhancer RNAs by interacting with acetylated histones. *Nat Struct Mol Biol* **21**, 1047-1057 (2014).
8. Zhang, W. et al. Bromodomain-containing protein 4 (BRD4) regulates RNA polymerase II serine 2 phosphorylation in human CD4+ T cells. *J Biol Chem* **287**, 43137-43155 (2012).
9. Bowry, A., Piberger, A.L., Rojas, P., Saponaro, M. & Petermann, E. BET Inhibition Induces HEXIM1- and RAD51-Dependent Conflicts between Transcription and Replication. *Cell Rep* **25**, 2061-2069 e2064 (2018).
10. Wahba, L., Amon, J.D., Koshland, D. & Vuica-Ross, M. RNase H and multiple RNA biogenesis factors cooperate to prevent RNA:DNA hybrids from generating genome instability. *Mol Cell* **44**, 978-988 (2011).
11. Amon, J.D. & Koshland, D. RNase H enables efficient repair of R-loop induced DNA damage. *Elife* **5** (2016).
12. Castellano-Pozo, M. et al. R loops are linked to histone H3 S10 phosphorylation and chromatin condensation. *Mol Cell* **52**, 583-590 (2013).
13. Skourti-Stathaki, K., Proudfoot, N.J. & Gromak, N. Human senataxin resolves RNA/DNA hybrids formed at transcriptional pause sites to promote Xrn2-dependent termination. *Mol Cell* **42**, 794-805 (2011).
14. Sun, Q., Csorba, T., Skourti-Stathaki, K., Proudfoot, N.J. & Dean, C. R-loop stabilization represses antisense transcription at the Arabidopsis FLC locus. *Science* **340**, 619-621 (2013).
15. Cohen, S. et al. Senataxin resolves RNA:DNA hybrids forming at DNA double-strand breaks to prevent translocations. *Nat Commun* **9**, 533 (2018).
16. Cristini, A., Groh, M., Kristiansen, M.S. & Gromak, N. RNA/DNA Hybrid Interactome Identifies DXH9 as a Molecular Player in Transcriptional Termination and R-Loop-Associated DNA Damage. *Cell Rep* **23**, 1891-1905 (2018).
17. Chen, L. et al. The Augmented R-Loop Is a Unifying Mechanism for Myelodysplastic Syndromes Induced by High-Risk Splicing Factor Mutations. *Mol Cell* **69**, 412-425 e416 (2018).
18. Liu, W. et al. Brd4 and JMJD6-associated anti-pause enhancers in regulation of transcriptional pause release. *Cell* **155**, 1581-1595 (2013).
19. Chen, L. et al. R-ChIP Using Inactive RNase H Reveals Dynamic Coupling of R-loops with Transcriptional Pausing at Gene Promoters. *Mol Cell* **68**, 745-757 e745 (2017).
20. Rahman, S. et al. The Brd4 extraterminal domain confers transcription activation independent of pTEFb by recruiting multiple proteins, including NSD3. *Mol Cell Biol* **31**, 2641-2652 (2011).
21. Chudnovsky, Y. et al. ZFHX4 interacts with the NuRD core member CHD4 and regulates the glioblastoma tumor-initiating cell state. *Cell Rep* **6**, 313-324 (2014).
22. Itzen, F., Greifenberg, A.K., Bosken, C.A. & Geyer, M. Brd4 activates P-TEFb for RNA polymerase II CTD phosphorylation. *Nucleic Acids Res* **42**, 7577-7590 (2014).
23. Jang, M.K. et al. The bromodomain protein Brd4 is a positive regulatory component of P-TEFb and stimulates RNA polymerase II-dependent transcription. *Mol Cell* **19**, 523-534 (2005).
24. Schroder, S. et al. Two-pronged binding with bromodomain-containing protein 4 liberates positive transcription elongation factor b from inactive ribonucleoprotein complexes. *J Biol Chem* **287**, 1090-1099 (2012).

25. Winter, G.E. et al. BET Bromodomain Proteins Function as Master Transcription Elongation Factors Independent of CDK9 Recruitment. *Mol Cell* (2017).
26. Yang, Z. et al. Recruitment of P-TEFb for stimulation of transcriptional elongation by the bromodomain protein Brd4. *Mol Cell* **19**, 535-545 (2005).
27. Da Costa, D. et al. BET inhibition as a single or combined therapeutic approach in primary paediatric B-precursor acute lymphoblastic leukaemia. *Blood Cancer J* **3**, e126 (2013).
28. Anand, P. et al. BET bromodomains mediate transcriptional pause release in heart failure. *Cell* **154**, 569-582 (2013).
29. Wade, J.T. & Struhl, K. The transition from transcriptional initiation to elongation. *Curr Opin Genet Dev* **18**, 130-136 (2008).

Reviewers' comments:

Reviewer #2 (Remarks to the Author):

The authors have done an excellent job in addressing my previous concerns and the new data greatly improve the manuscript. However, the authors should explain how BET bromodomain protein loss leads to increased phosphorylation of RPA2 Ser33 (Figure 8B and S7B), while impairing the ATR-TopBP1-Chk1 pathway (Figure 10).

Reviewer #3 (Remarks to the Author):

The manuscript by Lam et al,Yaffe and Floyd has been revised with extensive new data, experimentation and validation. Many of these new experiments are in response reviewers suggestions (isoforms, cell cycle defects) and I appreciate the authors careful attempts to address our comments. One problem with this new data is that the manuscript is now dramatically extended in its scope which clouds the molecular mechanism and model that the authors are really proposing. While I appreciate the tremendous amount of work, the authors may be better to reframe this story in simpler terms. Below I will outline my persistent concerns.

The major issue that remains is that the article does not present a clear and cohesive model for how BRD4 prevents transcription-replication conflicts. The authors present models in Figure 7 and Figure 10 which incorporate their new observations that gene expression of key R-loop regulators is altered, leading to R-loops, TRCs and DNA damage. This is a perfectly reasonable model that I believe is likely to be true, but statements throughout the manuscript and inconsistencies in timing of their experiments undermine this clear message.

The paper should be simplified to focus on the mechanism of TRCs in BRD4 depleted cells and whether or not this is due to changes in gene expression alone, or whether there are direct physical effects of BRD4 on R-loops or the replisome.

- On Page 23, line 567, the authors still invoke RNAPII stalling as a short term mechanism of TRCs after BRD4 loss. If this is true and part of their model then they should experimentally support it or describe the literature in more detail.

- Loss of expression of SETX, DHX9 and SRSF1 is a great explanation for their observations, except that DNA damage and R-loops are seen after 6 hours of BRD4 depletion (Fig. 2 and 3) while SETX is depleted after 24 hours and DHX9 and SRSF1 are depleted after 48 hours (Figure 7). So this leaves a major gap in our understanding of the mechanism. If the early effects of failure to elongate transcription lead to R-loops and TRCs in cis to BRD4 sites then the article should focus on that rather than the late effects of gene expression. If there is a direct effect on all BRD4 target genes then the downregulation of SETX 18 hours later just confuses the issue. The same problem exists for the ATRIP/ATR/TOPBP1/CHK1 qPCR data which are done at 48 hours.

- The confusion around the proposed model is enforced by the wording in places. For example, on page 22 line 525-526 the authors state "… supports our model that a normal function of BRD4 in these cells is to suppress collisions between the transcription and replication machinery." I feel this invokes a very direct model not clearly articulated or supported by the authors. Based on the model where loss of R-loop interactor expression is the key driver, the function of BRD4 is just to regulate transcription not suppress collisions as indicated.

- The bottom of Page 15 also begins to invoke a kind of two-step model that BRD4 regulated genes form R-loops when BRD4 is lost, and that these genes themselves are R-loop regulators. Which of these R-loop promoting activities of BRD4-loss are important for the authors observations is not clear.

Other issues:
- I am not convinced by the data in 6B that only a subset of BRD4-loss-induced R-loops cause DNA damage. I doubt very much that the foci represent single R-loops that can be classified as with or without H2AX signal – there are 1000s of R-loop sites in the genome. It would be necessary to show BRD4-loss-induced R-loops that are damaging and BRD4-loss-induced R-loops that are not damaging to understand that a specific subset are damaged. I felt that this data was unnecessary.

- I did not understand the DNA combing data as presented. The authors inhibit BRD4 with JQ1 or ARV825 for 16 or 6 hours respectively, then they add IdU, then CldU for equivalent times. The fiber images show that IdU is incorporated normally, but that absolutely no CldU is incorporated. If replication is stalled after BRD4 inhibition, then neither label should be incorporated effectively. Or if there were more induced replisome stalling events, then the ratios of green to red track lengths should not equal 1. Given the representative images and the bar graph presentation, what the data actually shows is unclear and if the fibers shown are representative then there seems to be a technical problem.

Reviewer #2 (Remarks to the Author):

The authors have done an excellent job in addressing my previous concerns and the new data greatly improve the manuscript. However, the authors should explain how BET bromodomain protein loss leads to increased phosphorylation of RPA2 Ser33 (Figure 8B and S7B), while impairing the ATR-TopBP1-Chk1 pathway (Figure 10).

We thank the reviewer for this astute observation. There is now strong evidence suggesting that phosphorylation of RPA2 Ser33 occurs via activation of ATR through an ETAA1-ATR interaction and that this pathway is distinct from the ATR-TopBP1-Chk1 pathway required for replication stress-induced DNA damage checkpoint activation (Bass et al, *Nat Cell Biol*, **18** (11), pp. 1185-95, 2016; and Haahr et al, *Nat Cell Biol*, **18** (11), pp. 1196-1207, 2016)[1,2]. In Bass et al, the investigators report that they *"consistently observe that RPA phosphorylation is primarily dependent on ETAA1 and only modestly affected by TopBP1 knockdown"*. We can therefore infer that downregulation of TopBP1 following BET bromodomain protein loss would *not* affect RPA phosphorylation, which is dependent on ETAA1 activation of ATR, but *would* affect ATR activation of Chk1, which requires activation of ATR by TopBP1.

We have included this point on pp. 24-25 of the Discussion where we state: "Finally, it is interesting to observe phosphorylation of RPA2 ser33 despite our observed paradoxic decrease in the ATR-TopBP1-Chk1 pathway following BRD4 loss (Figs. 10D-E and S9C-D). There is now strong evidence suggesting that phosphorylation of RPA ser33 occurs through an ETAA1-ATR interaction which is independent of the activation of ATR by TopBP1. These studies show that phosphorylation of RPA is primarily dependent on ETAA1 and only modestly affected by TopBP1 knockdown. We can thus infer that there is an appropriate replication stress response to the increases in TRCs however cells cannot appropriately trigger the replication stress DNA damage checkpoint due to downregulation of TopBP1 following BRD4 loss."

Reviewer #3 (Remarks to the Author):

The manuscript by Lam et al, Yaffe and Floyd has been revised with extensive new data, experimentation and validation. Many of these new experiments are in response to reviewers' suggestions (isoforms, cell cycle defects) and I appreciate the authors careful attempts to address our comments. One problem with this new data is that the manuscript is now dramatically extended in its scope which clouds the molecular mechanism and model that the authors are really proposing. While I appreciate the tremendous amount of work, the authors may be better to reframe this story in simpler terms. Below I will outline my persistent concerns.

The major issue that remains is that the article does not present a clear and cohesive model for how BRD4 prevents transcription-replication conflicts. The authors present models in Figure 7 and Figure 10 which incorporate their new observations that gene expression of key R-loop regulators is altered, leading to R-loops, TRCs and DNA damage. This is a perfectly reasonable model that I believe is likely to be true, but statements throughout the manuscript and inconsistencies in timing of their experiments undermine this clear message.

The paper should be simplified to focus on the mechanism of TRCs in BRD4 depleted cells and

whether or not this is due to changes in gene expression alone, or whether there are direct physical effects of BRD4 on R-loops or the replisome.

- *On Page 23, line 567, the authors still invoke RNAPII stalling as a short term mechanism of TRCs after BRD4 loss. If this is true and part of their model then they should experimentally support it or describe the literature in more detail.*

We thank the reviewer for providing this insight. As requersted, in our revised discussion we now describe the literature detailing the role of BRD4 in both release of transcriptional pausing at proximal promoters during the transition from initiation to elongation, and the role of BRD4 in preventing stalling of RNAPII during elongation through its continued interaction with acetylated histones, citing appropriate references. Specifically, we now write on page 21 of the Discussion: "BRD4 performs at least 2 distinct functions during mRNA transcription: it facilitates the transition from transcriptional initiation to elongation (i.e. promoter proximal pause release), and it also prevents RNAPII stalling throughout elongation through its continual interaction with acetylated histones. BRD4 is known to release the PTEF-b complex from the inhibitory factors HEXIM1/2 and 7SK snRNA, allowing for its transition to its active form with subsequent phosphorylation of RNAPII at Ser2, as required for efficient transcriptional elongation[25,26,33,56,57,58]. Our data is also consistent with findings from Zhang et al, who showed that JQ1 treatment resulted in reduced RNAPII ser2 throughout the bodies of BRD4-regulated genes in CD4+ T-cells[26], and work by Liu et al, who showed decreased RNAPII travelling ratios (suggestive of RNAPII pausing) across RNAPII-bound genes, and decreased RNAPII ser2 across BRD4 regulated genes following BRD4 loss of function[59]. Importantly, however, work by Keiko Ozato's group has now shown that BRD4 is involved not only in facilitating pause-release at proximal promoters and the transition to transcriptional elongation at transcription start sites and enhancers, but also assists RNAPII progression throughout the gene bodies through interactions with acetylated histones via its bromodomains, independent of PTEF-b[25]. Furthermore, this process of BRD4-mediated passage of RNAPII through the body of transcriptionally elongating genes was directly antagonized by bromodomain inhibitors. Thus, BRD4 functions as an elongation factor required for RNA polymerase processivity all along the gene body, independently of its role at promoters[25]. Our experimental findings showing loss of productive RNAPII elongation, concomitant with increased RNA-loops and DNA damage throughout the gene bodies of a subset of BRD4-regulated genes following JQ1 treatment (Figs 7B-C, S5, and S6), is fully consistent with these models of BRD4 function from the literature.

- *Loss of expression of SETX, DHX9 and SRSF1 is a great explanation for their observations, except that DNA damage and R-loops are seen after 6 hours of BRD4 depletion (Fig. 2 and 3) while SETX is depleted after 24 hours and DHX9 and SRSF1 are depleted after 48 hours (Figure 7). So this leaves a major gap in our understanding of the mechanism. If the early effects of failure to elongate transcription lead to R-loops and TRCs in cis to BRD4 sites then the article should focus on that rather than the late effects of gene expression. If there is a direct effect on all BRD4 target genes then the downregulation of SETX 18 hours later just confuses the issue. The same problem exists for the ATRIP/ATR/TOPBP1/CHK1 qPCR data which are done at 48 hours.*

The reviewer is absolutely correct. While we do observe loss of R-loop resolving proteins after BRD4 inhibition, this effect is delayed relative to the initial observation of DNA damage, which occurs within 6 hours. We have therefore revised the data presentation, moving this data to the Supplemental Figures, and eliminated this from the revised model shown in Figure 10G. Loss of R-loop resolving

proteins could still contribute to the persistence or R-loops and DNA damage at later times, which we very briefly mention in the revised Discussion.

With regards to the downstream effects of BRD4 loss on the ATR/ATRIP/TopBP1/Chk1 axis, we would like to point out that the RT-qPCR data shown in Fig. 10B was obtained at both 24 *and* 48 hrs of JQ1 treatment to demonstrate the time-dependent downregulation of TopBP1 mRNA levels. The western blots in Figs. 10D and 10E were obtained after 24 hrs of JQ1 treatment. Karakashev and colleagues demonstrated potent downregulation of TopBP1 mRNA levels after *30 minutes* of JQ1 treatment in cells[3], further corroborating the early effects of bromodomain inhibition on transcription of TopBP1 in cells. While we stress that the inability to activity the ATR-TopBP1-Chk1 axis does not play into the mechanism of R-loop-associated DNA damage following BRD4 loss, we feel that it is important to demonstrate the paradoxic failure of the activation of the ATR-driven replication stress DNA damage checkpoint in response to transcription-replication collisions following prolonged BRD4 inhibition because this would offer valuable insight as to why cells are unable to repair their damaged DNA as well as help to further elucidate a mechanism of action for this class of small molecule inhibitors as a potential anti-cancer therapeutic.

- *The confusion around the proposed model is enforced by the wording in places. For example, on page 22 line 525-526 the authors state "… supports our model that a normal function of BRD4 in these cells is to suppress collisions between the transcription and replication machinery." I feel this invokes a very direct model not clearly articulated or supported by the authors. Based on the model where loss of R-loop interactor expression is the key driver, the function of BRD4 is just to regulate transcription not suppress collisions as indicated.*

The reviewer is again correct. We have therefore revised this statement on page 20 of the revised manuscript to state: "Our data showing that degradation (Figs. 8A & S7A) or inhibition (Fig. 10F) of BET bromodomain proteins in several oncogenic cell lines leads to increased DNA damage specifically during S-phase, supports our model that a normal function of BRD4 in these cells is to enhance transcription of RNAPII-bound genes, ensuring proper spatio-temporal co-ordination between the transcription and replication machinery". Thank you for catching this error in our thinking.

- *The bottom of Page 15 also begins to invoke a kind of two-step model that BRD4 regulated genes form R-loops when BRD4 is lost, and that these genes themselves are R-loop regulators. Which of these R-loop promoting activities of BRD4-loss are important for the authors observations is not clear.*

As outlined above, we have now removed the transcriptional regulation of R-loop processing proteins by BRD4 from the results section and have simplified the final model to focus on loss of RNAPII elongation following BRD4 loss leading to accumulation of R-loops and DNA damage at BRD4-regulated genes. We do briefly mention the late loss of R-loop resolving proteins in the Discussion for the sake of completeness.

Other issues:

- *I am not convinced by the data in 6B that only a subset of BRD4-loss-induced R-loops cause DNA damage. I doubt very much that the foci represent single R-loops that can be classified as with or without H2AX signal – there are 1000s of R-loop sites in the genome. It would be necessary to show*

BRD4-loss-induced R-loops that are damaging and BRD4-loss-induced R-loops that are not damaging to understand that a specific subset are damaged. I felt that this data was unnecessary.

We thank the reviewer for their interpretation of this figure. We also agree that the foci likely do not represent single R-loops but we felt that this data was a way of demonstrating that there is some degree of co-localization of our initial immunofluorescence data characterizing increased numbers of nuclear R-loops and γH2AX foci following BRD4 loss. This then led to our genome-wide ChIP-seq and DRIP-qPCR experiments showing damage at a subset of BRD4-regulated genes (Figures 7A, 7B, and S5) with R-loop accumulation throughout the entire gene body (Figures 7C and S6) following bromodomain inhibition. The senior editor at Nature Communications specifically liked that figure when she visited us at MIT, and we therefore respectfully wish to preserve it in the manuscript.

- I did not understand the DNA combing data as presented. The authors inhibit BRD4 with JQ1 or ARV825 for 16 or 6 hours respectively, then they add IdU, then CldU for equivalent times. The fiber images show that IdU is incorporated normally, but that absolutely no CldU is incorporated. If replication is stalled after BRD4 inhibition, then neither label should be incorporated effectively. Or if there were more induced replisome stalling events, then the ratios of green to red track lengths should not equal 1. Given the representative images and the bar graph presentation, what the data actually shows is unclear and if the fibers shown are representative then there seems to be a technical problem.

We thank the reviewer for this observation. We were initially also quite surprised at the lack of second label CldU incorporation into DNA fibers following treatment with JQ1 and ARV-825, but we repeated the experiments in two different cell lines (Fig. 8B in HeLa cells and Fig. S7D in HCT116 cells), and still observed the same results, which made us feel more confident that this was not due to a technical problem in nucleoside analog incorporation, fiber spreading, or antibody labeling of DNA fibers on the slides. We thus concluded that this was a true result of the effects of bromodomain protein inhibition or degradation on incorporation of the nucleoside analogues at the forks. Our results are fully supported by a recent report by Petermann and colleagues showing that short-term treatment of U2OS cells with 1 μM JQ1 for up to 8 hours leads to significant slowing of replication forks to ~ 0.6 kb/min compared to DMSO control and that this percent change in fork slowing is even more dramatic in other oncogenic cell lines including NALM6 leukemic and MEC1 B-cell lymphocytic leukaemia cells[4]. Finally, a study by Stankovic and colleagues completely supports our observations regarding lack of second analog incorporation following JQ1 treatment, since these authors also showed a lack of incorporation of the second nucleoside analogue label following treatment of NALM6 cells with 1 μM of JQ1 for 24 hrs (see figure below reporodiced from Figure 6a of Da Costa et al, *Blood Cancer Journal*, **3**, e126, 2013)[5]. This study further corroborates our findings that in the onset of fork slowing with JQ1 treatment that cells are still able to incorporate some of the first nucleoside analogue but this becomes more challenging upon addition of the second analogue.

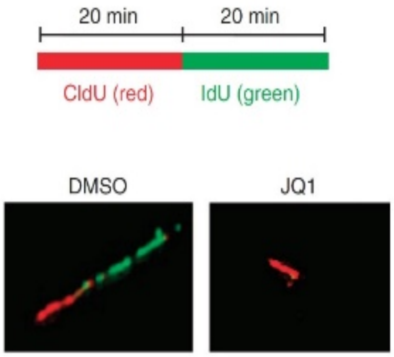

Figure 6A from Da Costa et al, *Blood Cancer Journal*, **3**, e126, 2013 shows that treatment of NALM6 leukemic cells with 1 μM of JQ1 for 24 hours followed by 20 minute consecutive pulses of CldU followed by IdU show failure to incorporate the second nucleoside analogue label in DNA fiber spreads.

References

1 Bass, T. E. *et al.* ETAA1 acts at stalled replication forks to maintain genome integrity. *Nat Cell Biol* **18**, 1185-1195, doi:10.1038/ncb3415
ncb3415 [pii] (2016).

2 Haahr, P. *et al.* Activation of the ATR kinase by the RPA-binding protein ETAA1. *Nat Cell Biol* **18**, 1196-1207, doi:10.1038/ncb3422
ncb3422 [pii] (2016).

3 Karakashev, S. *et al.* BET Bromodomain Inhibition Synergizes with PARP Inhibitor in Epithelial Ovarian Cancer. *Cell Rep* **21**, 3398-3405, doi:S2211-1247(17)31762-X [pii]
10.1016/j.celrep.2017.11.095 (2017).

4 Bowry, A., Piberger, A. L., Rojas, P., Saponaro, M. & Petermann, E. BET Inhibition Induces HEXIM1- and RAD51-Dependent Conflicts between Transcription and Replication. *Cell Rep* **25**, 2061-2069 e2064, doi:S2211-1247(18)31683-8 [pii]
10.1016/j.celrep.2018.10.079 (2018).

5 Da Costa, D. *et al.* BET inhibition as a single or combined therapeutic approach in primary paediatric B-precursor acute lymphoblastic leukaemia. *Blood Cancer J* **3**, e126, doi:10.1038/bcj.2013.24
bcj201324 [pii] (2013).
